# Synthesis of spiro quasi[1]catenanes and quasi[1]rotaxanes via a templated backfolding strategy

**DOI:** 10.1038/ncomms15392

**Published:** 2017-05-25

**Authors:** Luuk Steemers, Martin J. Wanner, Martin Lutz, Henk Hiemstra, Jan H. van Maarseveen

**Affiliations:** 1 Van ‘t Hoff Institute for Molecular Sciences, University of Amsterdam, Science Park 904, Amsterdam 1098 XH, The Netherlands; 2 Crystal and Structural Chemistry Bijvoet Center for Biomolecular Research, Utrecht University, Padualaan 8, Utrecht 3584 CH, The Netherlands

## Abstract

Due to their well-defined three-dimensional geometry, spiro compounds are widely utilized in drug research. From the central tetrahedral carbon atom, besides the regular structure, an inverted spiro connectivity may be envisioned. Here we disclose the synthesis of this molecule class that we have coined quasi[1]catenanes. Next to their fascinating and aesthetic shape, the higher compactness as compared to regular spiro bicycles is noteworthy. To enable synthetic access to compact entangled multimacrocyclic molecules, we have developed a new strategy. The key element is a template, which is covalently connected to the linear precursors, and spatially directs the sterically congested backfolding macrocyclizations that are required to give quasi[1]catenanes. Similarly, quasi[1]rotaxanes are made.

## Abstract (web summary)

Spiro compounds contain two or more rings linked together through one common atom. Here the authors provide a method to backfold both rings, producing spiro quasi[1]catenanes, via a strategy of temporarily linking the linear intermediates with covalent bonds.

Entangled molecular architectures inspire synthetic chemists for creative endeavours and find application in nanotechnology and materials research^1^. Striking natural examples are the lasso peptides^2^, and also catenane structures found in some proteins^3^ and DNA^4^. Synthetically, an impressive array of entangled molecules such as catenanes and rotaxanes have been made that are mainly applied in nanotechnology research^1^. Supramolecular three-dimensional templating via metal coordination, π-stacking or hydrogen bonding as developed by the respective Sauvage^5^/Leigh^6^, Stoddart^7^,^8^ and Hunter^9^/Vögtle^10^ groups gave access to the vast majority of the catenane and rotaxane series^11^. However, the prerequisite of moieties able to form non-covalent interactions limits the structural diversity of the entangled molecules that can be obtained. Remarkably, the first example of a non-statistical [2]catenane synthesis was reported back in 1964 by Schill and Lüttringhaus^12^ using a covalent approach^12^,^13^,^14^. Over the past years, there has been a comeback of covalent approaches towards entangled structures^15^,^16^,^17^,^18^,^19^. Currently, we are developing covalent template-directed synthetic concepts to disclose unknown or ‘impossible'^20^ entangled or mechanically interlocked molecular geometries with access to the natural [1]rotaxane-type lasso peptide series as the ultimate goal^21^. Here we report a synthetic concept based on template-directed backfolding macrocyclization of which the utility is demonstrated by the efficient synthesis of an uncommon spiro geometry^22^. The regular spiro geometry is obtained after connection of two rings to the same core tetrahedral carbon atom and can be considered as the molecular equivalent of the figure of eight^23^. The fascinating spirocyclic geometry is found in many natural compounds and due to their rigid and well-defined three-dimensional shape, characterized by a perpendicular arrangement of the two rings, spiro compounds find wide application in drug research^24^. Besides the regular geometry (as for structure **1**, Fig. 1), an alternative connectivity may be envisioned by backfolding of the two rings at the central tetrahedral carbon atom to give an inverted spiro configuration (that is, **2**, Fig. 1). An intermediate in the landmark [2]catenane synthesis by the Schill group already displayed a similar inverted spiro geometry^12^. Despite the fact that both spiro geometries do not contain any stereocentre, they are still interesting from a stereochemical point of view. Uniquely, in the spiro connectivity case, after inverting the centre-of-symmetry tetrahedral carbon, a diastereomer is obtained with different properties while retaining the C_2_ symmetry around the same axis. Although we realize that every name is a compromise because no mechanical bond is present, we coined the topologically trivial inverted spiro geometry a quasi[1]catenane **2** (ref. 25). Similarly, a quasi[1]rotaxane **3** can be drawn, of which unwinding to give conformer **4** is prevented by large stoppers (Fig. 1).

For steric reasons, the synthesis of the quasi[1]catenane **2** and quasi[1]rotaxane **3** architectures requires specifically tailored methodologies. We have tackled this challenge, as will be pointed out first for quasi[1]catenane **2**, by using a covalent template directing the required backfolding cyclization. Template **6** (Fig. 2, route A) will be temporarily connected to two of the four linear ring precursors in **5** and will eventually be part of the spirocyclic ring formed with the other precursor chains. Due to the tetrahedral geometry of the central carbon atom, cyclization of the first ring on the template in **7** will occur in a perpendicular and backfolded fashion over the linear precursor of the second ring to form a pseudo-quasi[1]rotaxane **8** (ref. 26). Subsequent backfolding cyclization of the second ring to give the inverted spiro connectivity, followed by cleavage of the temporal scaffolding bonds provides bicyclic quasi[1]catenane **2**. Essentially, spiro quasi[1]catenane **2** may be obtained from the tetrahedral precursor **5** in just four steps, of which three are macrocyclizations^27^. Without making the temporal connections to the template, the regular spiro bicycle **1** will be obtained (Fig. 2, routes B and C). An alternative route to the regular spiro bicycle **1** opens up after breaking the temporary bonds in **8** (Fig. 2, route D), initiating unwinding of the sterically congested quasi-thread fragment to give **10**, followed by closure of the second ring. Capping the end-groups in pseudo-quasi[1]rotaxane **8** by large stoppers (Fig. 2, route E) to give **12** prevents the unwinding process after temporal bond cleavage to give quasi[1]rotaxane **3**. Stopper attachment to **10** (Fig. 2, route F) gives the sterically relaxed conformer **4**.

## Results

### Design of the building blocks

To avoid the requirement of protective groups and to facilitate the sterically difficult macrocyclizations, robust transformations were selected, that is, transesterification/lactonization^28^ for the scaffolding temporal connection to the template, Cu-catalysed azide alkyne cycloaddition^29^,^30^ (CuAAC) for the first ring closure and olefin metathesis^31^ for the second and final ring closure or introduction of the quasi-rotaxane stopper elements. For synthetic reasons and to ensure the perpendicular mutual arrangement of the two pairs of ring-precursor chains at precursor **5**, we have chosen a spiro-linkage via the tetrahedral carbon atom of a 9*H*-fluorene moiety (Fig. 3)^32^. The design of the temporal covalent scaffolding tether at the central precursor **5** is based on amide-*N*-benzylic moieties containing phenolic hydroxyl groups that will be esterified to template **6** and may be removed from the final spiro or rotaxane compounds by consecutive transesterification and protolytic cleavage of the amide benzylic linkages^33^. As the template, 2,5-bis(pent-4-yn-1-yloxy)-1,4-benzenedicarboxylic ester **6** was chosen. The tether length in precursor **5** and template **6** gives access to spiro architectures of 27- and 31-membered rings, resulting from CuAAC and ring-closing metathesis (RCM) macrocyclizations, respectively. These ring sizes are significantly larger than the smallest accessible rotaxane ring fragment of 21 atoms and in the same range as the natural peptide rotaxanes^34^. For mechanically locking the conformation of the 31-membered ring of the quasi[1]rotaxane by cross-metathesis, we selected tris(4-(*tert*-butyl)phenyl)methane-functionalized acrylamide **11** as the stopper element^35^. For the multigram scale synthesis of the central tetrahedral precursor **5**, template **6** and rotaxane stopper **11**, please see the Supplementary Information.

### Synthesis of the quasi[1]catenane

**Figure 2:**
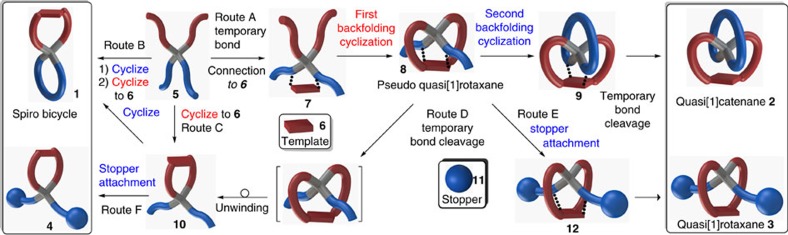
The template-directed backfolding concept. The synthesis of quasi[1]catenane **2** starts by temporal connection of **5** to template **6** providing **7** (route A). This temporary connection directs the first backfolding cyclization to the template to give **8**. A second backfolding cyclization provides **9** and temporary template-connection cleavage liberates the quasi[1]catenane **2**. Besides the direct routes B and C, the regular spiro compound **1** may also be obtained after template-connection cleavage in **8**, inducing unwinding, followed by the final cyclization (route D). Capping of **8** by bulky stoppers **11** followed by template cleavage provides quasi[1]rotaxane **3** (route E). Capping of monocycle **10** gives the unwound conformer **4** (route F).

**Figure 3:**
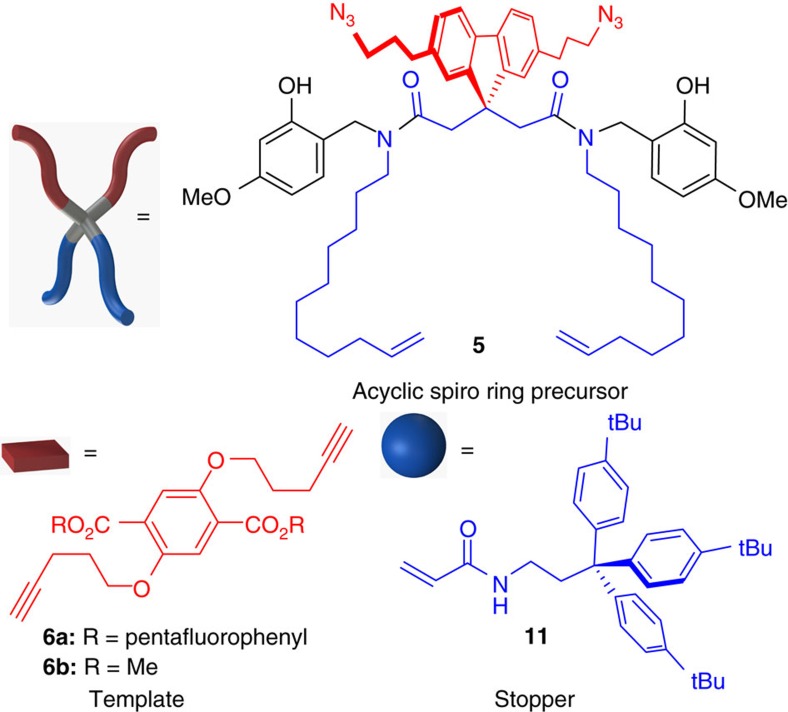
Molecular structures of the key components. Pivotal to the design of central precursor **5** is a tricyclic fluorene moiety containing the tetrahedral carbon atom that ensures a perfect perpendicular arrangement of the acyclic fragments. The two amide groups contain acid-cleavable phenolic benzyl groups allowing temporary connection via transesterification to template **6**. The terminal alkene and azides/alkynes allow robust RCM and CuAAC reactions towards the macrocyclic spiro geometry **1** and quasi[1]catenane **2** or stopper **11** attachment via cross-metathesis to give quasi[1]rotaxane **3** and its unwound conformer **4**.

**Figure 4:**
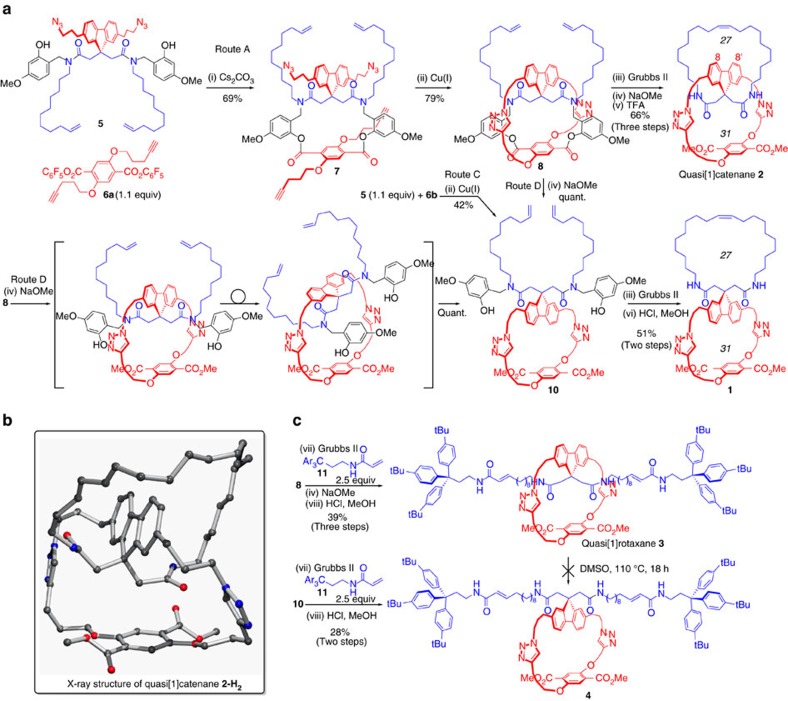
Synthetic routes from the common quaternary carbon precursor 5. (**a**) Detailed reaction conditions for the synthesis of spiro bicycle **1** and quasi[1]catenane **2**: (i) Cs_2_CO_3_ (4 equiv), MeCN (2 mM), 4 Å molecular sieves, 60 °C. (ii) Cu(MeCN)_4_BF_4_ (0.25 equiv), TBTA (0.25 equiv), CH_2_Cl_2_ (5 mM), reflux. (iii) Grubbs second-generation catalyst (0.2 equiv), CH_2_Cl_2_ (1 mM), 30 °C. (iv) NaOMe (5 equiv), MeOH/THF (1:1). (v) TFA/CH_2_Cl_2_ (9:1), Et_3_SiH (20 equiv). (vi) HCl (3 M), MeOH. (**b**) Molecular structure of quasi[1]catenane **2-H**_**2**_ in the crystal. Hydrate water and the disorder present in the C20 aliphatic chain have been removed in the drawing. (**c**) Detailed reaction conditions for the synthesis of quasi[1]rotaxane **3** and its unwound conformer **4**: (vii) Grubbs second-generation catalyst (0.2 equiv), CH_2_Cl_2_ (5 mM), reflux. (viii) HCl (3 M), MeOH/THF (4:1). Further conditions see **a**. TBTA=tris[(1-benzyl-1*H*-1,2,3-triazol-4-yl)methyl]amine, Grubbs second-generation catalyst=(1,3-Bis(2,4,6-trimethylphenyl)-2-imidazolidinylidene)dichloro-(phenylmethylene)(tricyclohexylphosphine)ruthenium.

We first followed the strategy to quasi[1]catenane **2** incorporating the inverted spiro[27,31] framework (Fig. 2, route A). Connection of **5** via transesterification of the pentafluorophenol ester **6a** using Cs_2_CO_3_ as the base in acetonitrile (2 mM) gave the 21-membered macrocyclic bislactone **7** in 69% isolated yield (Fig. 4a). The subsequent backfolding double CuAAC reactions required careful optimization to avoid competing intermolecular reactions over the challenging process to the 31-membered cage-type macrocycle^36^. Slow addition of a 5 mM solution of **7** to a solution of CuI (2 equiv) and DIPEA/lutidine (4 equiv) in acetonitrile gave **8** in poor and irreproducible yields due to extensive insoluble polymer formation. Simply stirring a 5 mM dichloromethane solution of **7** at reflux for 24 h using tetrakis(acetonitrile)copper(I)tetrafluoroborate (0.25 equiv) and tris[(1-benzyl-1*H*-1,2,3-triazol-4-yl)methyl]amine (TBTA, 0.25 equiv) as the catalytic complex gave, after work-up and purification, **8** in a 79% reproducible yield^37^. The final backfolding closure of the 27-membered cycloalkene via RCM using Grubbs' second-generation catalyst at high dilution (1 mM) in dichloromethane at reflux gave the tricyclic product in an isolated yield of 71% as a mixture of E/Z isomers. As a coeluting side product, the liquid chromatography mass spectrometry trace also showed some presence of the 26-membered cycloalkene analogue, due to double bond migration before cyclization^38^. After removal of the temporary tether by consecutive transesterification using NaOMe and trifluoroacetic acid (TFA)/Et_3_SiH-mediated protolytic cleavage of the benzylic linkages, the quasi[1]catenane **2** was isolated in a last-three-steps yield of 66%. ^1^H NMR studies confirmed the solution phase C2-symmetric conformation of the inverted spiro quasi[1]catenane **2** as drawn in Fig. 4a. Most protons of the nine pairs of cycloalkene methylene protons gave well-separated signals. In addition, the strong nOe contacts between the fluorene 8 and 8′ protons and the majority of the aliphatic protons of the macrocycloalkene confirm the folding of the macrocycle over the fluorene moiety (see Supplementary Information). Fortunately, after catalytic hydrogenation to remove the alkene E/Z mixture, crystals of **2-H**_**2**_ could be obtained of sufficient quality for X-ray diffraction (Fig. 4b). Besides unequivocal proof of the backfolded quasi[1]catenane configuration, the solid-state structure showed a similar conformation as in solution, albeit with the flexible C20-macrocycloalkane offset folded over the fluorene moiety.

**Figure 5:**
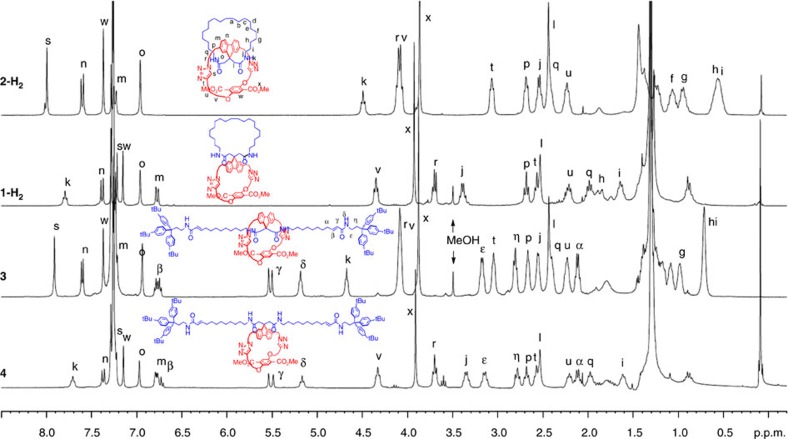
^1^H NMR spectra. Overlays of the ^1^H NMR spectra clearly show the similarities between the geometries of quasi[1]catenane **2-H**_**2**_ and quasi[1]rotaxane **3**, and also between spiro bicycle **1-H**_**2**_ and monocycle **4**. Furthermore, the structural dissimilarity between both C_2_-symmetric but diastereomeric spiro bicycle **1** and quasi[1]catenane **2-H**_**2**_ is reflected by their spectra as is the case for quasi[1]rotaxane **3** and its unwound conformer **4**.

Next, the regular spiro[27,31] macrobicycle **1** was made. Ironically, initial attempts were troublesome. Subjection of **5** to the high-dilution RCM macrocyclization conditions (Fig. 2, route B) led to complex and inseparable mixtures. Fortunately, by beginning with the CuAAC reaction from **5** and **6b**, monocycle **10** was obtained, although in a yield of 42% only (Fig. 4a, route C). It should be noted that this yield is significantly lower than the CuAAC reactions to **8** that both occurred in a template/scaffold-preorganized intramolecular fashion. As an elegant detour, monocycle **10** could be obtained quantitatively after methoxide-induced cleavage of the tether in pseudo-quasi[1]rotaxane **8** inducing immediate steric relaxation (Fig. 4a, route D). By lowering the temperature to 30 °C at high dilution, the RCM reaction proceeded cleanly and no truncated cycloalkene was observed. Protolytic removal of the 2-hydroxy-4-methoxybenzyl groups led to an unexpectedly stable adduct of TFA and **1**, which could not be separated. However, treatment with methanolic 3 M HCl at 50 °C gave a clean removal of the benzyl groups and the resulting triazolium ions were neutralized with aqueous NaHCO_3_ to give the regular spiro macrocycle **1** in a yield of 51%. Catalytic hydrogenation removed the RCM-derived olefin E/Z mixture to give **1-H**_**2**_. The different geometries of **1-H**_**2**_ and **2-H**_**2**_ are obvious from the ^1^H NMR spectra (Fig. 5) showing almost no overlapping signals. In quasi[1]catenane **2** especially, the four pairs of methylene protons (Fig. 5, protons f–i) experience an upfield shift to the 0.5 p.p.m. region due to their presence in the shielding region above the plane of the aromatic fluorene phenyl groups. For the same reason, a dramatic upfield shift of over 3 p.p.m. of the NH triplets (Fig. 5, protons k) of **2** is observed. The inverted spiro geometry in **2** is also reflected by the hydrogen bonds between the triazole CH's (Fig. 5, protons s) and the amide carbonyls resulting in a 0.8 p.p.m. downfield shield as compared to the same protons in regular spiro compound **1**. In addition, donor–acceptor distances in the crystal structure of 2.2 and 2.4 Å, respectively, show these hydrogen bonds in the solid state of **2**. The different chromatographic retention times of the two spiro diastereomers visualizes the different physical properties showing a higher polarity for the densely packed quasi[1]catenane **2**.

### Synthesis of the quasi[1]rotaxane

The backfolded intermediate **8** also provides access to quasi[1]rotaxane **3** (Fig. 4c). Cross-metathesis^39^ of the terminal thread alkenes in **8** with the acrylamide-functionalized stopper **11**, followed by tether detachment by consecutive transesterification and protolysis gave quasi[1]rotaxane **3** in a yield of 39% over three steps. The unthreaded conformation **4** was obtained via cross-metathesis-mediated stopper attachment to unwound intermediate **10** and protolytic debenzylation in a two-step yield of 28%. The structural dissimilarity between the quasi[1]rotaxane **3** and conformer **4** was reflected by comparing the ^1^H NMR spectra showing, as in the quasi[1]catenane series, remarkable shift differences, especially for the methylene protons in the thread fragment close to the fluorene moiety, the central amide NH's and the triazole proton (Fig. 5). Quasi[1]rotaxane **3** and the unwound isomer **4** only differ in conformation of which interconversion is mechanically blocked by the stoppers. In contrast to the very facile unwinding of the pseudo-thread in **8** through the 31-membered ring to give **10** (Fig. 4a, route D), the tris(4-(*tert*-butyl)phenyl)methane stoppered quasi[1]rotaxane **3** conformation was completely stable, even after heating in dimethylsulphoxide at 110 °C for 18 h as was confirmed by ^1^H NMR spectroscopy.

## Discussion

Generally, spiro compounds are obtained after two ring closures of the four linear-chain ring precursors connected to a central tetrahedral carbon atom. We have shown that after prior temporal covalent connection via a template of two of the four ring-precursor chains, ring closure of the second pair to the template is forced in a backfolded fashion. Final closure of the first pair of ring-precursor chains followed by cleavage of the temporary linkers to the template provides an inverted spiro geometry coined here as a quasi[1]catenane. Although the regular spiro and quasi[1]catenane geometries only differ by inversion of the configuration of the centre-of-symmetry tetrahedral carbon, they relate as diastereomers with inherently different physical properties as reflected by their contrasting ^1^H NMR spectra. By starting from the same tetrahedral carbon-centred acyclic precursor and the same backfolding concept, the structurally related mechanically locked quasi[1]rotaxane and its unwound conformer were obtained. The next step in the backfolding cyclization concept will be replacing the permanent perpendicular spiro connectivity by structurally similar but cleavable moieties, such as a 1,3-dioxolane, to give [2]rotaxanes and [2]catenanes. The longer-term goal is to apply the covalent tether-directed backfolding cyclization concept to synthetically disclose the natural [1]rotaxane-type lasso peptide series by incorporation of two amides of the thread section of the peptide backbone into a imidazolidin-4-one as the perpendicular and cleavable thread/ring connecting moiety. Covalent linking in a perpendicular fashion of the ring over thread fragment, as is the case with the backfolding cyclization concept, ensures the atom-precise mutual positioning of these components, which is a prerequisite for the eventual total synthesis of the lasso peptides.

## Methods

### Macrolactonization of the tetrahedral precursor **5** to template **6a**

**5** (1.94 g, 1.89 mmol), **6a** (1.38 g, 2.08 mmol, 1.1 equiv), Cs_2_CO_3_ (2.45 g, 7.58 mmol, 4 equiv) and 4 Å molecular sieves (1.00 g) were dissolved in dry CH_3_CN (750 ml) and the mixture was stirred overnight at 60 °C under a N_2_ atmosphere. The solvent was evaporated and the residue was taken in ca. 20 ml CH_2_Cl_2_ and filtered through a plug of Celite, which was washed with CH_2_Cl_2_. The organic layer was concentrated *in vacuo* and dry-loaded on silica and purified by column chromatography (petroleum ether (PE):EtOAc 4:1→3:1) to give **7** (1.72 g, 1.31 mmol, 69%) as a thick colourless oil.

### Double CuAAC macrocyclizations towards cage 8

**7** (420 mg, 0.319 mmol) and TBTA (42 mg, 0.080 mmol, 0.25 equiv) were dissolved in 65 ml dry CH_2_Cl_2_ and degassed with 5 vacuum/N_2_ cycles, after which Cu(CH_3_CN)_4_BF_4_ (25 mg, 0.080 mmol, 0.25 equiv) was added and the mixture was stirred overnight at reflux under N_2_ atmosphere. The reaction mixture was concentrated in *vacuo* and dry-loaded on silica and purified by column chromatography (PE:EtOAc 1:1→1:2→0:1) to give **8** (333 mg, 0.253 mmol, 79%) as a colourless foam.

### Ring-closing metathesis to the pseudo-quasi[1]catenane

**8** (79 mg, 0.060 mmol) was dissolved in dry CH_2_Cl_2_ (60 ml) and degassed with 5 vacuum/N_2_ cycles. To the solution was added Grubbs second-generation catalyst (10 mg, 0.012 mmol, 0.2 equiv) and the mixture was stirred overnight at 40 °C. The mixture was concentrated in *vacuo*, dry-loaded on silica and purified by column chromatography (PE:EtOAc 1:2→1:3→1:4) to give the pseudo-quasi[1]catenane (55 mg, 0.0427, mmol, 71%) as a beige solid.

### Temporary tether removal to liberate quasi[1]catenane 2

The pseudo-quasi[1]catenane (55 mg, 0.043 mmol) was dissolved in dry THF/CH_3_OH (2 ml, 1:1) and anhydrous NaOCH_3_ (12 mg, 0.21 mmol, 5 equiv) was added and the mixture was stirred at room temperature for 1 h. The reaction was quenched by addition of 0.5 ml AcOH and the reaction was diluted with 15 ml EtOAc and 10 ml saturated NaHCO_3_. The water layer was extracted with 2 × 5 ml EtOAc and the combined organic layers were dried over MgSO_4_ and concentrated in *vacuo* to give the dimethyl ester (58 mg, 0.0427, mmol, quantitative) as a slight yellow film. To this, TFA/CH_2_Cl_2_ (5 ml, 9:1) and Et_3_SiH (0.136 ml, 0.854 mmol, 20 equiv) were added. The mixture was stirred overnight at room temperature and concentrated in *vacuo*. The residue was dissolved in CH_2_Cl_2_ (10 ml) and Et_3_N (0.5 ml) was added and stirred for 5 min. The organic layers were washed with 1 M HCl (10 ml), saturated NaHCO_3_ (10 ml), dried over MgSO_4_ and concentrated in *vacuo*. The crude product was dry-loaded on silica and purified by column chromatography (CH_2_Cl_2_/CH_3_OH 96:4→94:6) to give **2** (43 mg, 0.040 mmol, 93%) as a colourless foam.

### Data availability

Additional data supporting the findings reported in this article are available within the Supplementary Information file. For the experimental procedures and spectral data of all compounds described, see the Supplementary Methods. For ^1^H and ^13^C NMR spectra of all compounds, see Supplementary Figs 5–71. CCDC 1517772 contains the supplementary crystallographic data for compound **2-H**_**2**_. These data can be obtained free of charge from the Cambridge Crystallographic Data Centre via www.ccdc.cam.ac.uk/data_request/cif.

## Additional information

**How to cite this article:** Steemers, L. *et al*. Synthesis of spiro quasi[1]catenanes and quasi[1]rotaxanes via a templated backfolding strategy. *Nat. Commun.* **8,** 15392 doi: 10.1038/ncomms15392 (2017).

**Publisher's note**: Springer Nature remains neutral with regard to jurisdictional claims in published maps and institutional affiliations.

## Supplementary Material

Supplementary Information Supplementary figures, supplementary methods and supplementary references.

## Figures and Tables

**Figure 1:**
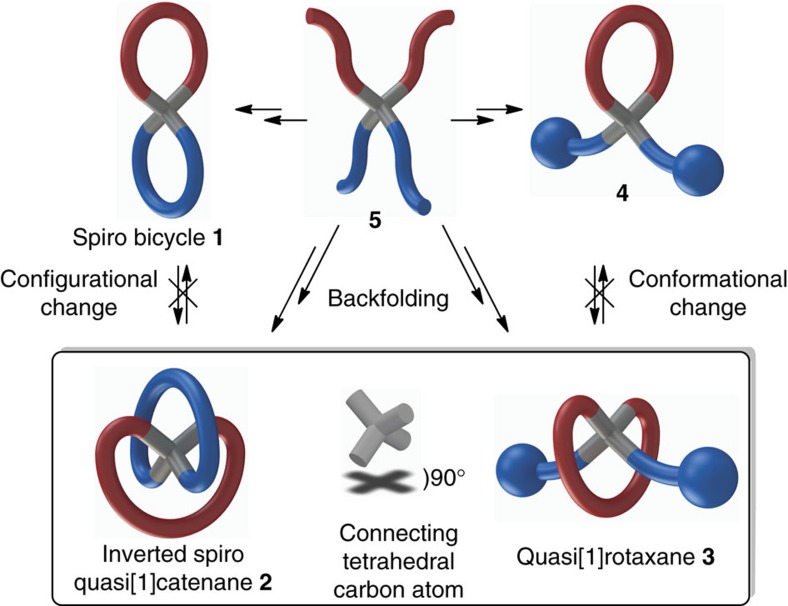
Sketches of the quasi[1]catenane and quasi[1]rotaxane geometries. These are, together with the two regular ring systems, all available from a single quaternary carbon precursor. By starting from compound **5** in which four linear-chain ring precursors are connected to the same central tetrahedral carbon atom, after closure of the perpendicularly arranged rings, two spiro geometries may be envisioned. Besides the regular spiro bicycle **1**, after double-backfolding ring closure, the inverted spiro configuration is obtained giving quasi[1]catenane **2**. Alternatively, by installation of large stoppers at the end of the two linear chain fragments, next to the regular closure to give **4**, cyclization via backfolding gives access to the mechanically locked quasi[1]rotaxane **3** conformation.
