# Supplementary material for: Synthesis of spiro quasi[1]catenanes and quasi[1]rotaxanes via a templated backfolding strategy
Source: Nat Commun. 2017 May 25;8:15392. doi: 10.1038/ncomms15392 (PMC5458513; doi:10.1038/ncomms15392)
Supplement: Supplementary Information — Supplementary figures, supplementary methods and supplementary references. [file ncomms15392-s1.pdf]

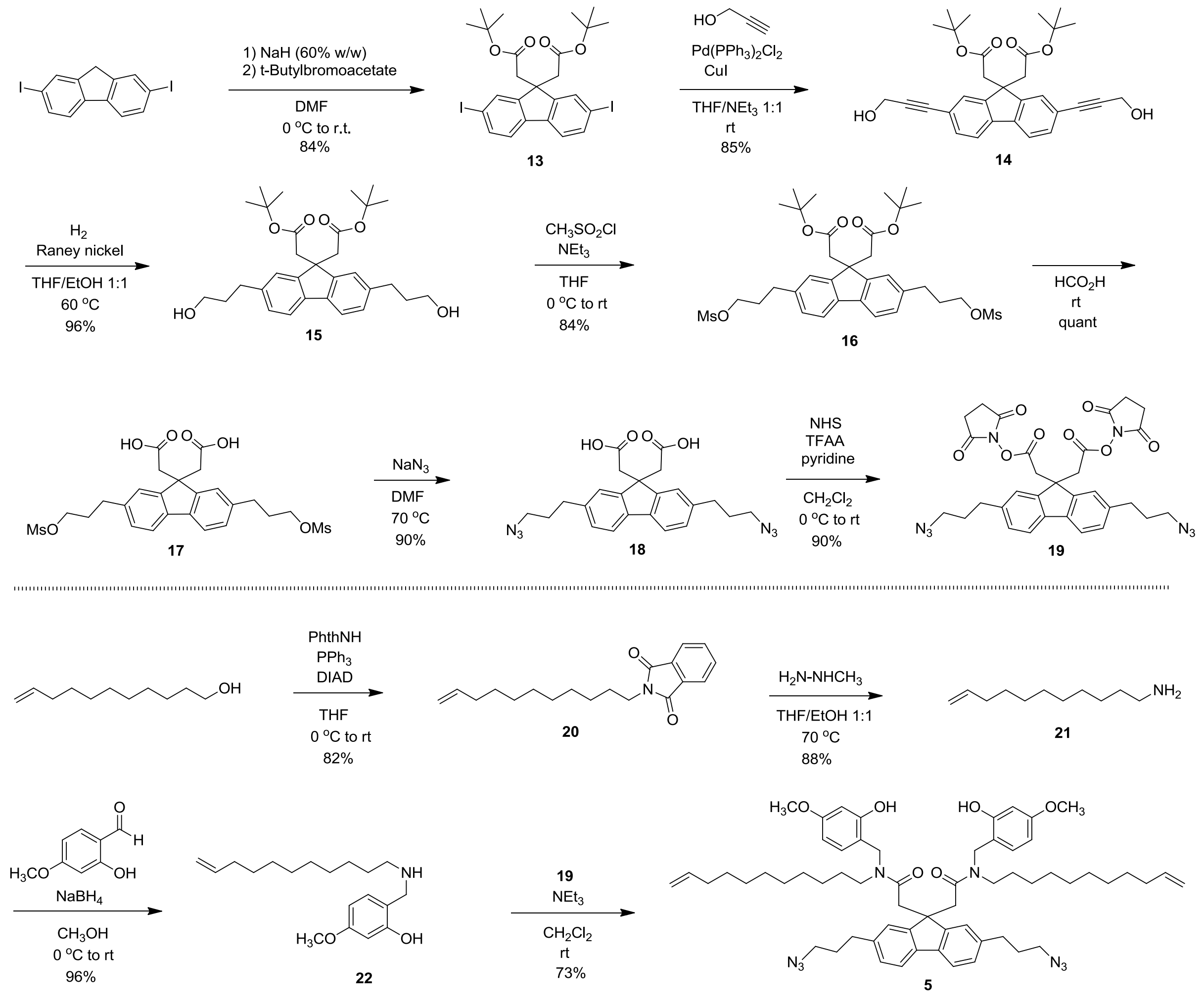

Supplementary figure 1. Synthesis of compound 5

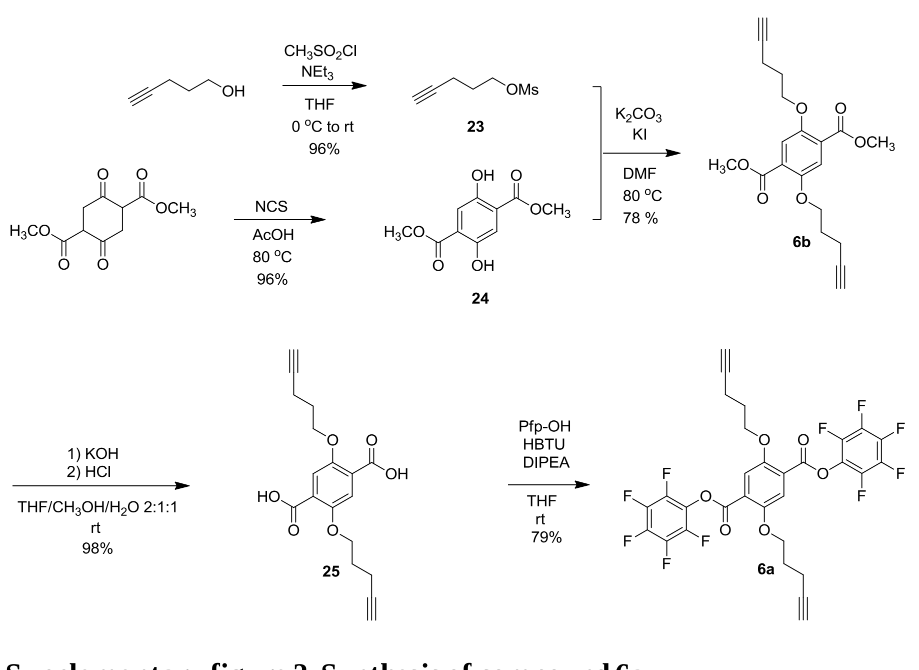

Supplementary figure 2. Synthesis of compound 6a

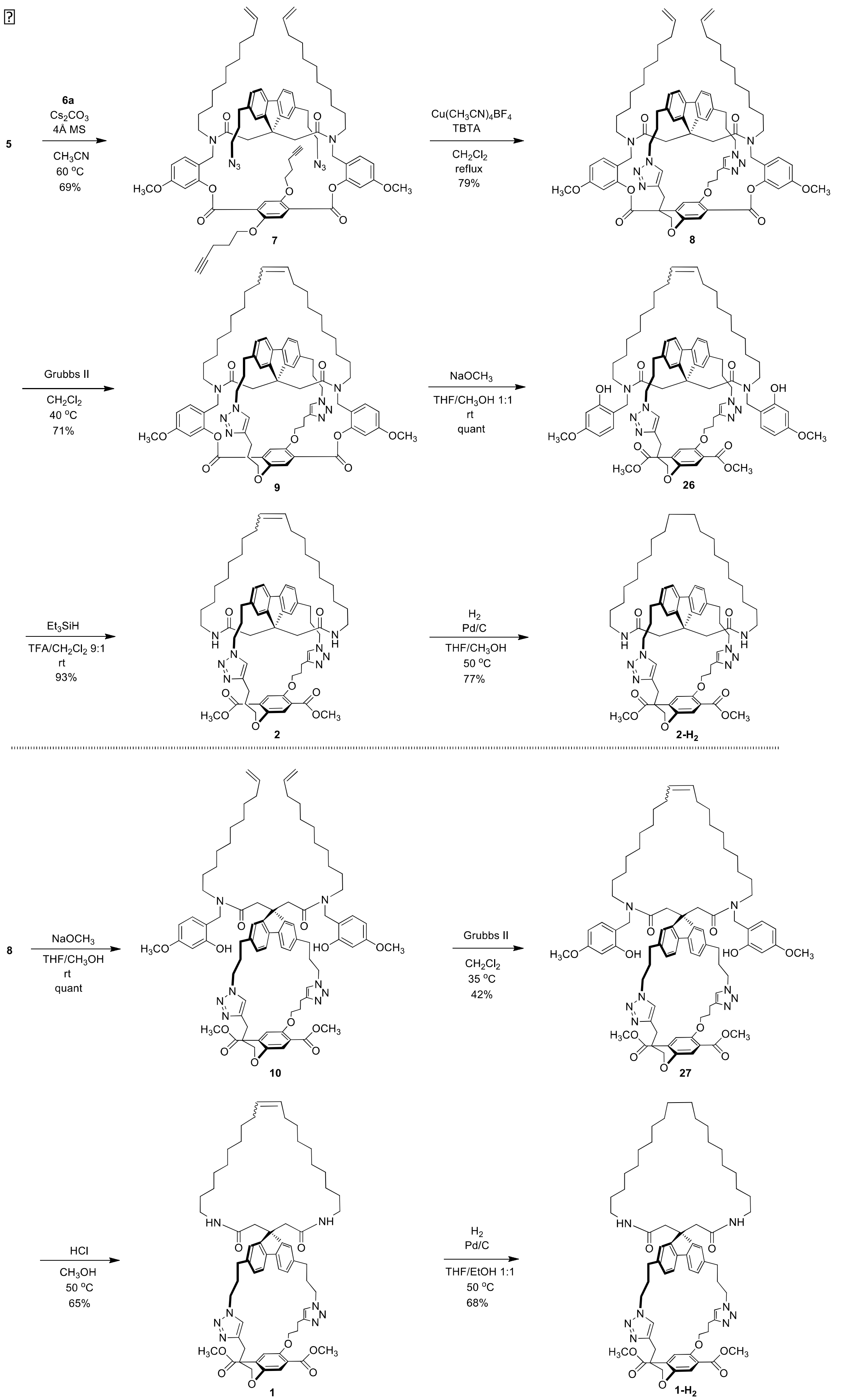

Supplementary figure 3. Synthesis of compounds 1 and 2

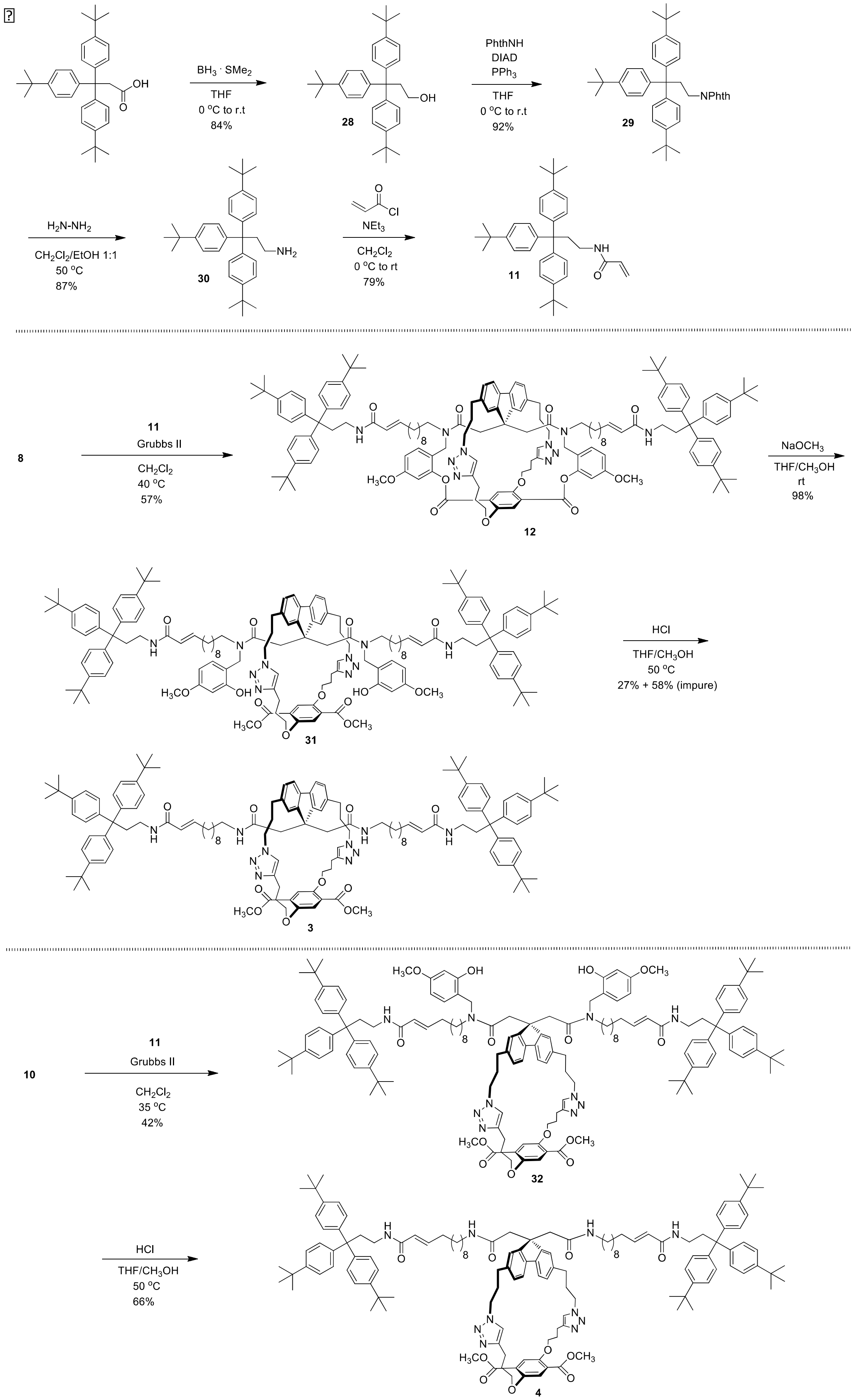

Supplementary figure 4. Synthesis of compounds 11, 3 and 4

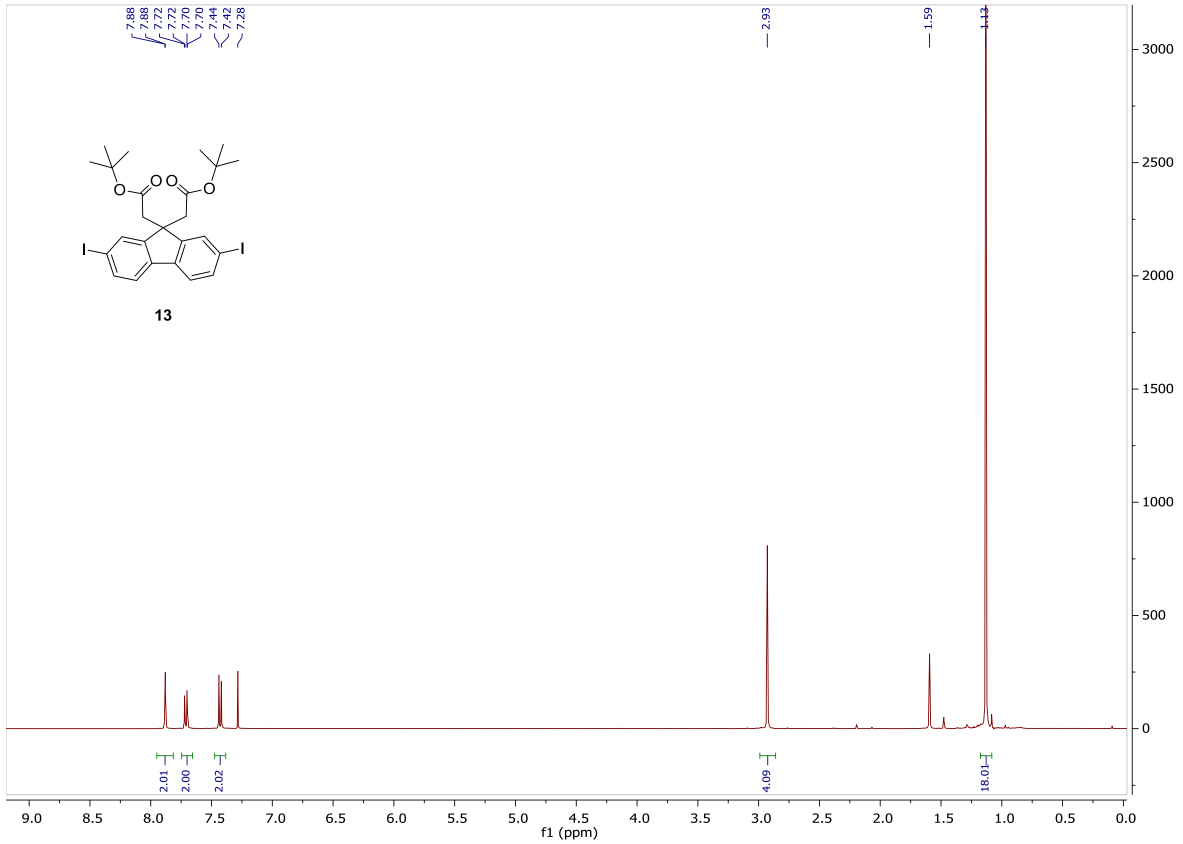

Supplementary figure 5. ^{1}H-NMR spectrum of compound 13 ($CDCl_3$, 300 MHz)

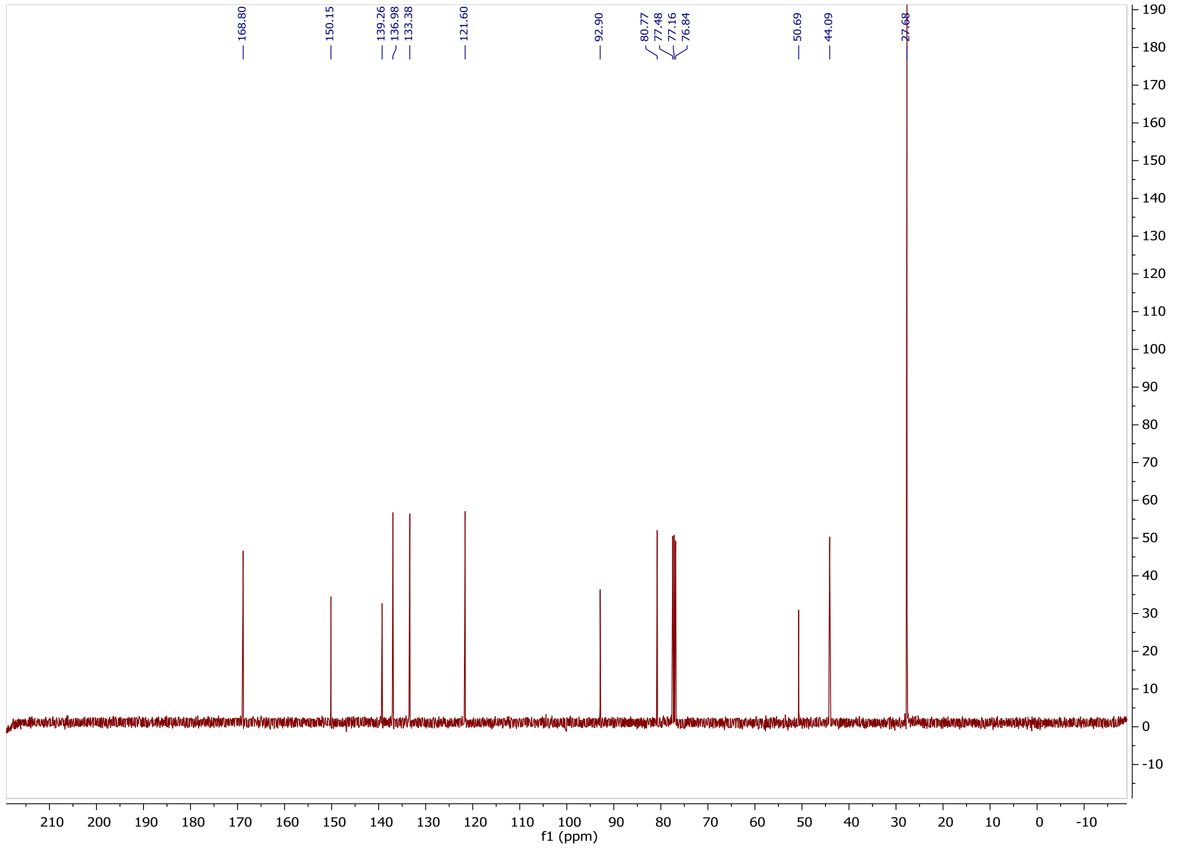

Supplementary figure 6. ^{13}C-NMR spectrum of compound 13 ($CDCl_3$, 75 MHz)

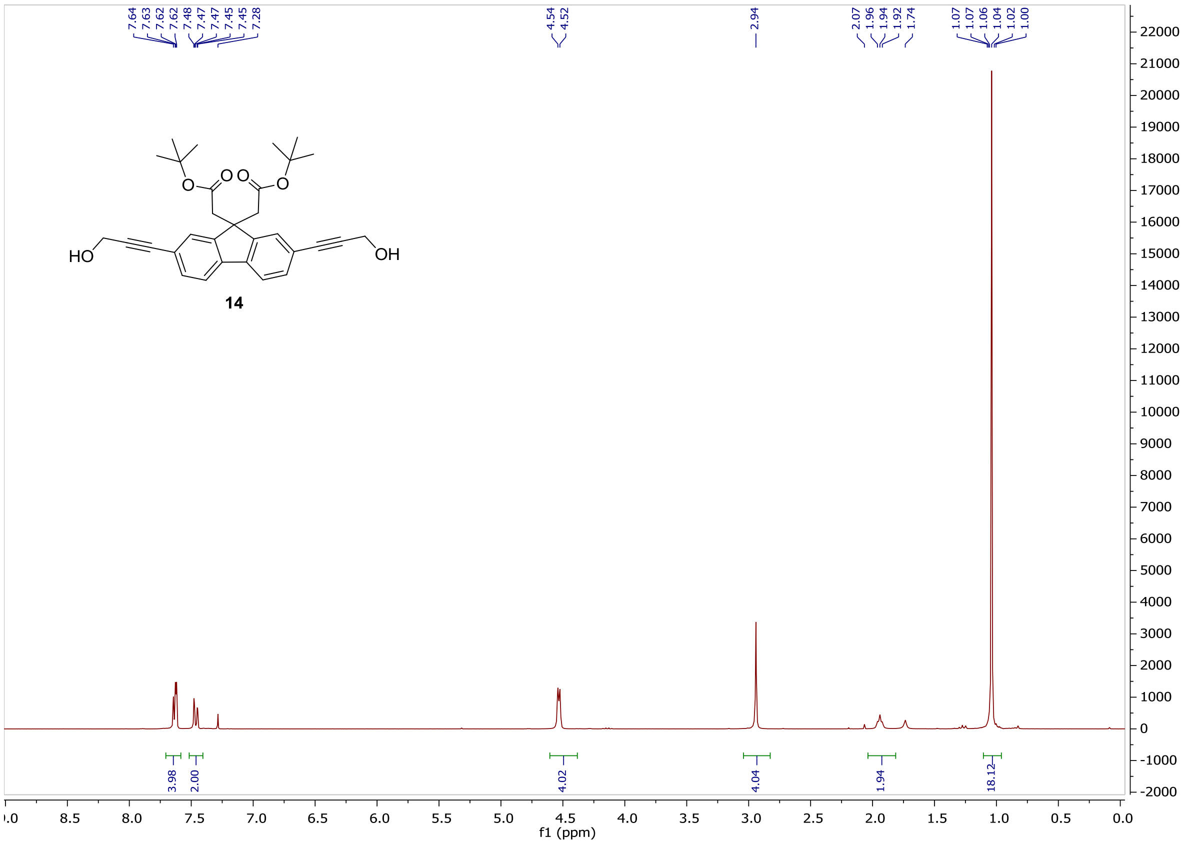

Supplementary figure 7. ¹H-NMR spectrum of compound 14 ($CDCl_3$, 300 MHz)

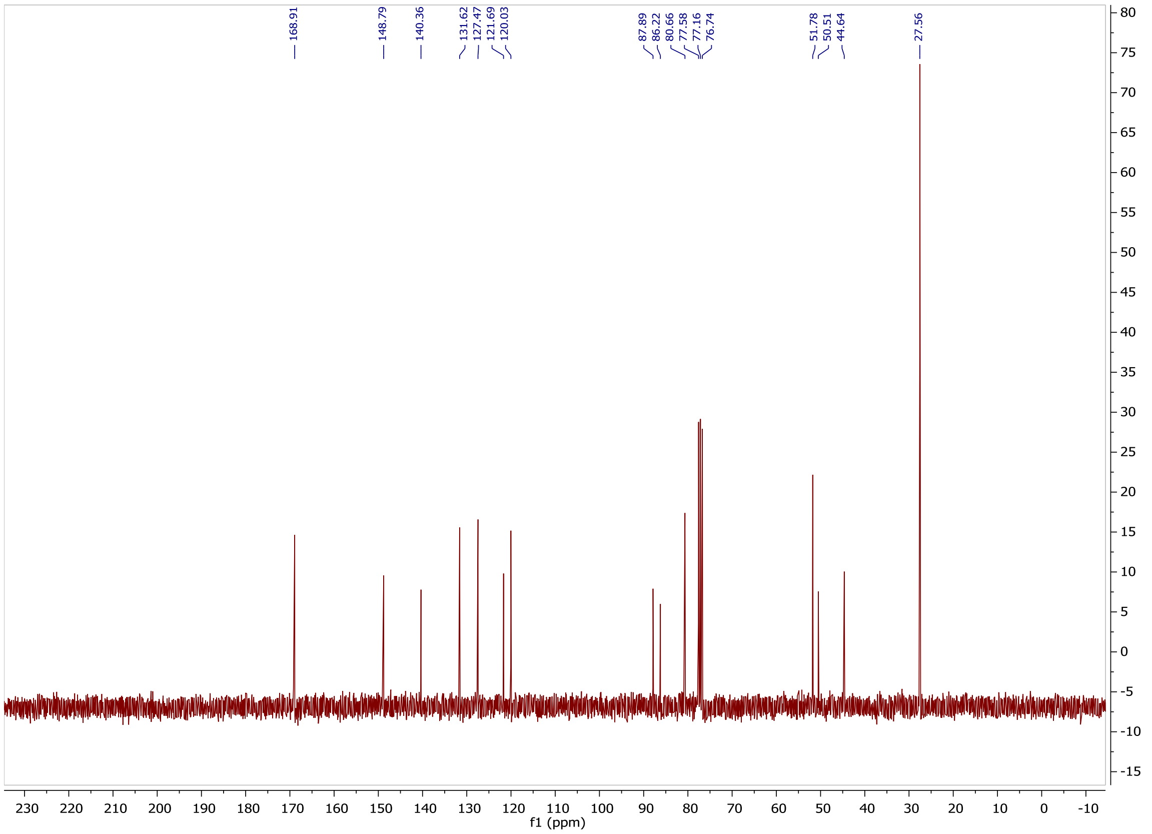

Supplementary figure 8. ¹³C-NMR spectrum of compound 14 ($CDCl_3$, 75 MHz)

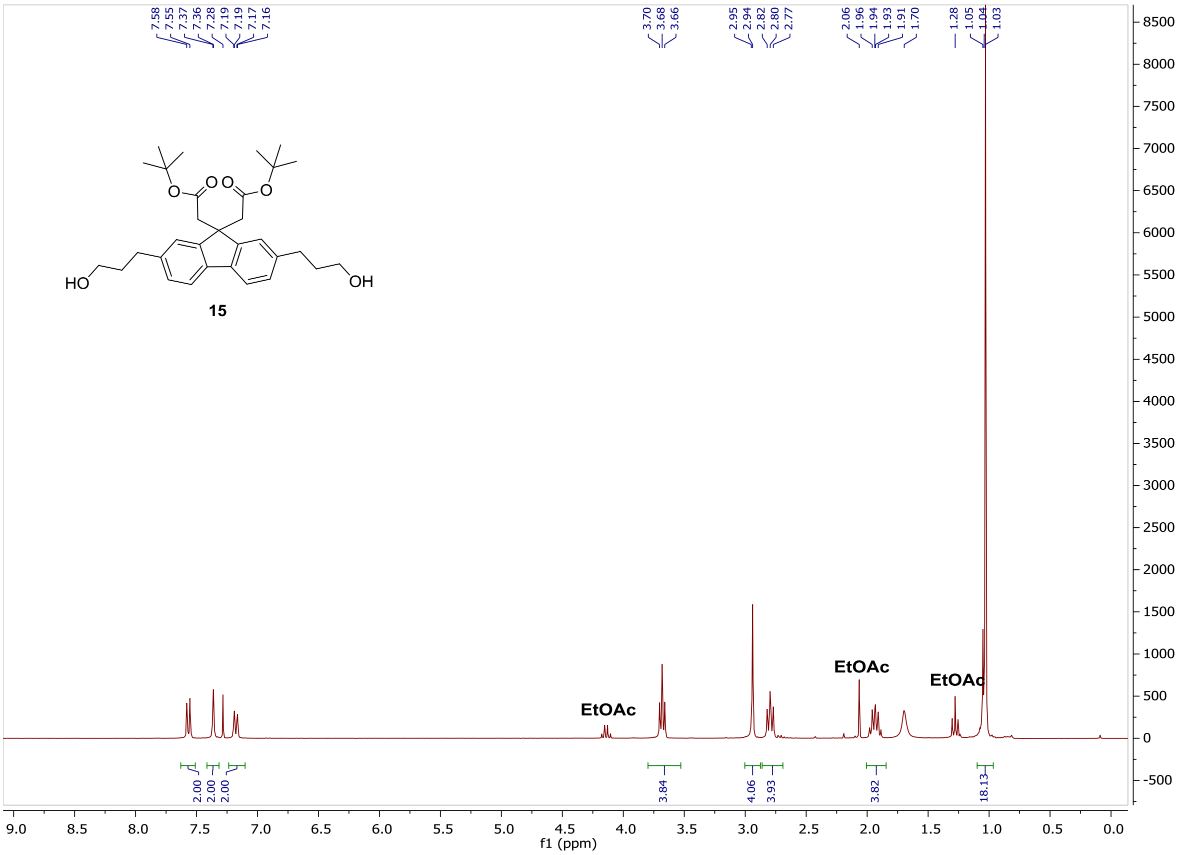

Supplementary figure 9. ^{1}**H-NMR spectrum of compound 15 ($CDCl_3$, 300 MHz)**

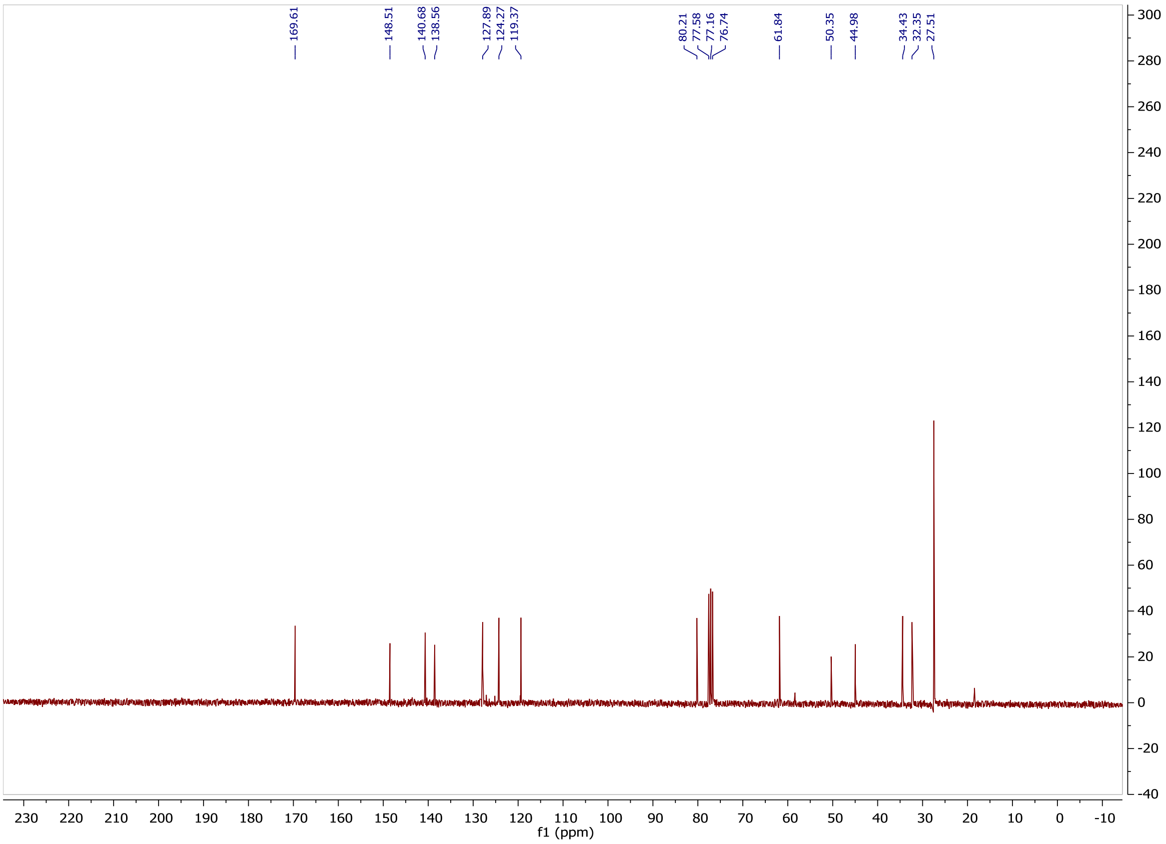

Supplementary figure 10. ^{13}**C-NMR spectrum of compound 15 ($CDCl_3$, 75 MHz)**

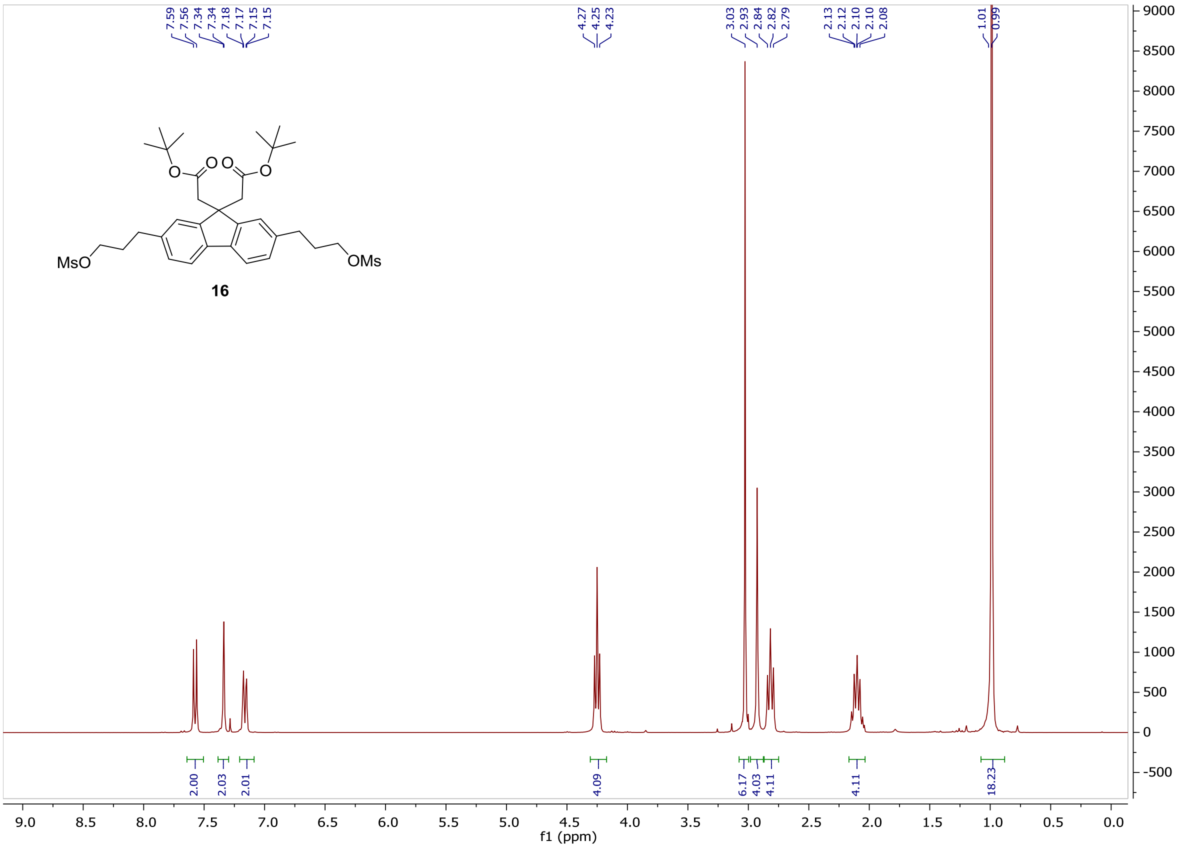

Supplementary figure 11. ^{1}H-NMR spectrum of compound 16 ($CDCl_3$, 300 MHz)

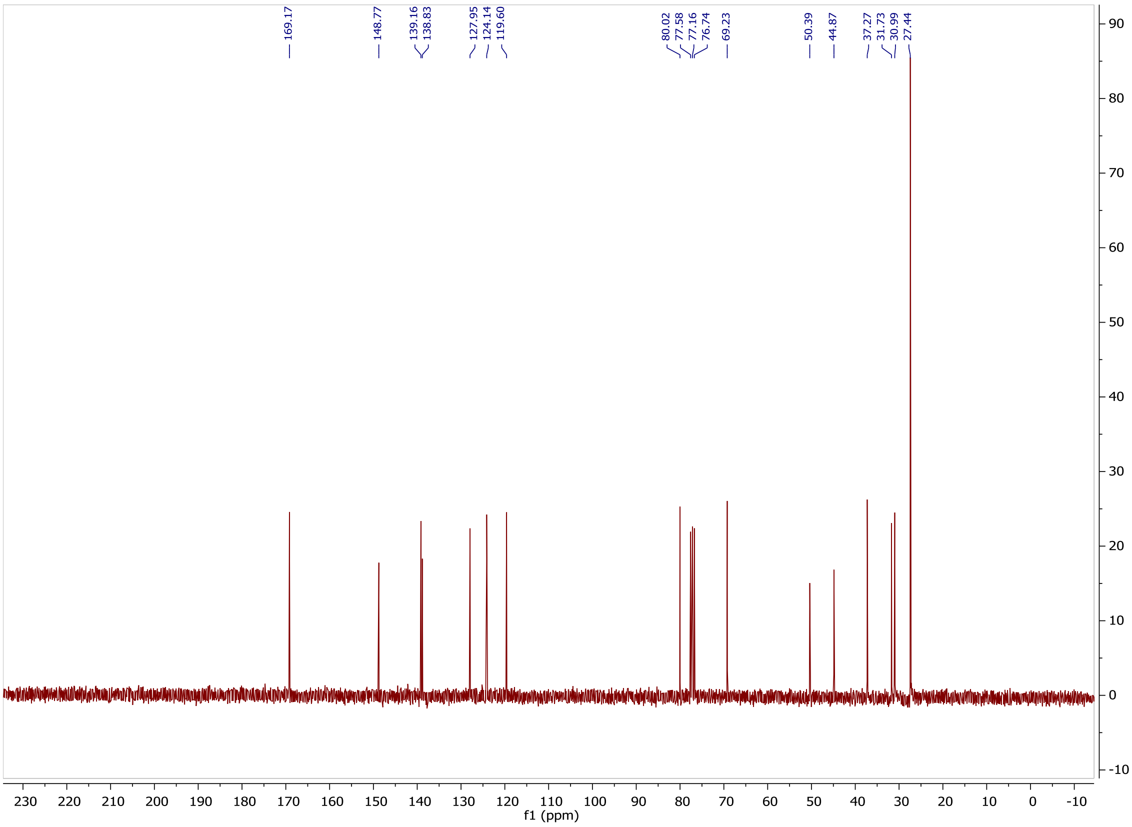

Supplementary figure 12. ^{13}C-NMR spectrum of compound 16 ($CDCl_3$, 75 MHz)

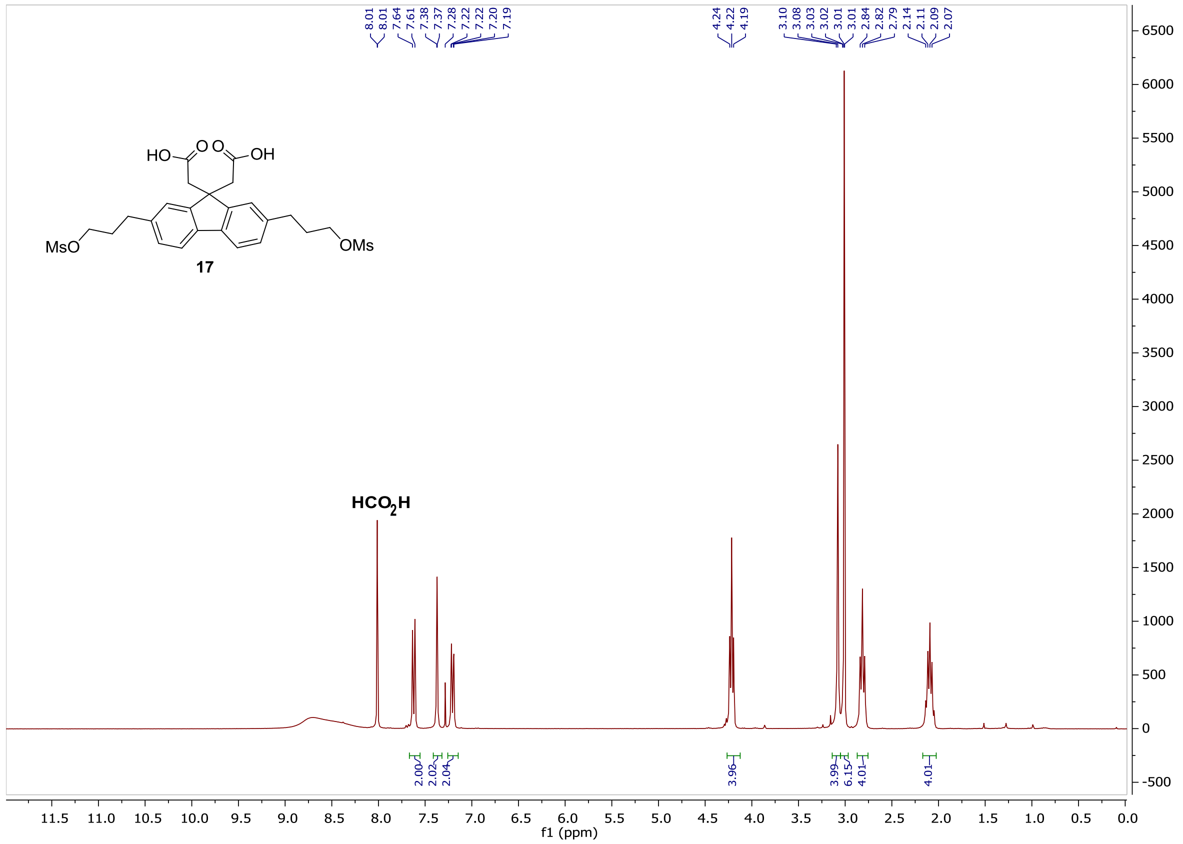

Supplementary figure 13. ^{1}H-NMR spectrum of compound 17 ($CDCl_3$, 300 MHz)

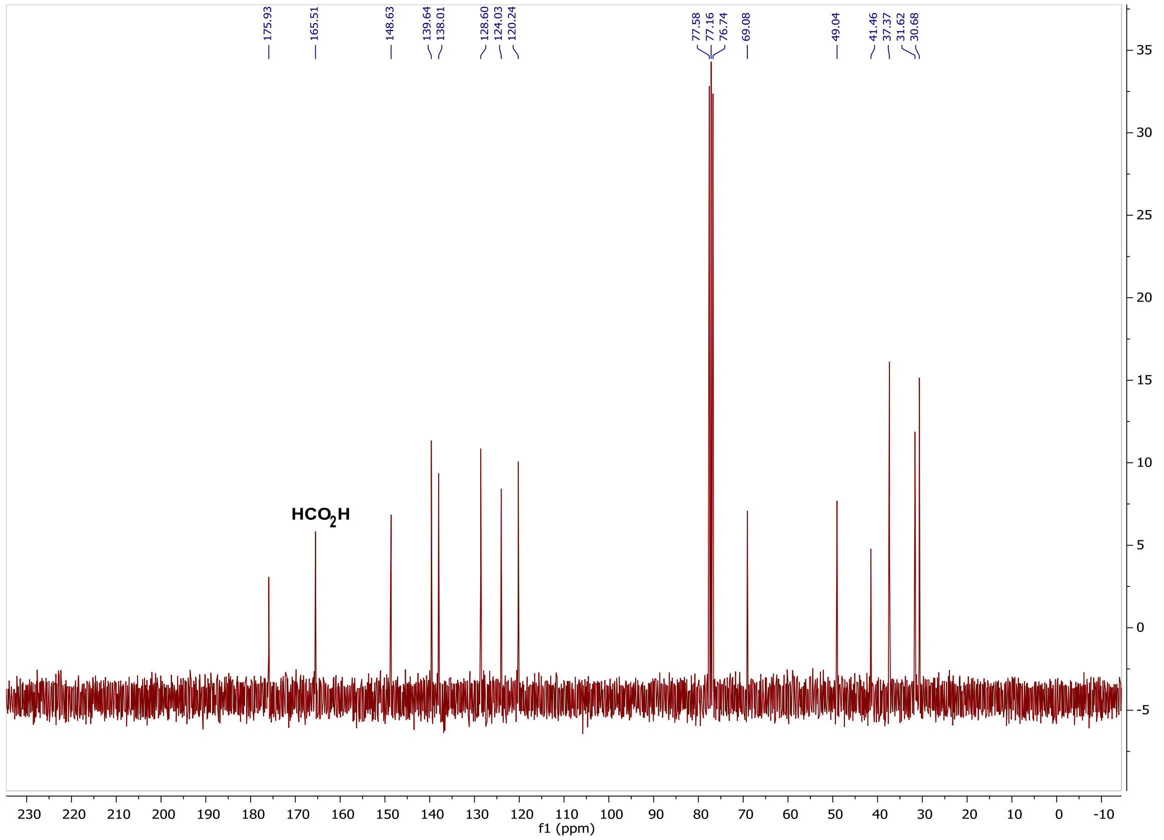

Supplementary figure 14. ^{13}C-NMR spectrum of compound 17 ($CDCl_3$, 75 MHz)

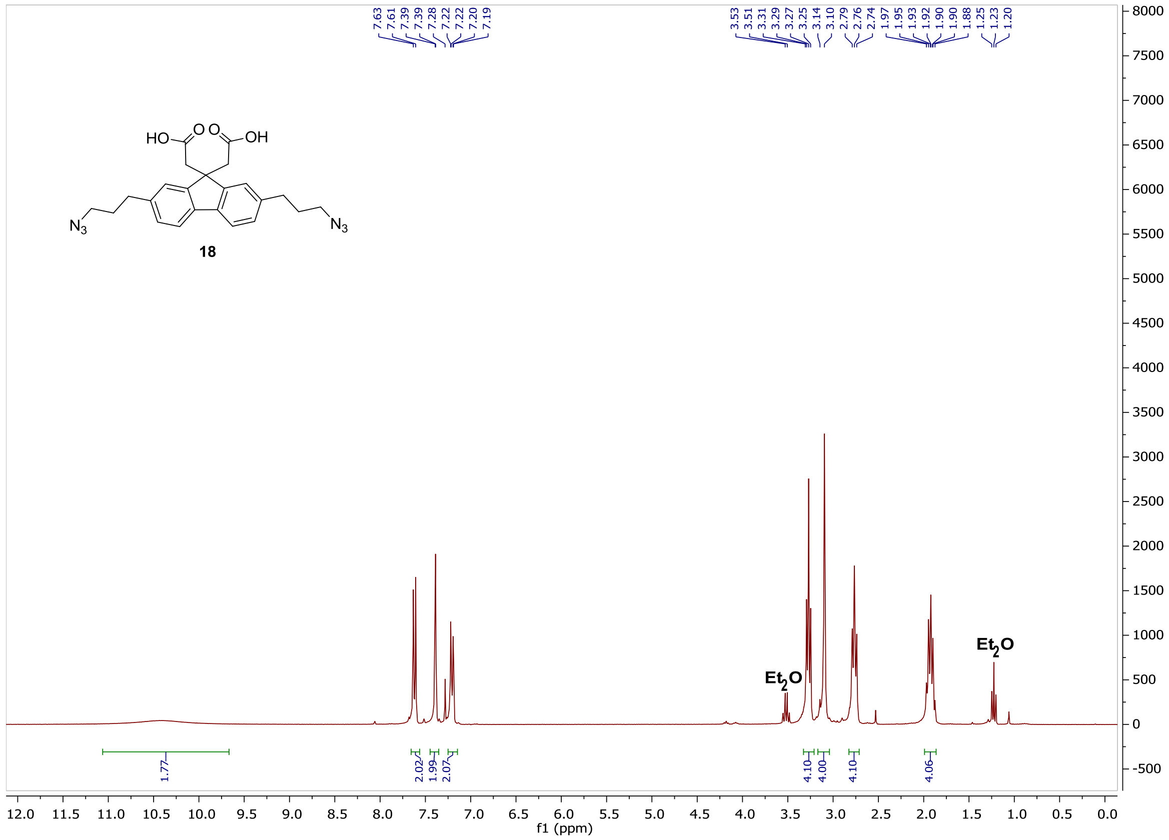

Supplementary figure 15. ^{1}H-NMR spectrum of compound 18 ($CDCl_3$, 300 MHz)

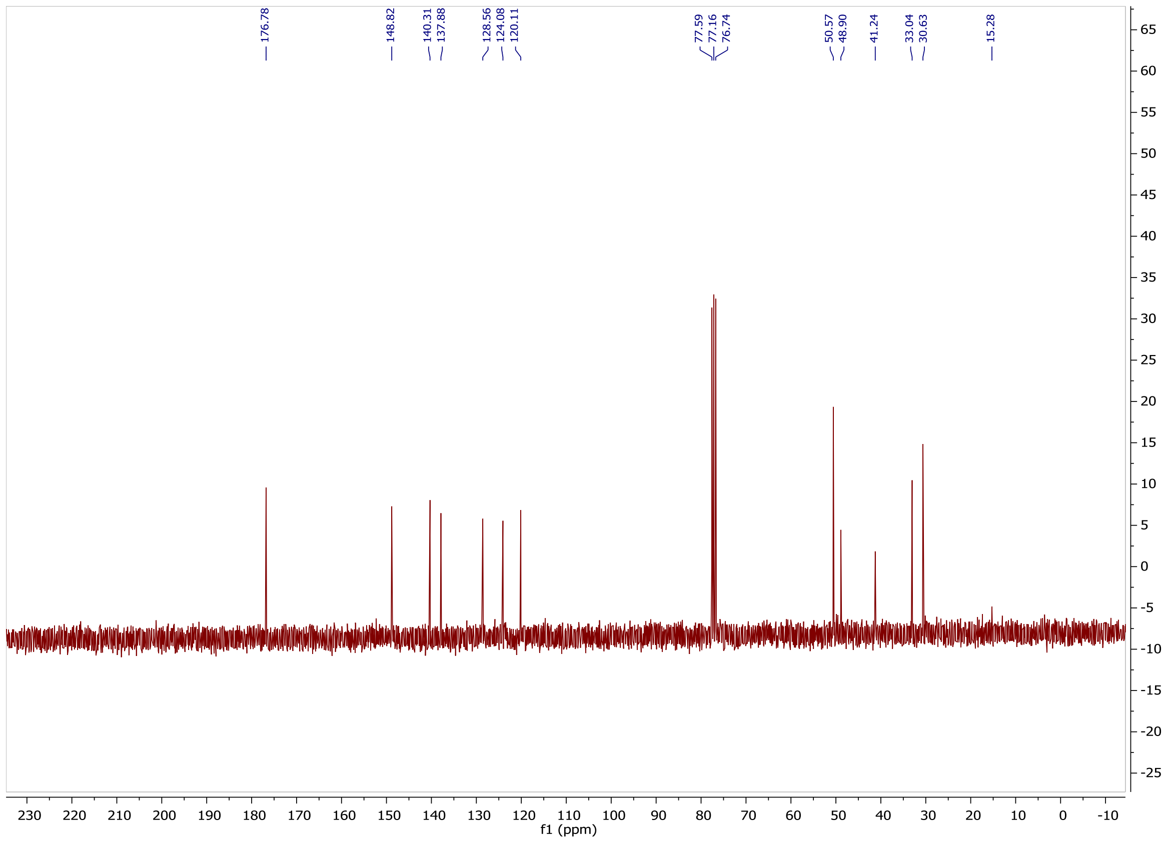

Supplementary figure 16. ^{13}C-NMR spectrum of compound 18 ($CDCl_3$, 75 MHz)

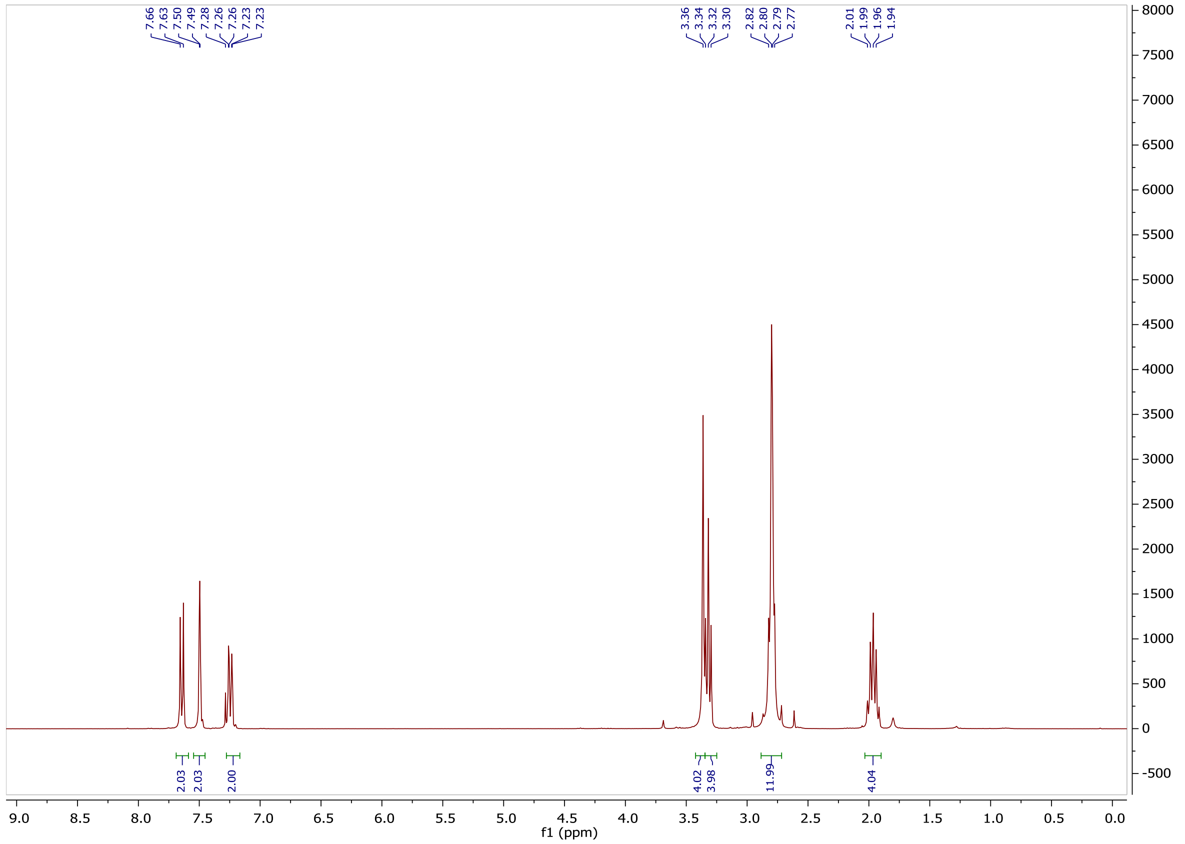

Supplementary figure 17. **^{1}H-NMR spectrum of compound 19 ($CDCl_3$, 300 MHz)**

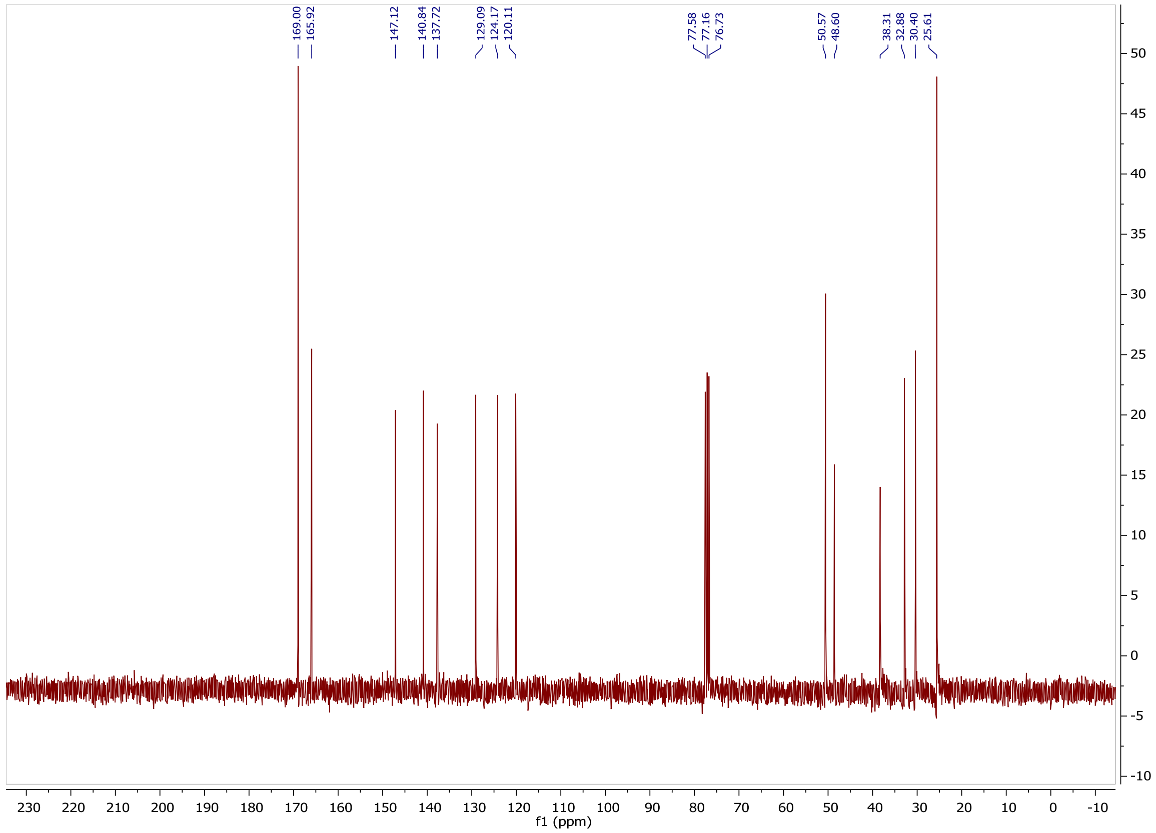

Supplementary figure 18. **^{13}C-NMR spectrum of compound 19 ($CDCl_3$, 75 MHz)**

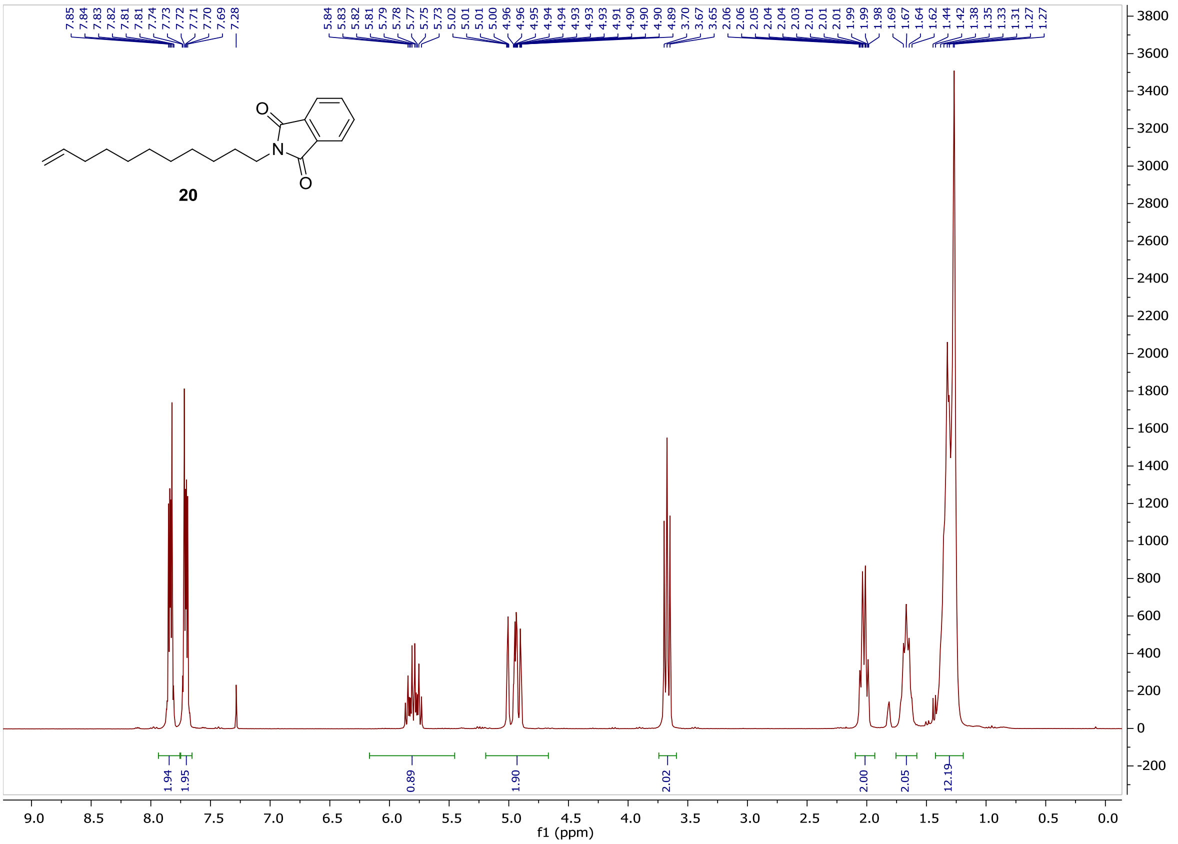

Supplementary figure 19. ^{1}**H-NMR spectrum of compound 20 (CDCl$_3$, 300 MHz)**

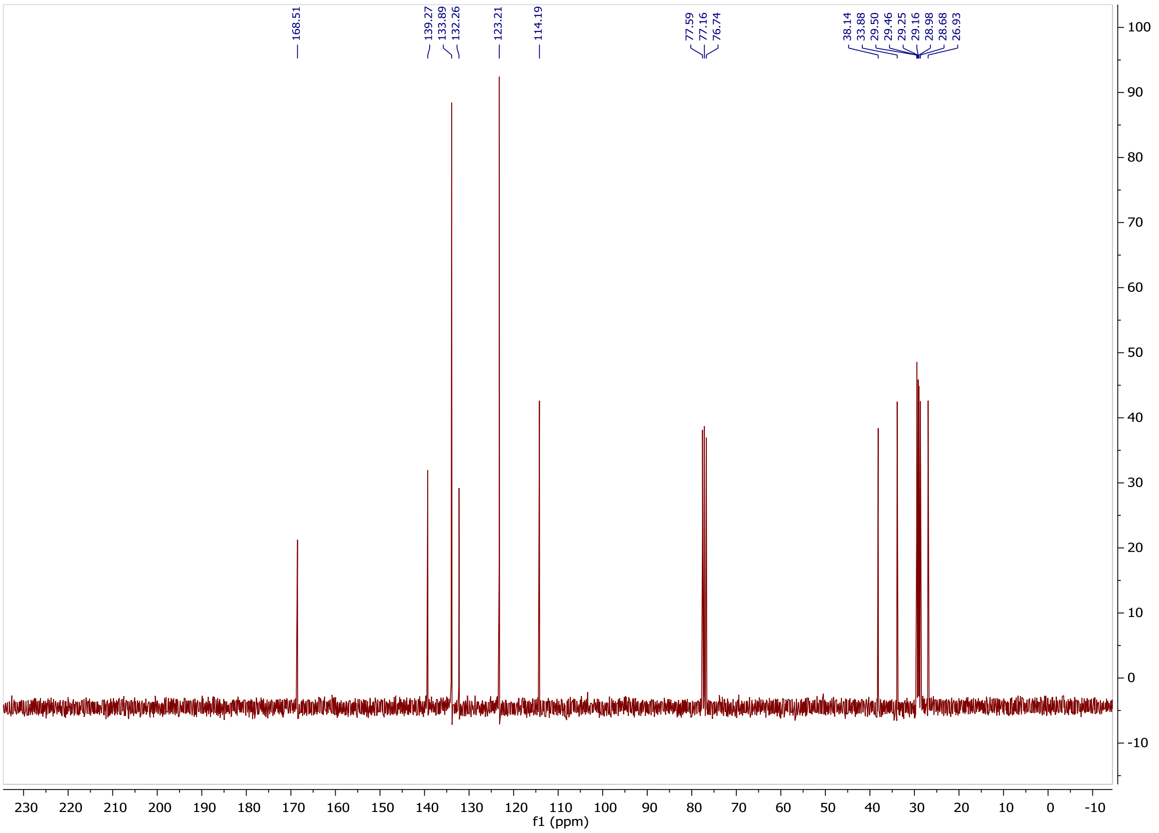

Supplementary figure 20. ^{13}**C-NMR spectrum of compound 20 (CDCl$_3$, 75 MHz)**

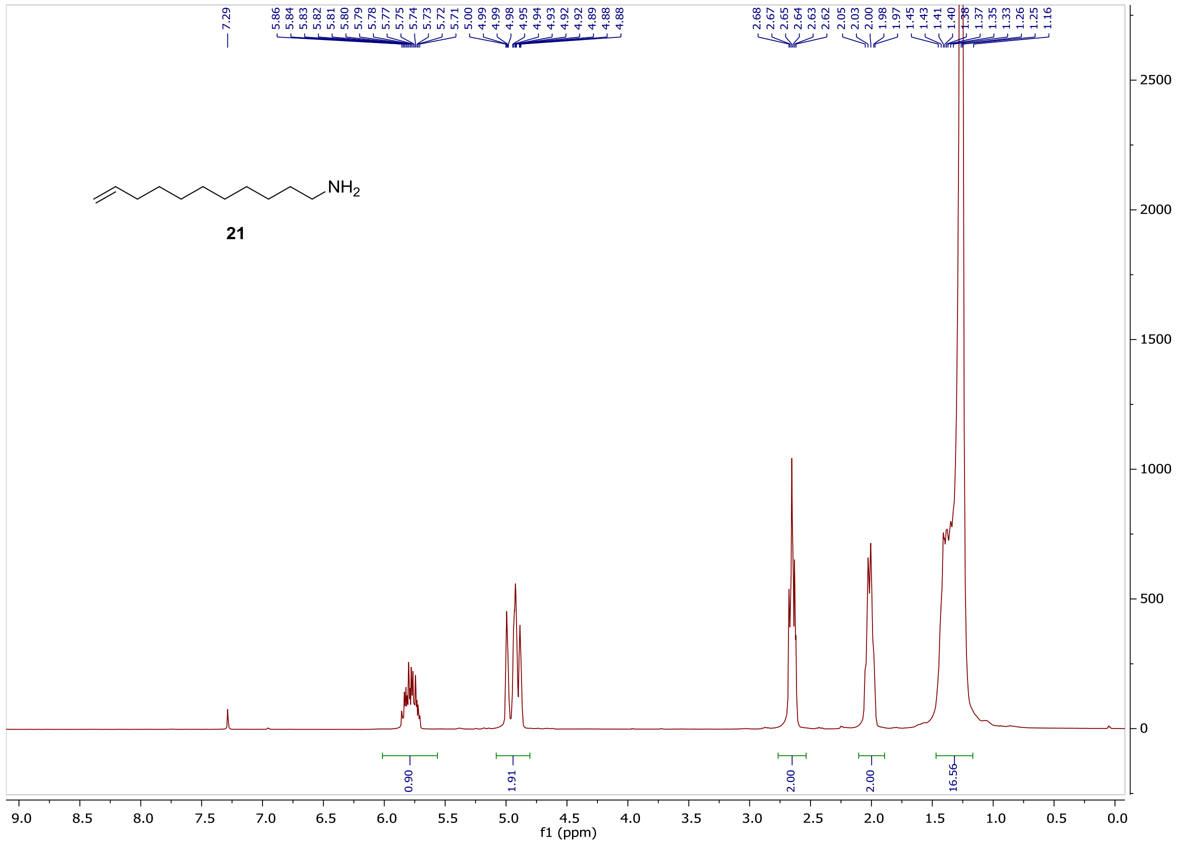

Supplementary figure 21. ^{1}H-NMR spectrum of compound 21 ($CDCl_3$, 300 MHz)

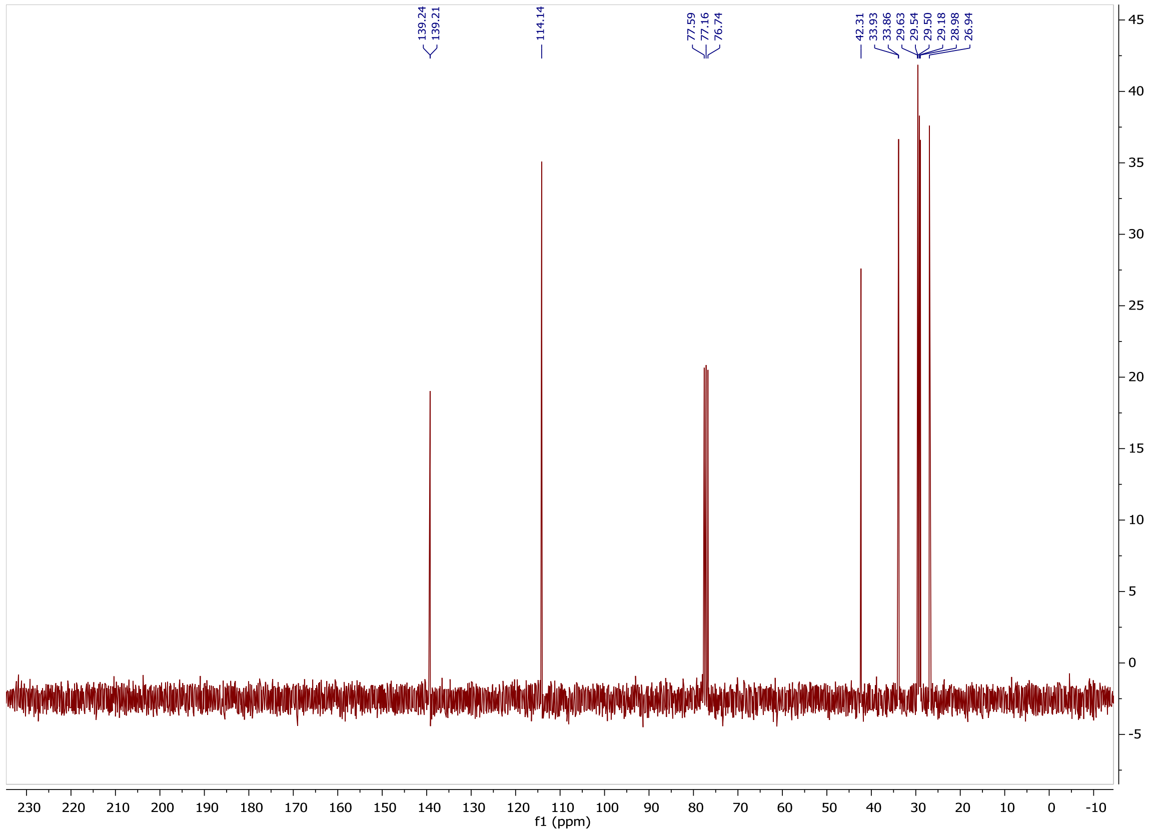

Supplementary figure 22. ^{13}C-NMR spectrum of compound 21 ($CDCl_3$, 75 MHz)

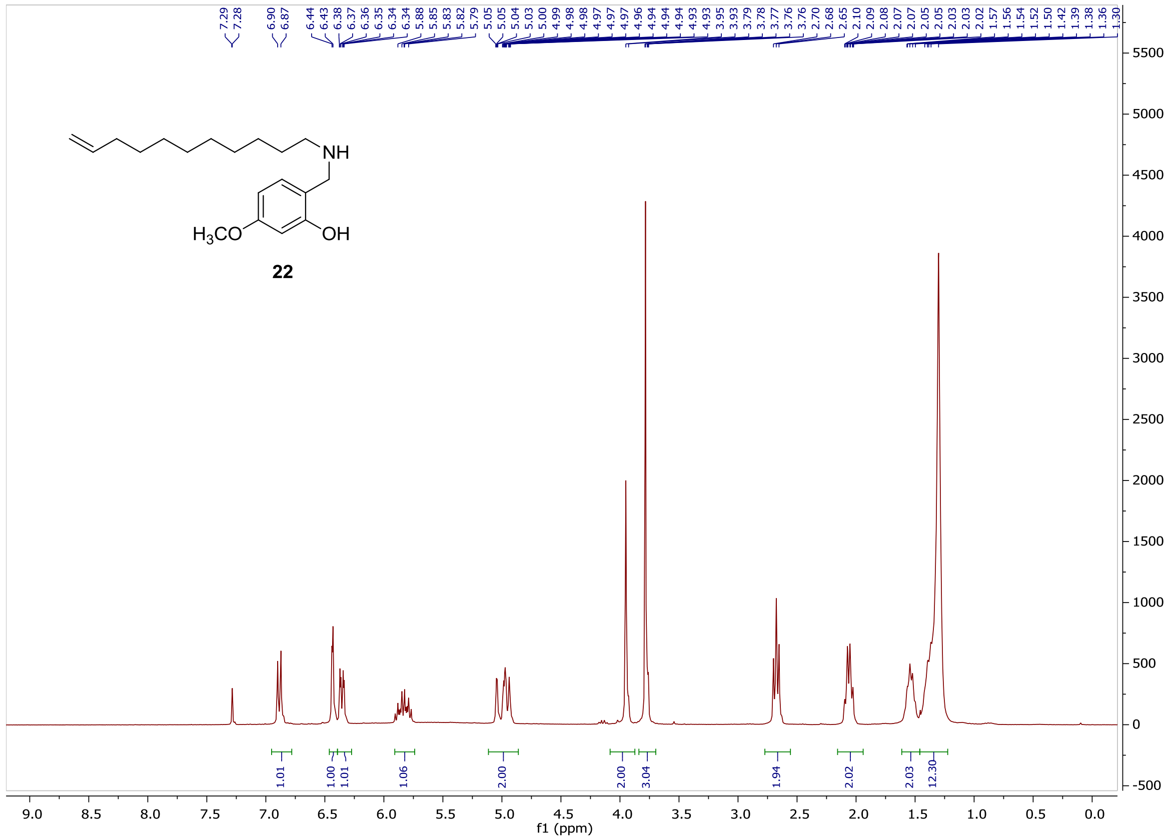

Supplementary figure 23. ^{1}H-NMR spectrum of compound 22 ($CDCl_3$, 300 MHz)

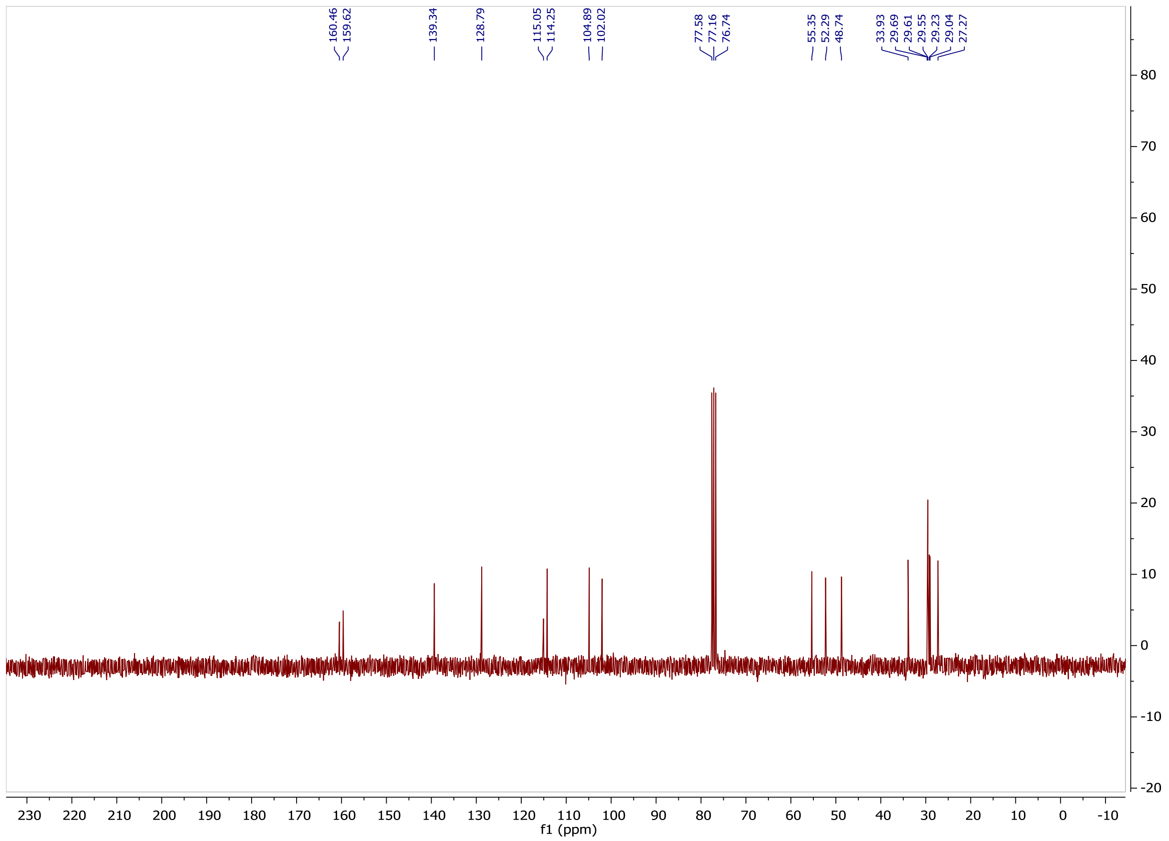

Supplementary figure 24. ^{13}C-NMR spectrum of compound 22 ($CDCl_3$, 75 MHz)

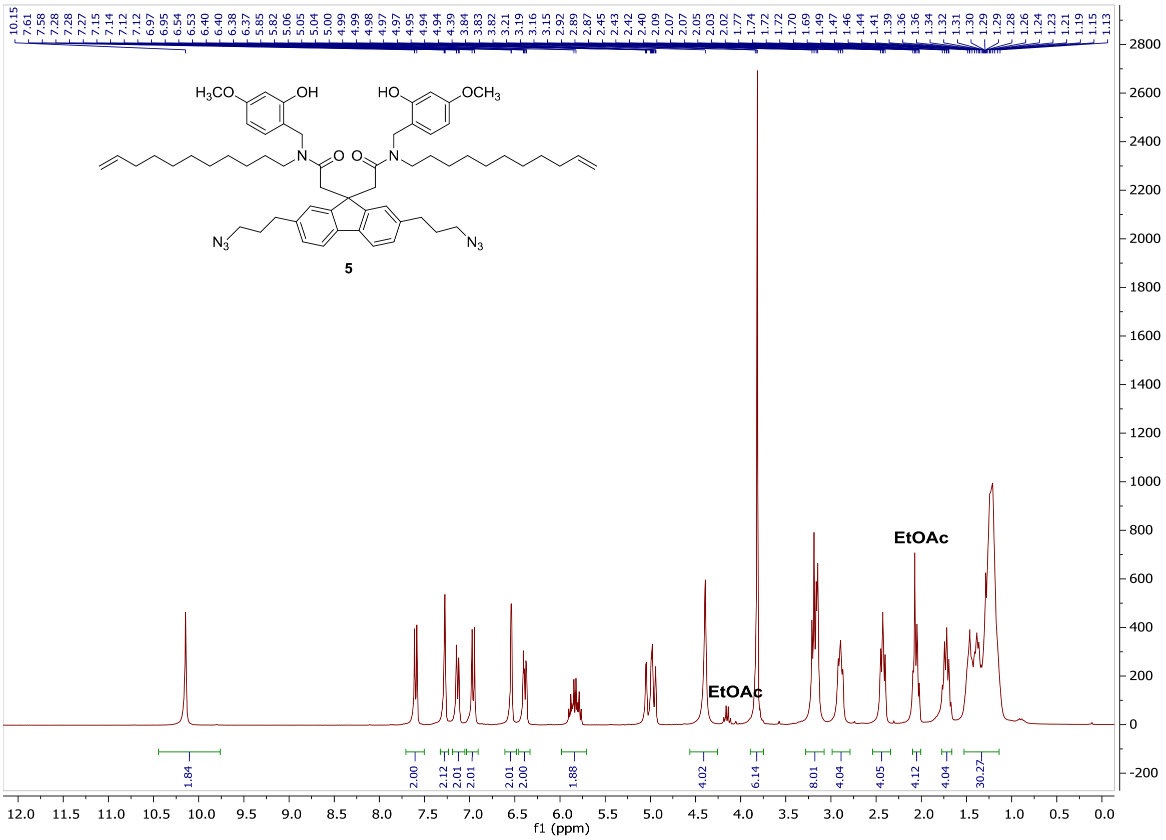

Supplementary figure 25. ^{1}H-NMR spectrum of compound 5 ($CDCl_3$, 300 MHz)

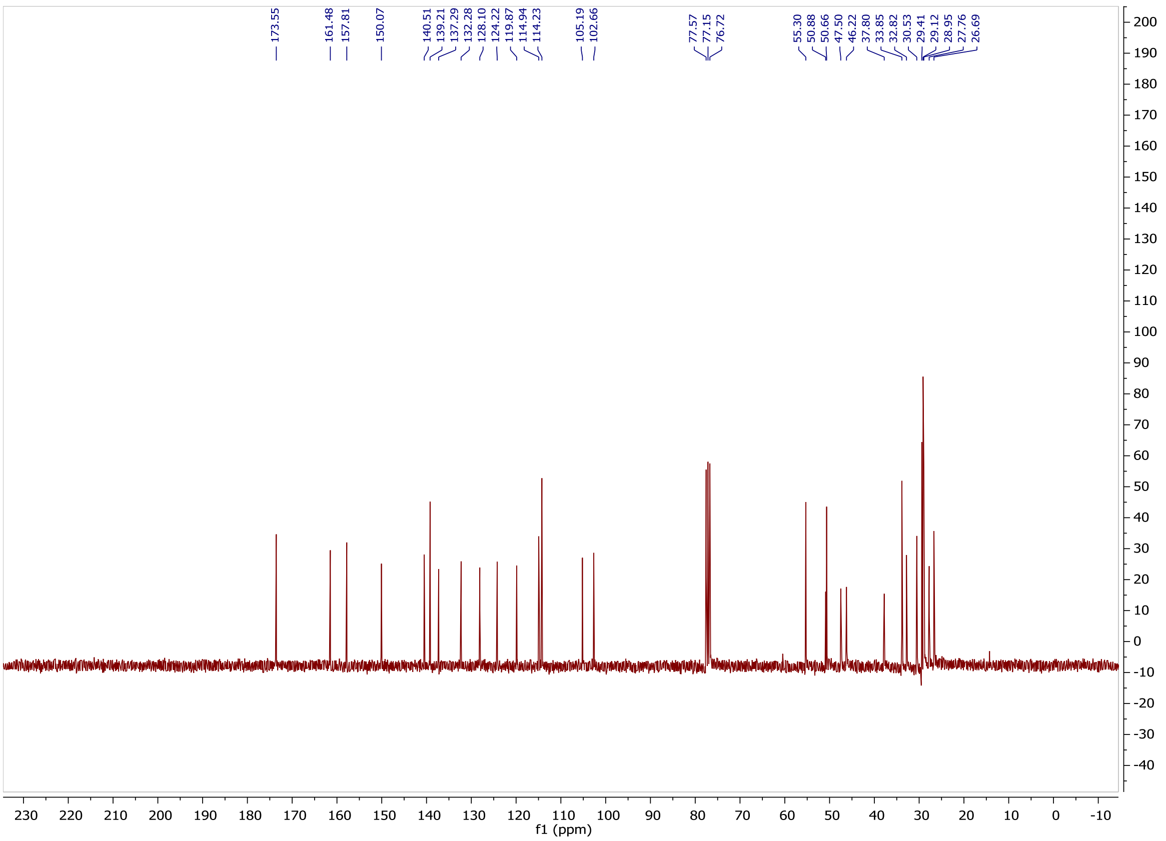

Supplementary figure 26. ^{13}C-NMR spectrum of compound 5 ($CDCl_3$, 75 MHz)

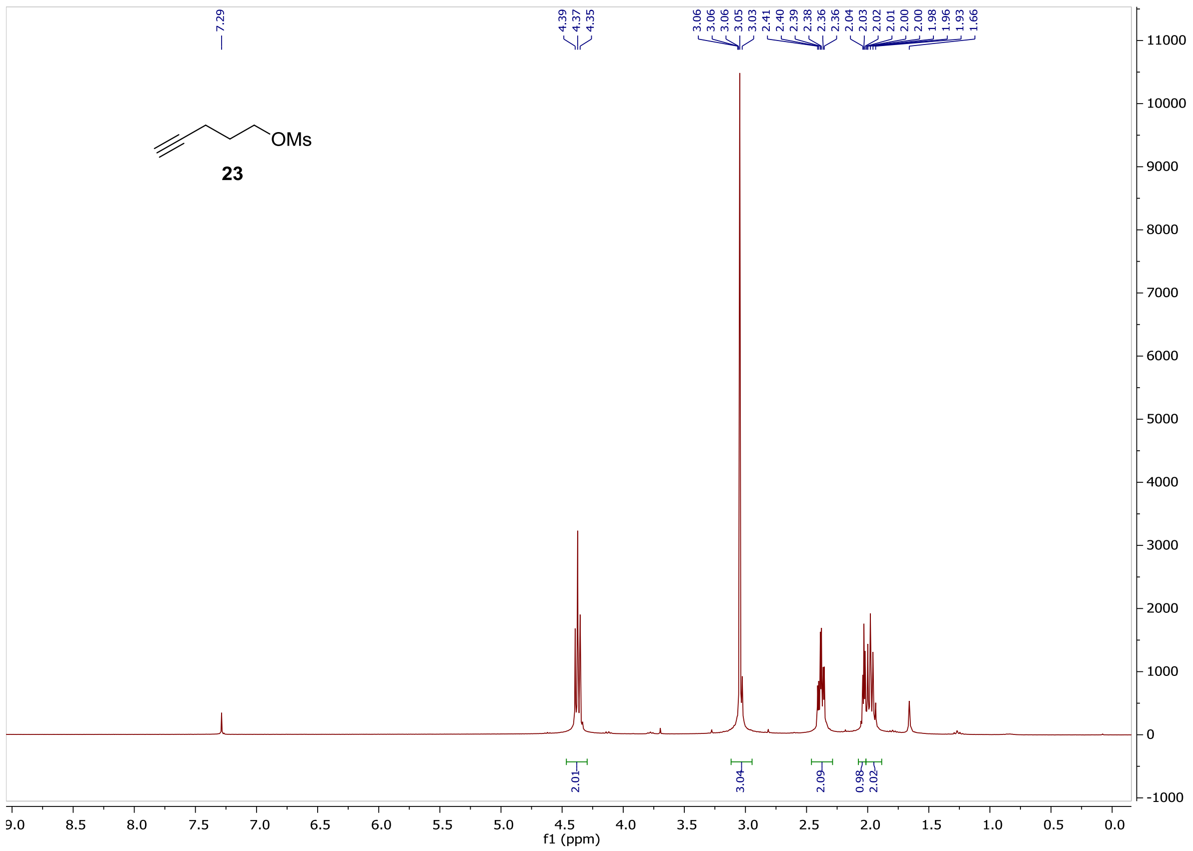

Supplementary figure 27. ^{1}H-NMR spectrum of compound 23 ($CDCl_3$, 300 MHz)

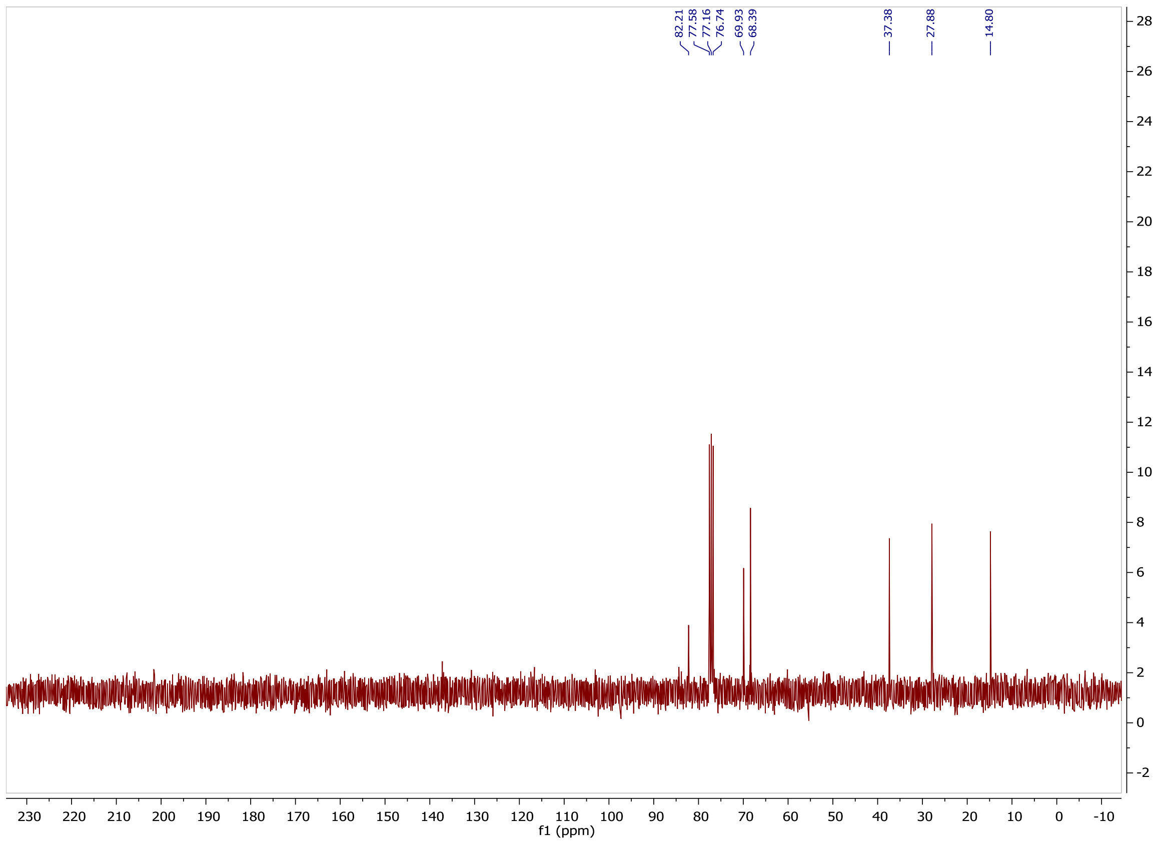

Supplementary figure 28. ^{13}C-NMR spectrum of compound 23 ($CDCl_3$, 75 MHz)

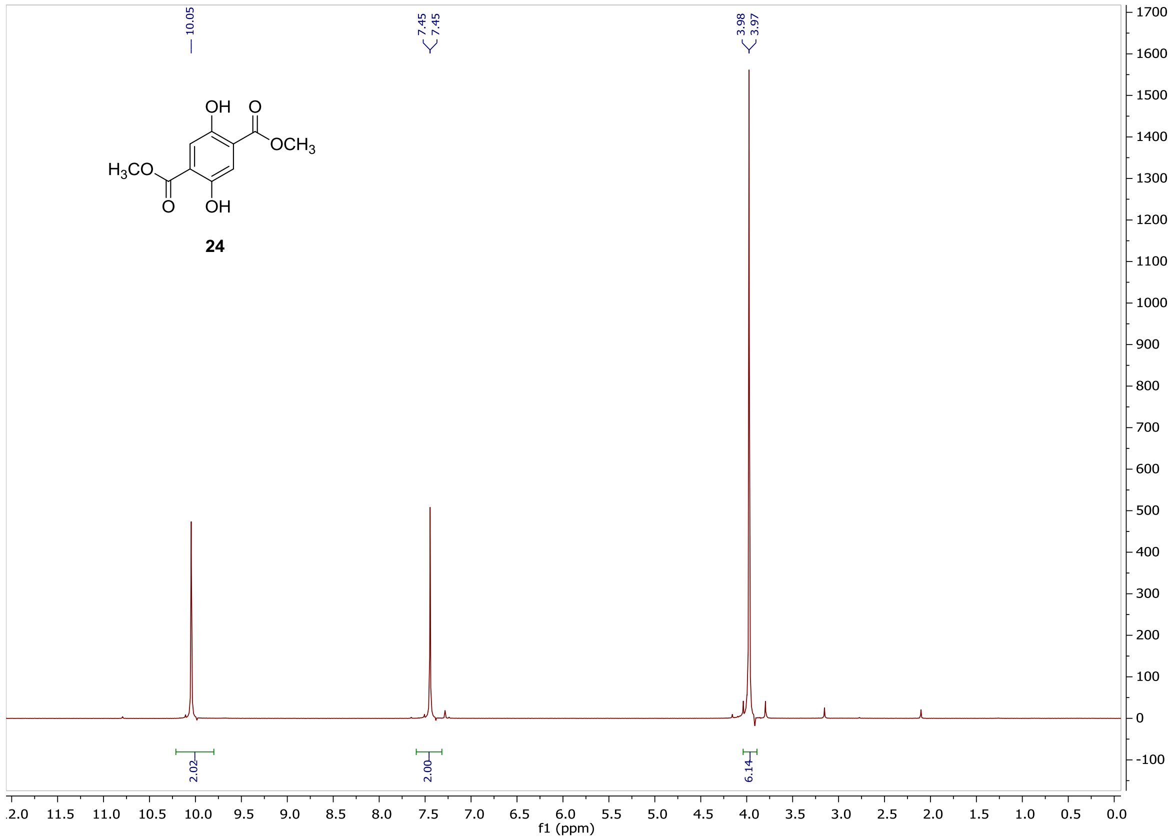

Supplementary figure 29. ^{1}**H-NMR spectrum of compound 24 (CDCl$_3$, 300 MHz)**

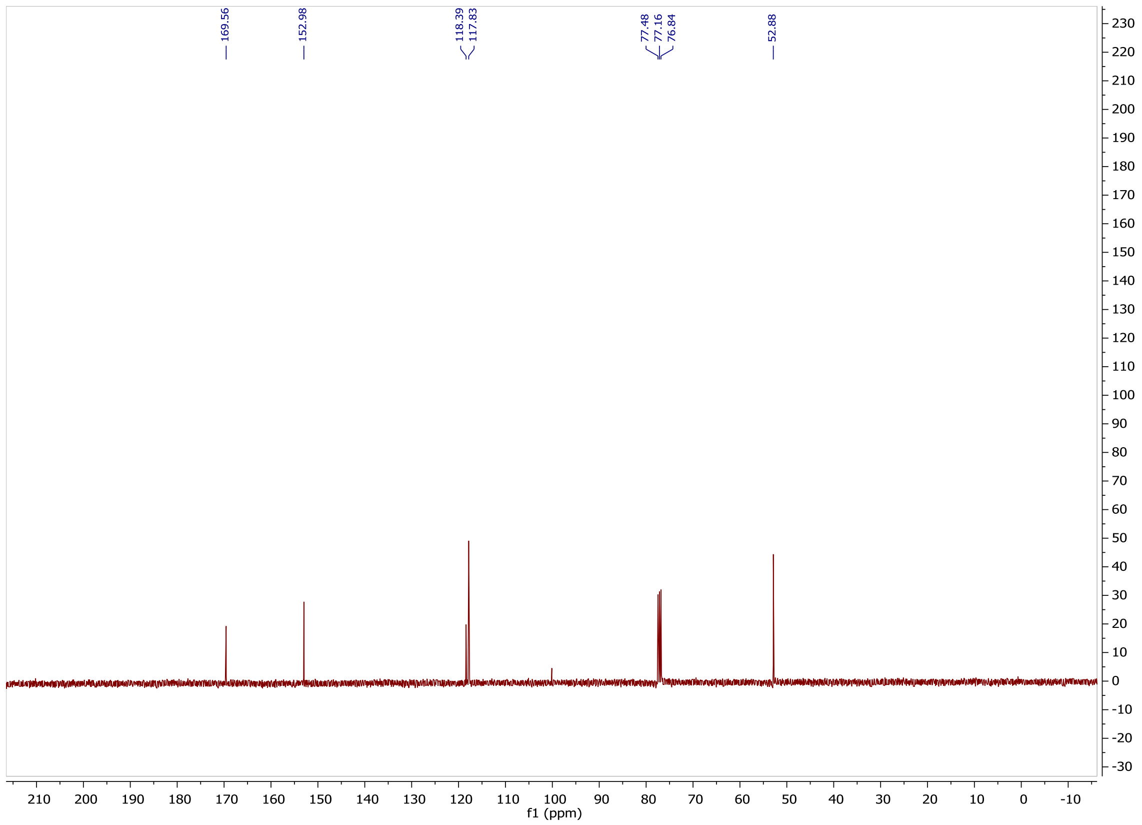

Supplementary figure 30. ^{13}**C-NMR spectrum of compound 24 (CDCl$_3$, 75 MHz)**

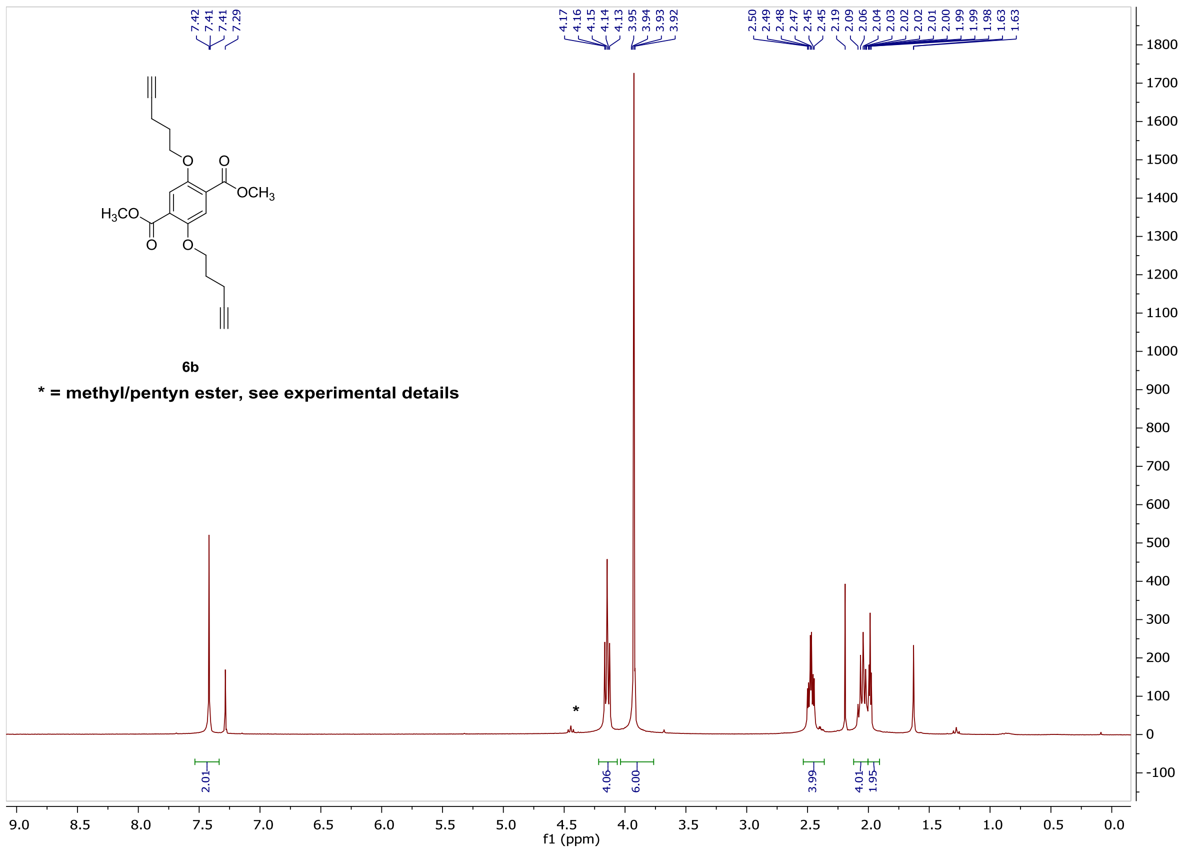

Supplementary figure 31. ^{1}H-NMR spectrum of compound 6b ($CDCl_3$, 300 MHz)

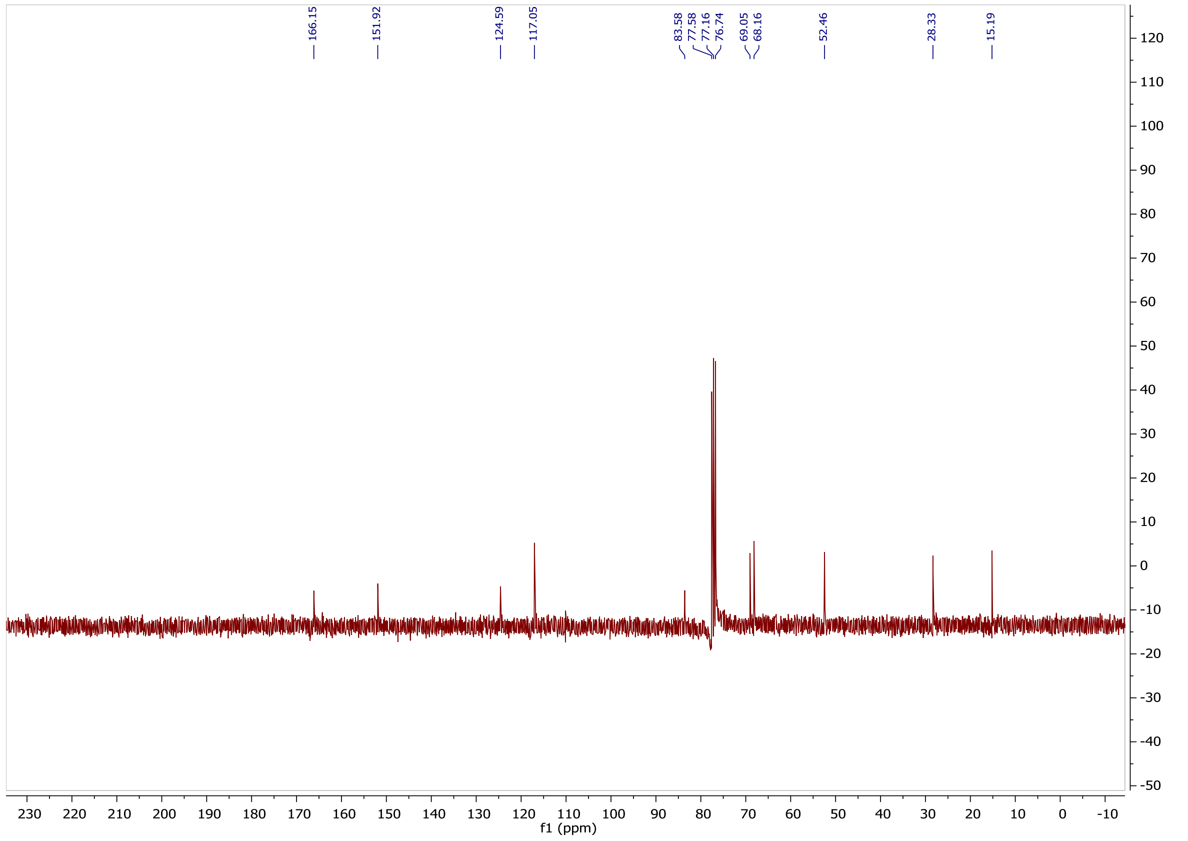

Supplementary figure 32. ^{13}C-NMR spectrum of compound 6b ($CDCl_3$, 75 MHz)

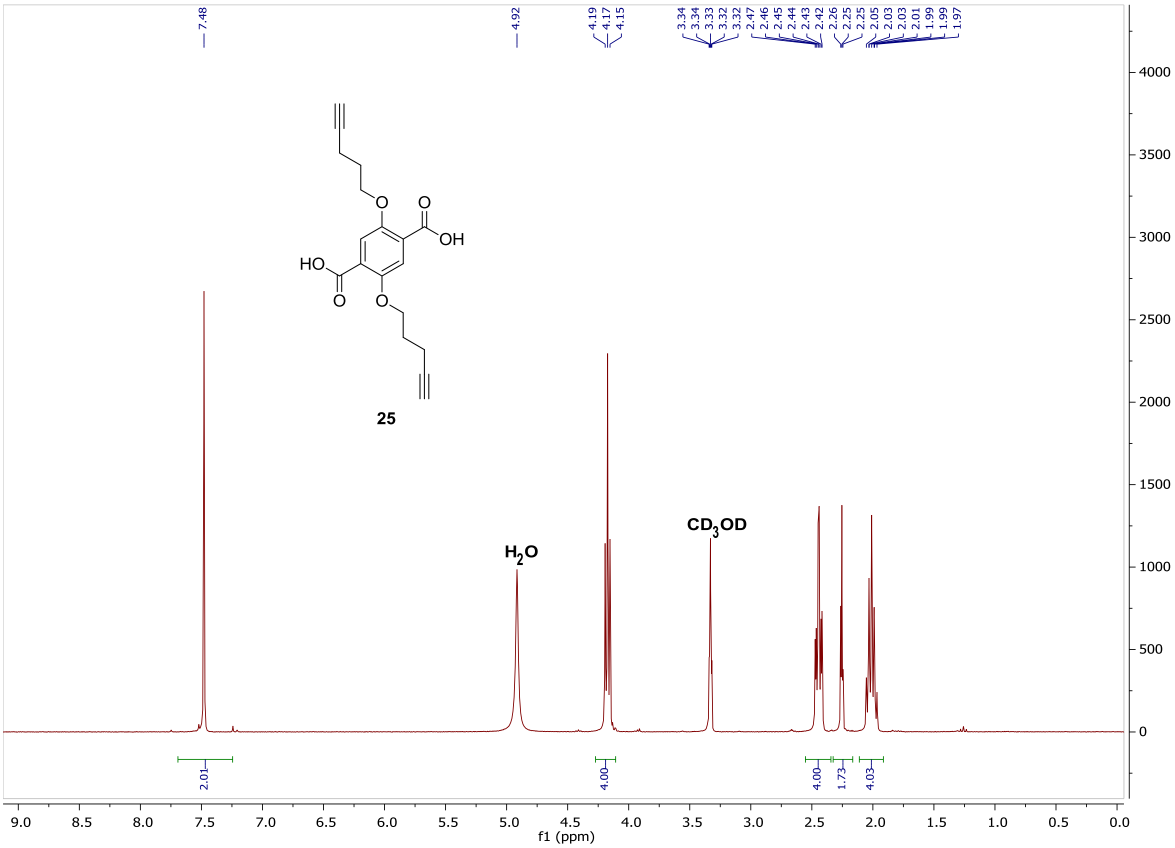

Supplementary figure 33. ^{1}**H-NMR spectrum of compound 25 ($CDCl_3$, 300 MHz)**

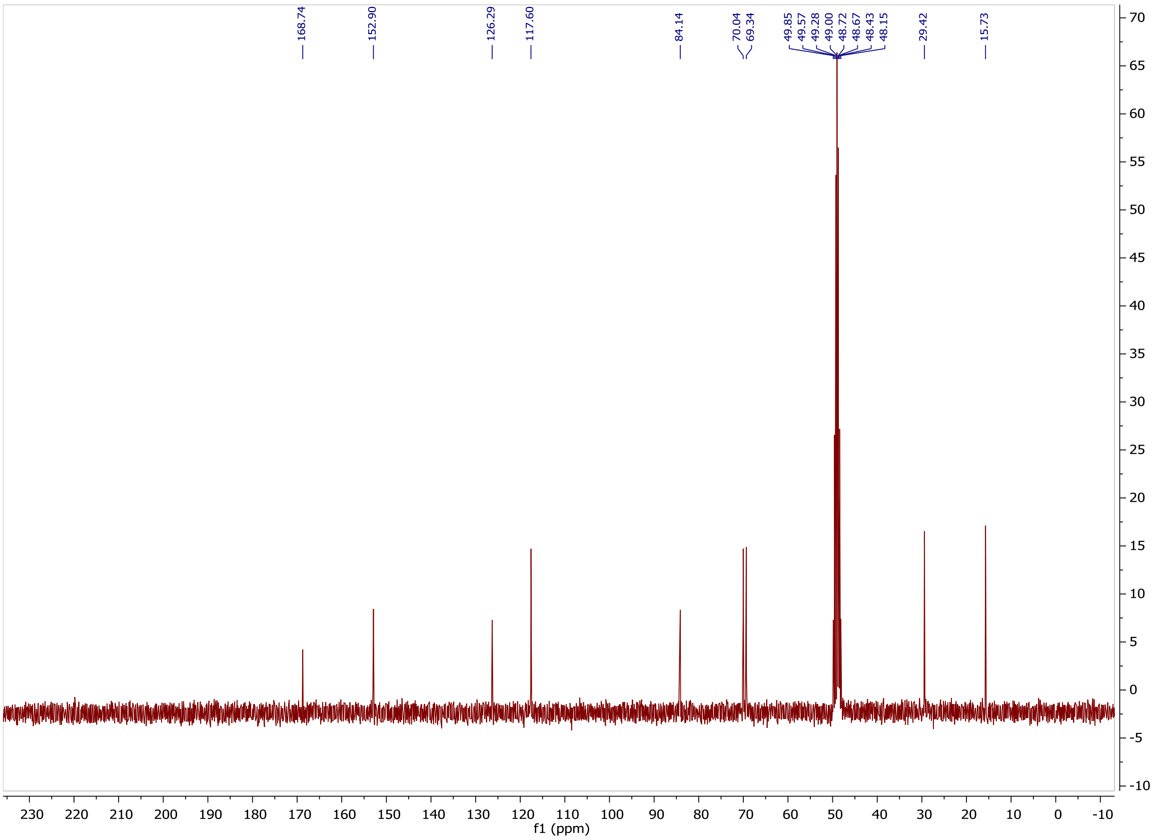

Supplementary figure 34. ^{13}**C-NMR spectrum of compound 25 ($CDCl_3$, 75 MHz)**

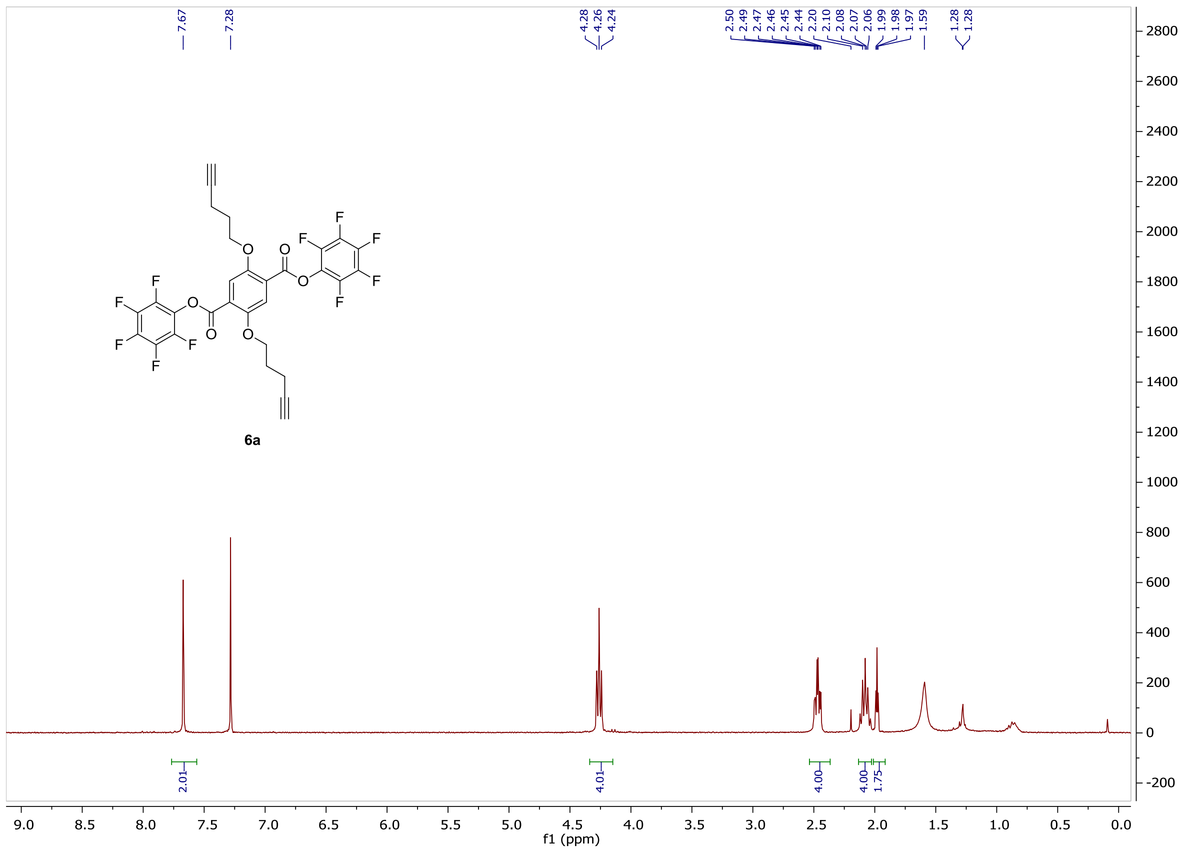

Supplementary figure 35. ^{1}H-NMR spectrum of compound 6a ($CDCl_3$, 300 MHz)

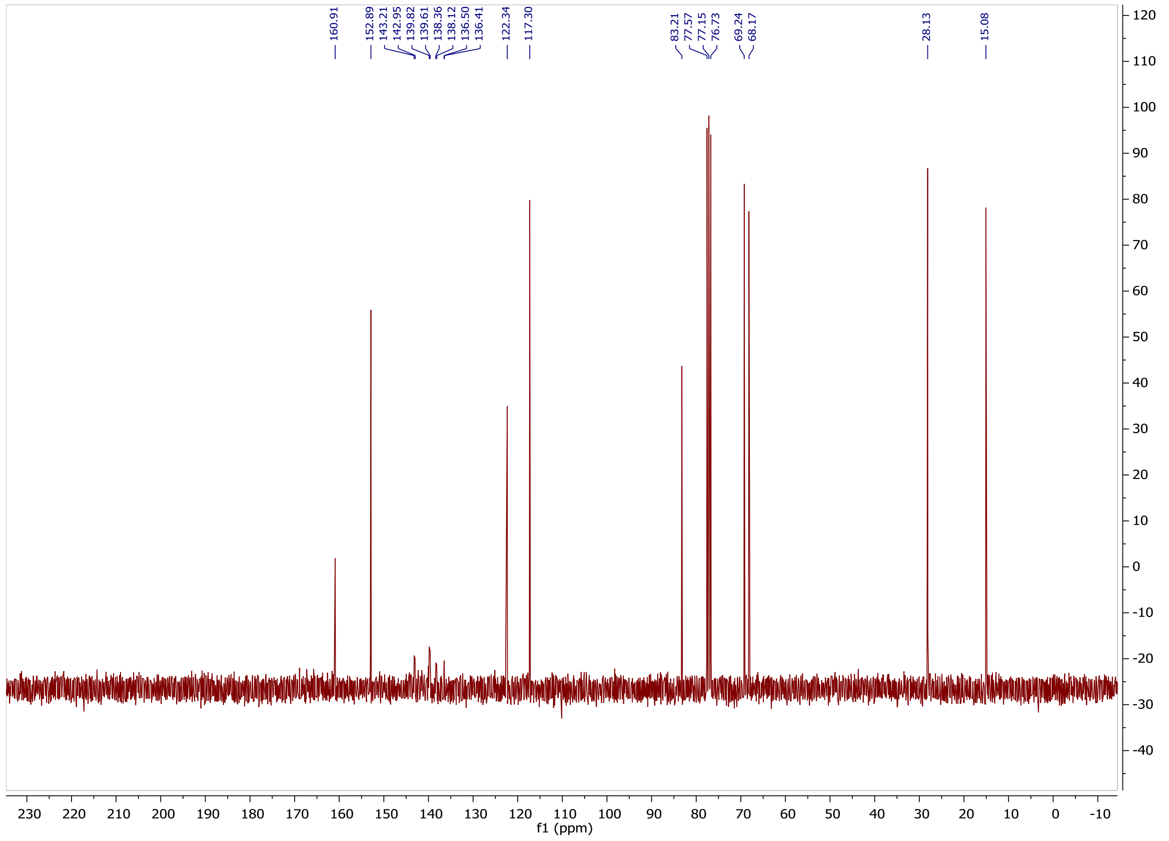

Supplementary figure 36. ^{13}C-NMR spectrum of compound 6a ($CDCl_3$, 75 MHz)

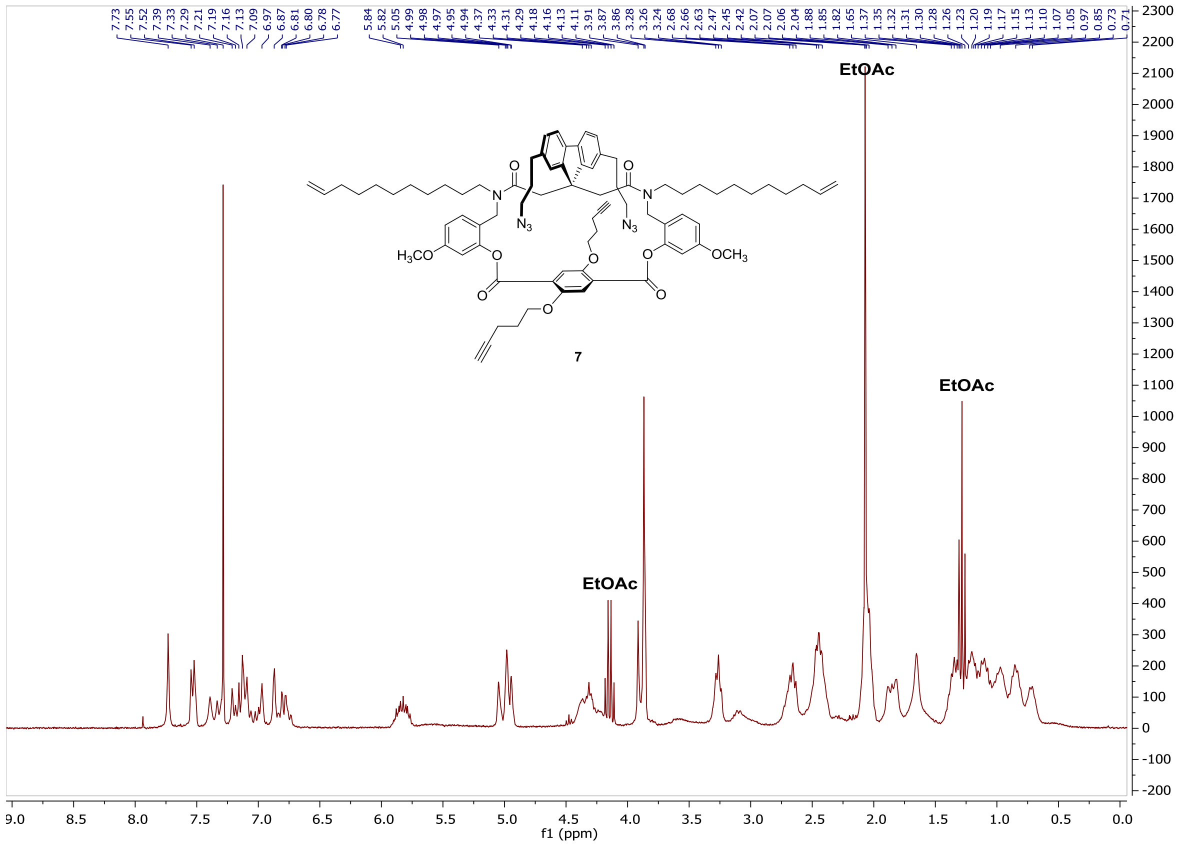

Supplementary figure 37. ^{1}H-NMR spectrum of compound 7 ($CDCl_3$, 300 MHz)

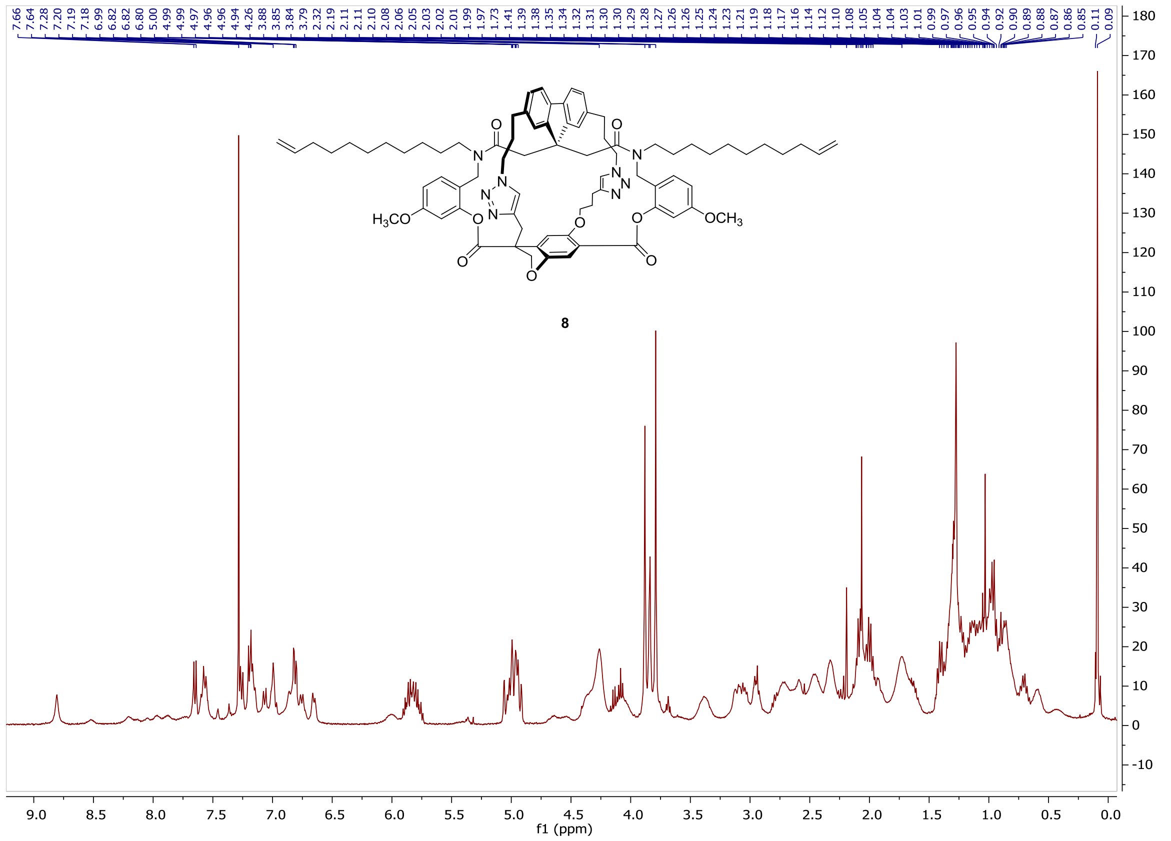

Supplementary figure 38. ^{1}H-NMR spectrum of compound 8 ($CDCl_3$, 300 MHz)

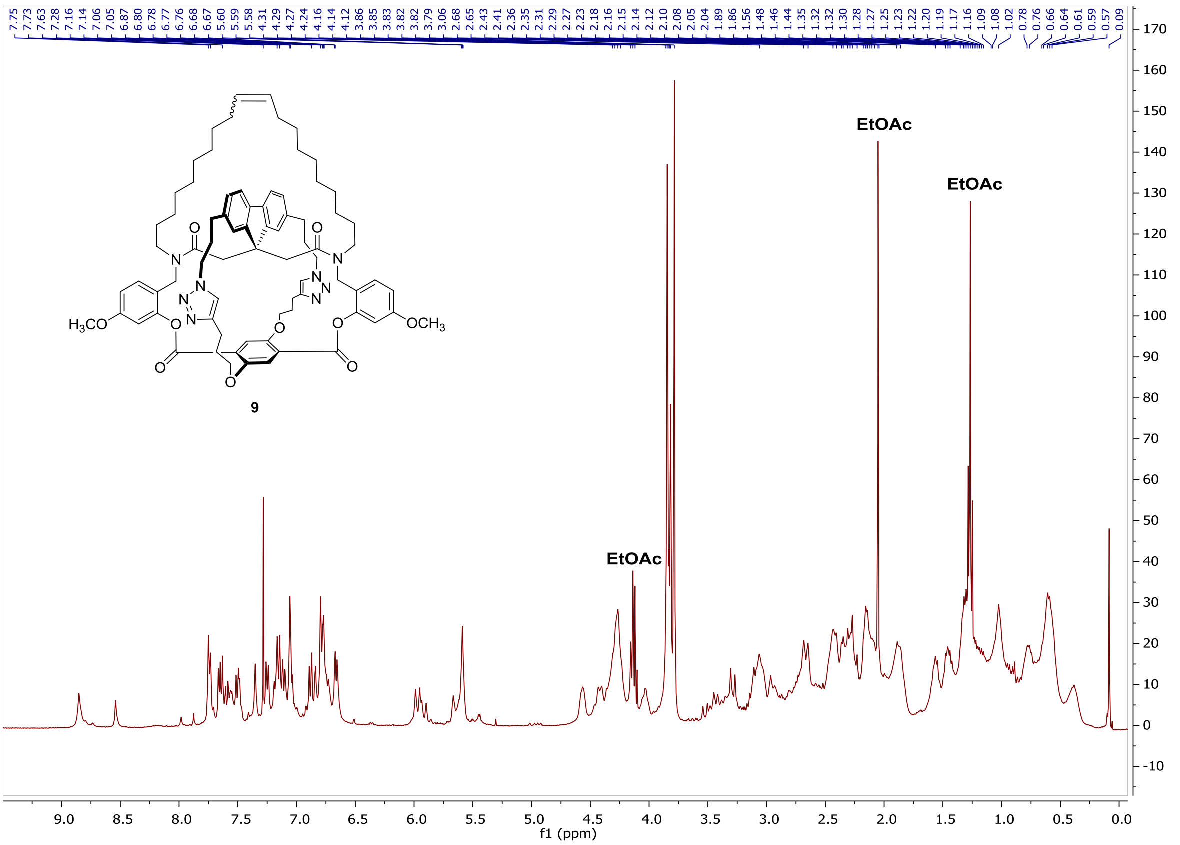

Supplementary figure 40. ^{1}H-NMR spectrum of compound 26 ($CDCl_3$, 300 MHz)

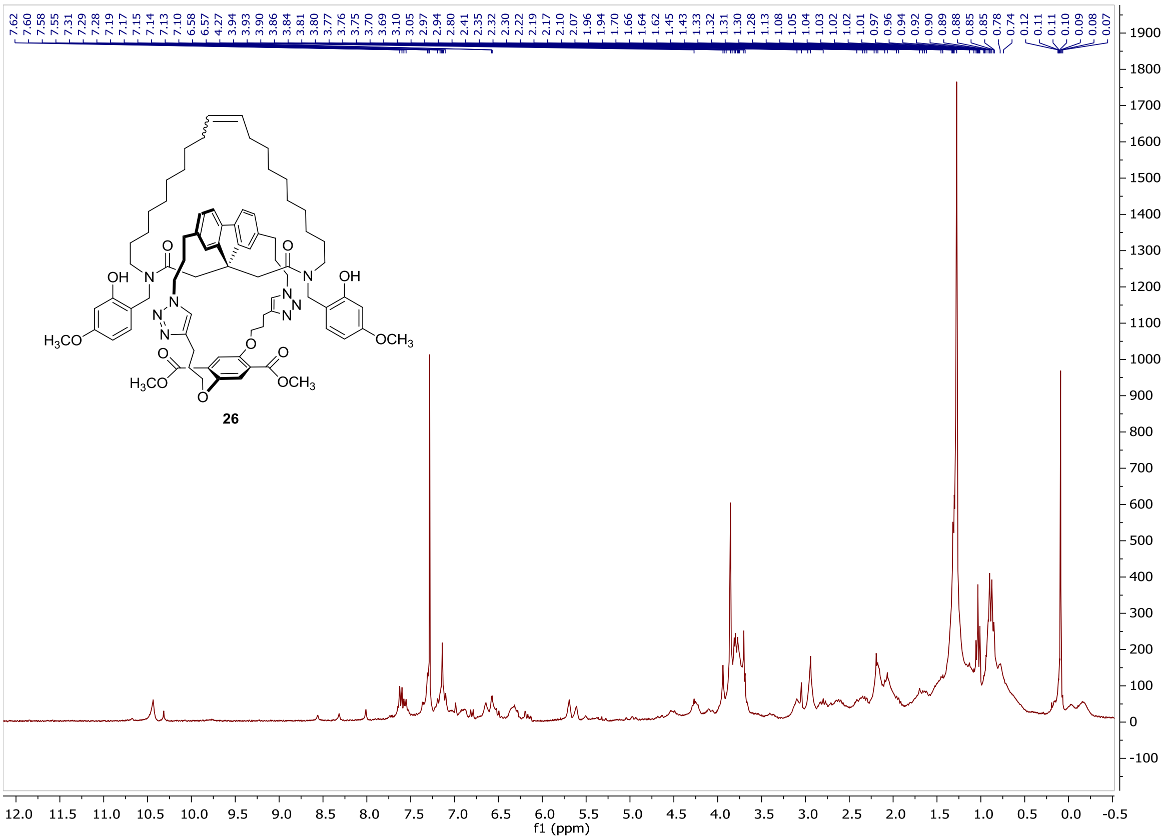

Supplementary figure 39. ^{1}H-NMR spectrum of compound 9 ($CDCl_3$, 300 MHz)

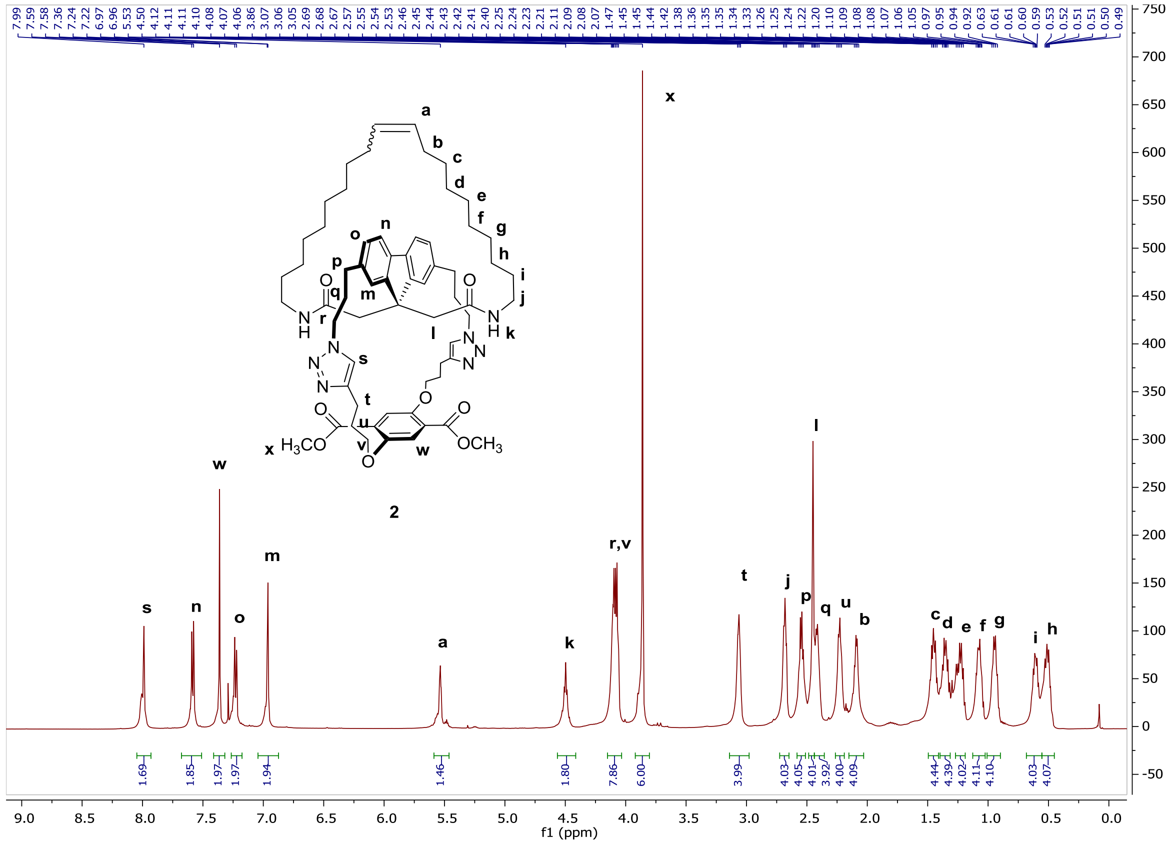

Supplementary figure 41. ^{1}H-NMR spectrum of compound 2 ($CDCl_3$, 500 MHz)

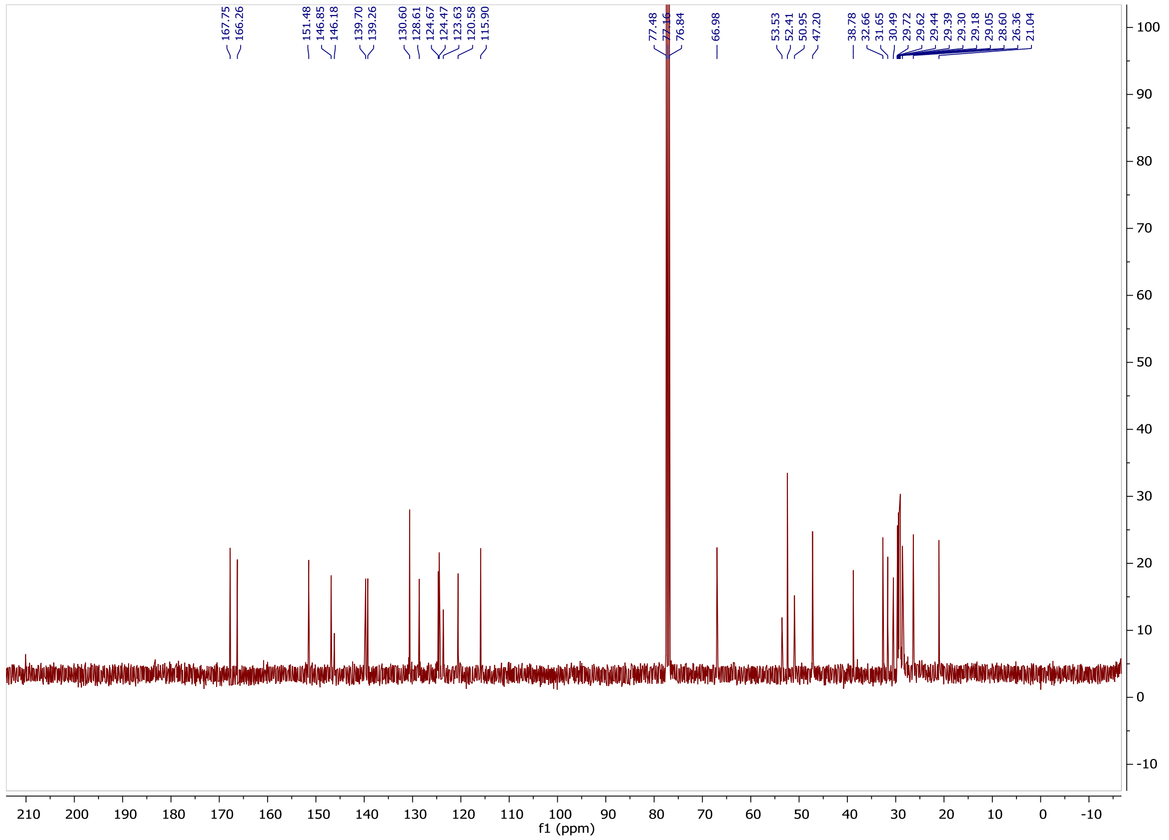

Supplementary figure 42. ^{13}C-NMR spectrum of compound 2 ($CDCl_3$, 125 MHz)

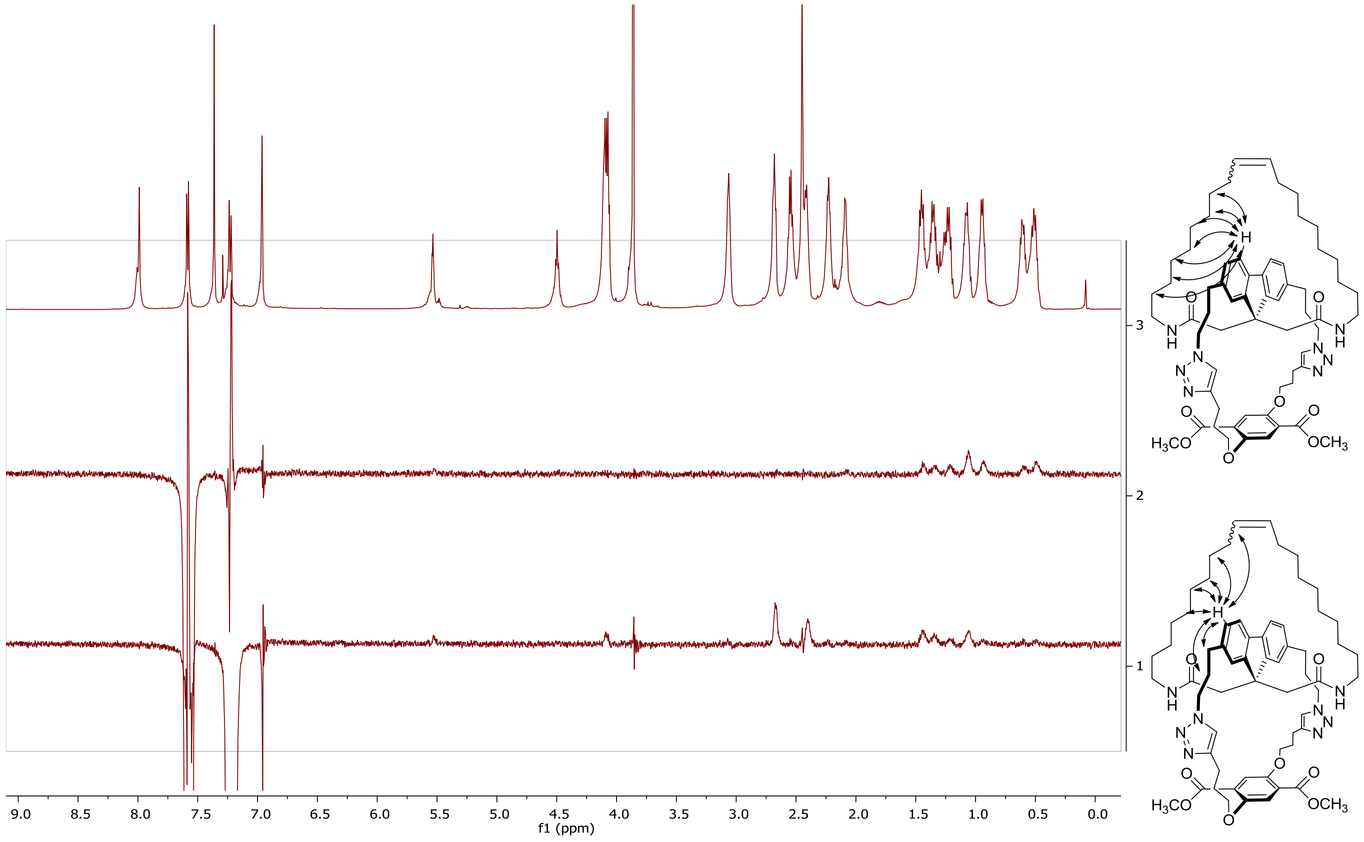

Supplementary figure 43. NOE spectrum of compound 2 ($CDCl_3$, 500 MHz)

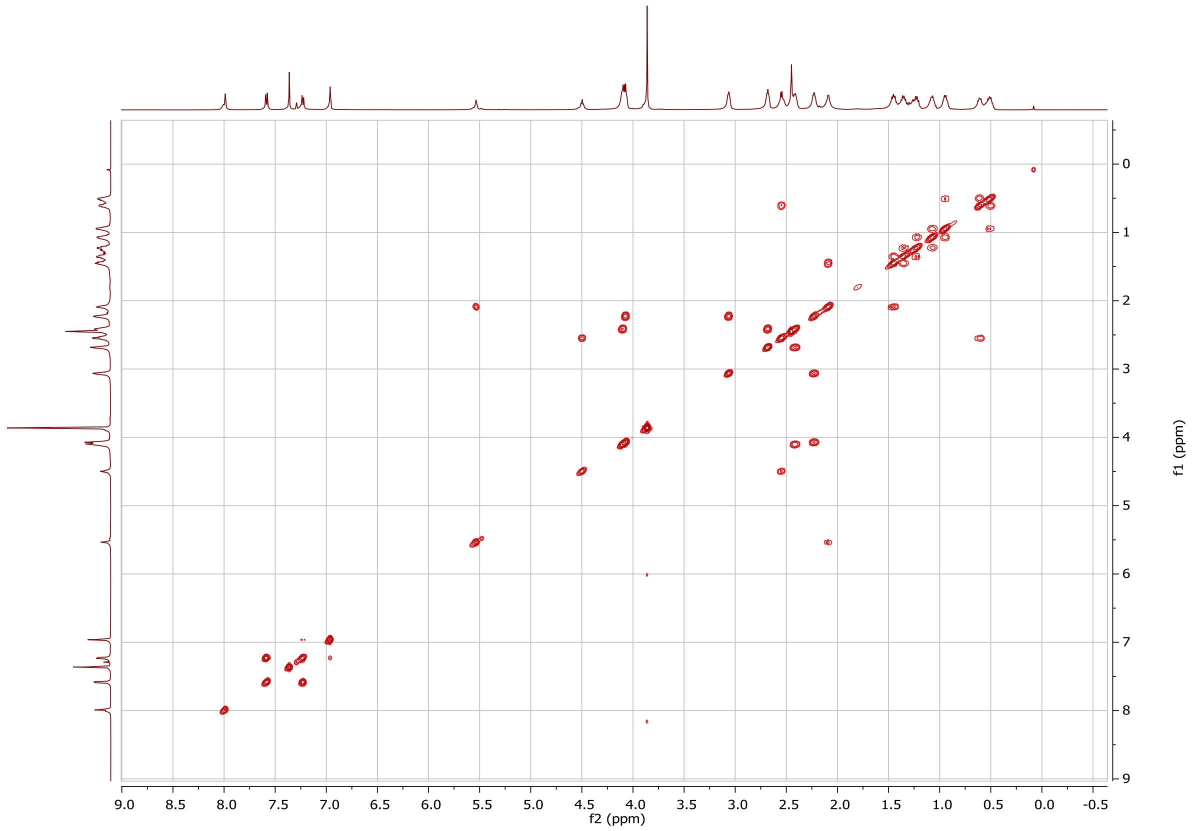

Supplementary figure 44. COSY spectrum of compound 2 ($CDCl_3$, 500 MHz)

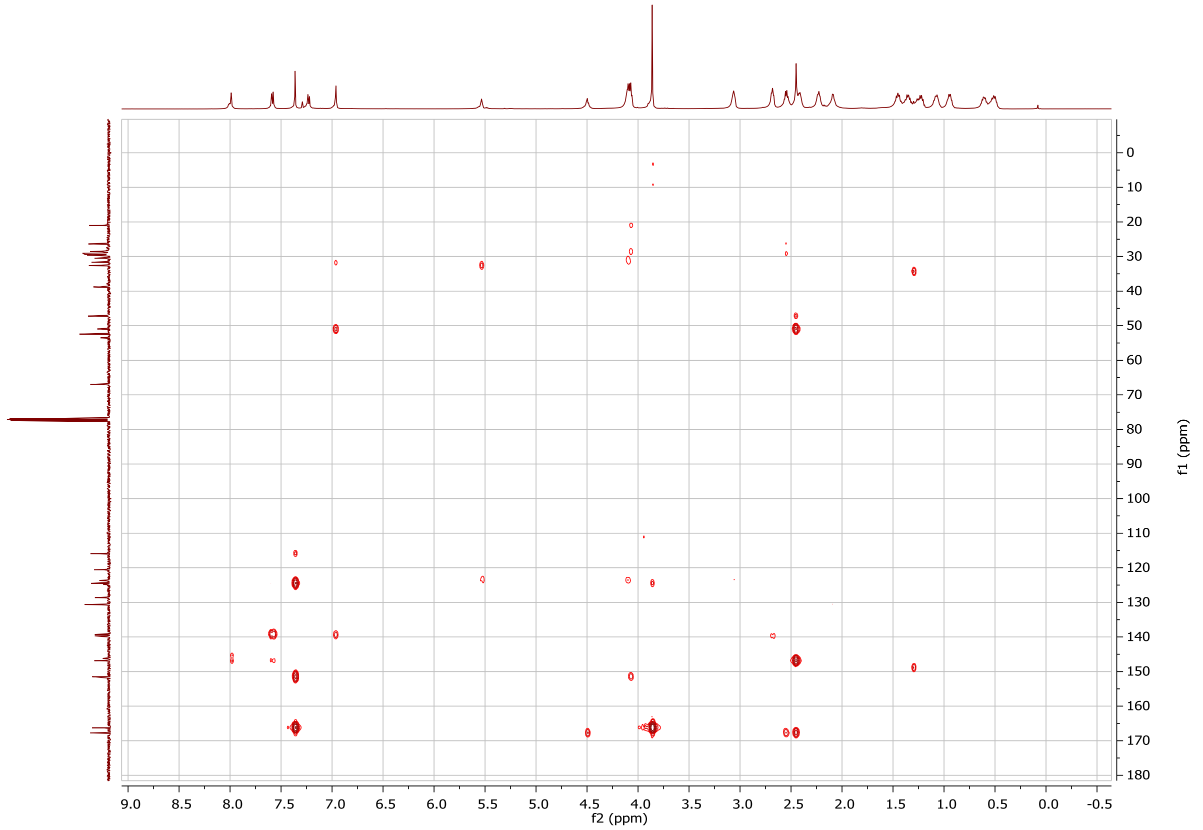

Supplementary figure 45. HMBC spectrum of compound 2 ($CDCl_3$, 500 MHz)

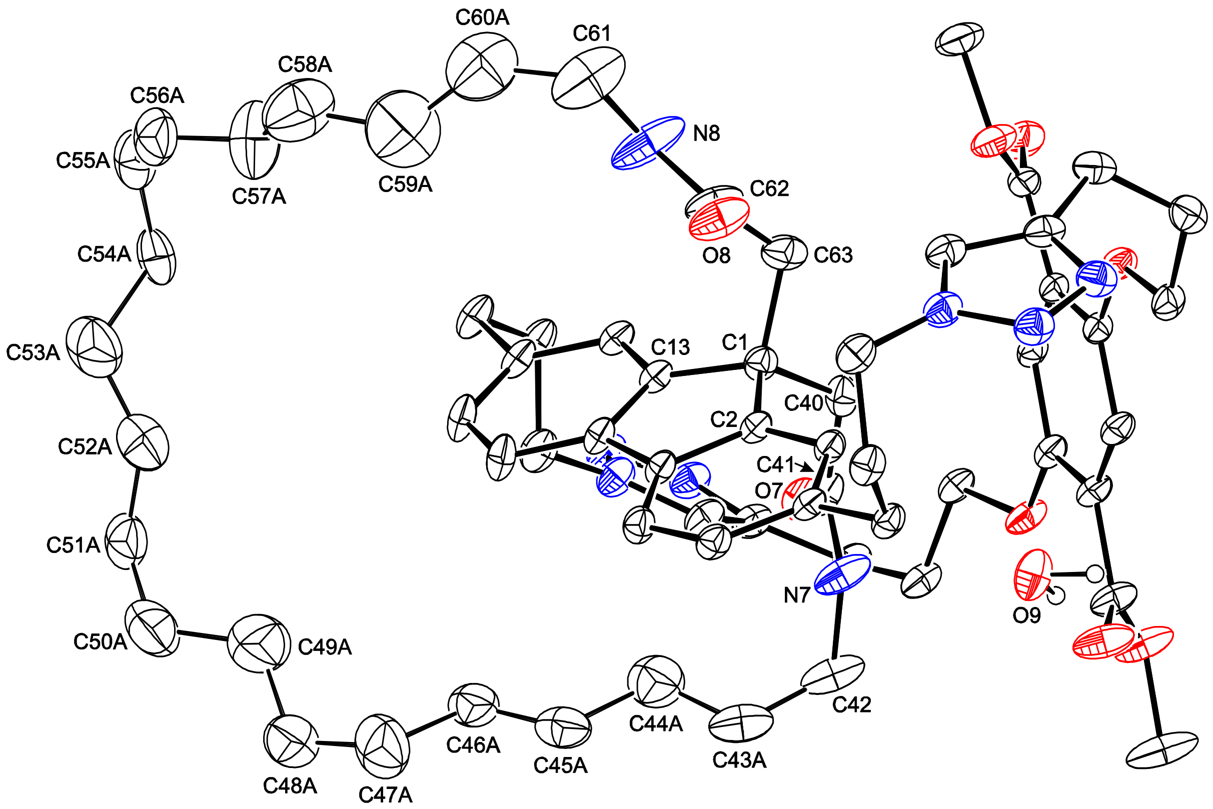

Supplementary figure 46. Displacement ellipsoid plot of l0860c (30% probability level). C-H hydrogen atoms and minor disorder component are omitted for clarity.

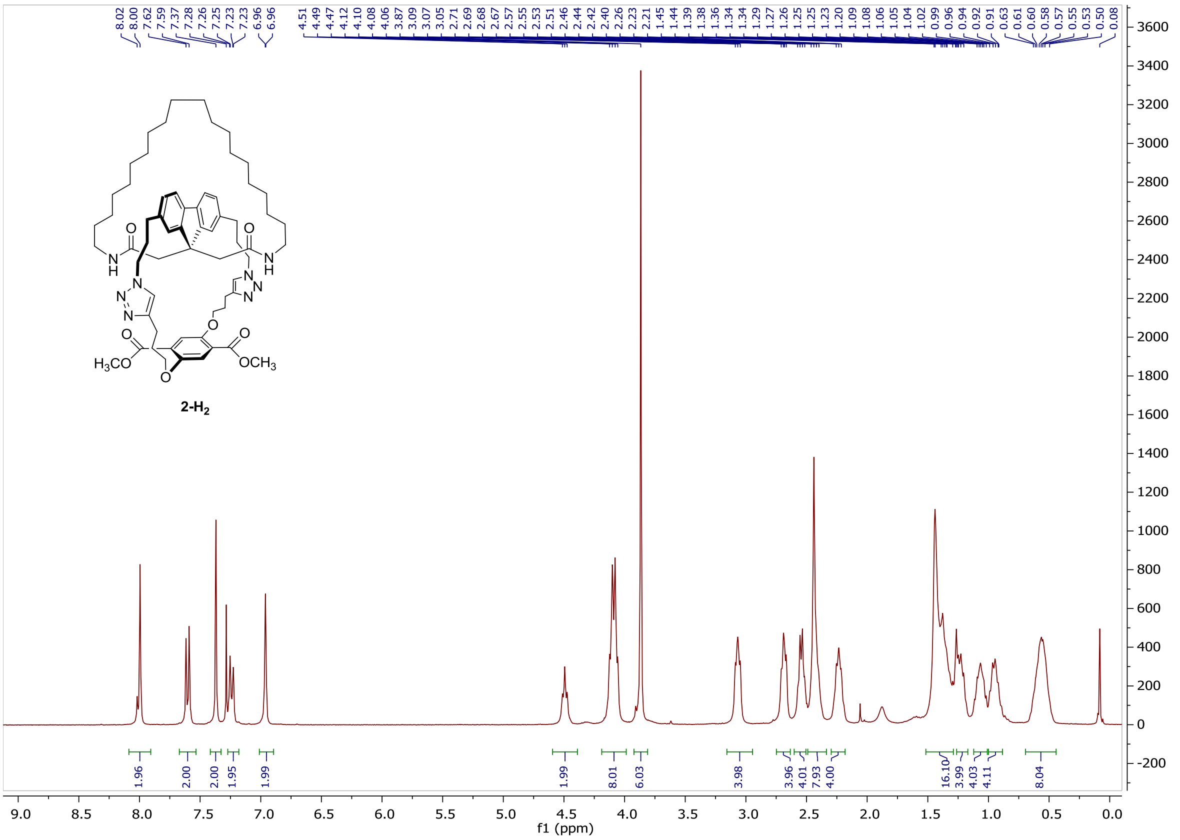

Supplementary figure 47. **^{1}H-NMR spectrum of compound 2-H_2 ($CDCl_3$, 300 MHz)**

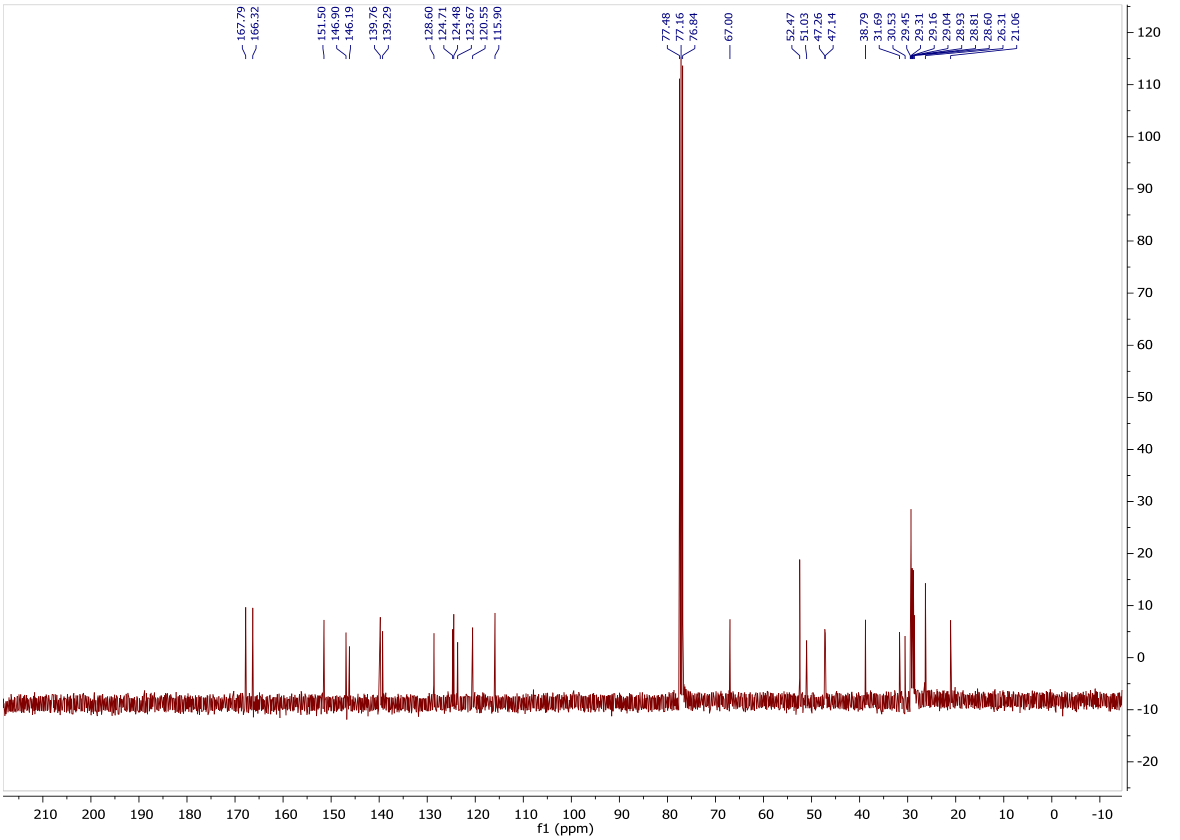

Supplementary figure 48. **^{13}C-NMR spectrum of compound 2-H_2 ($CDCl_3$, 75 MHz)**

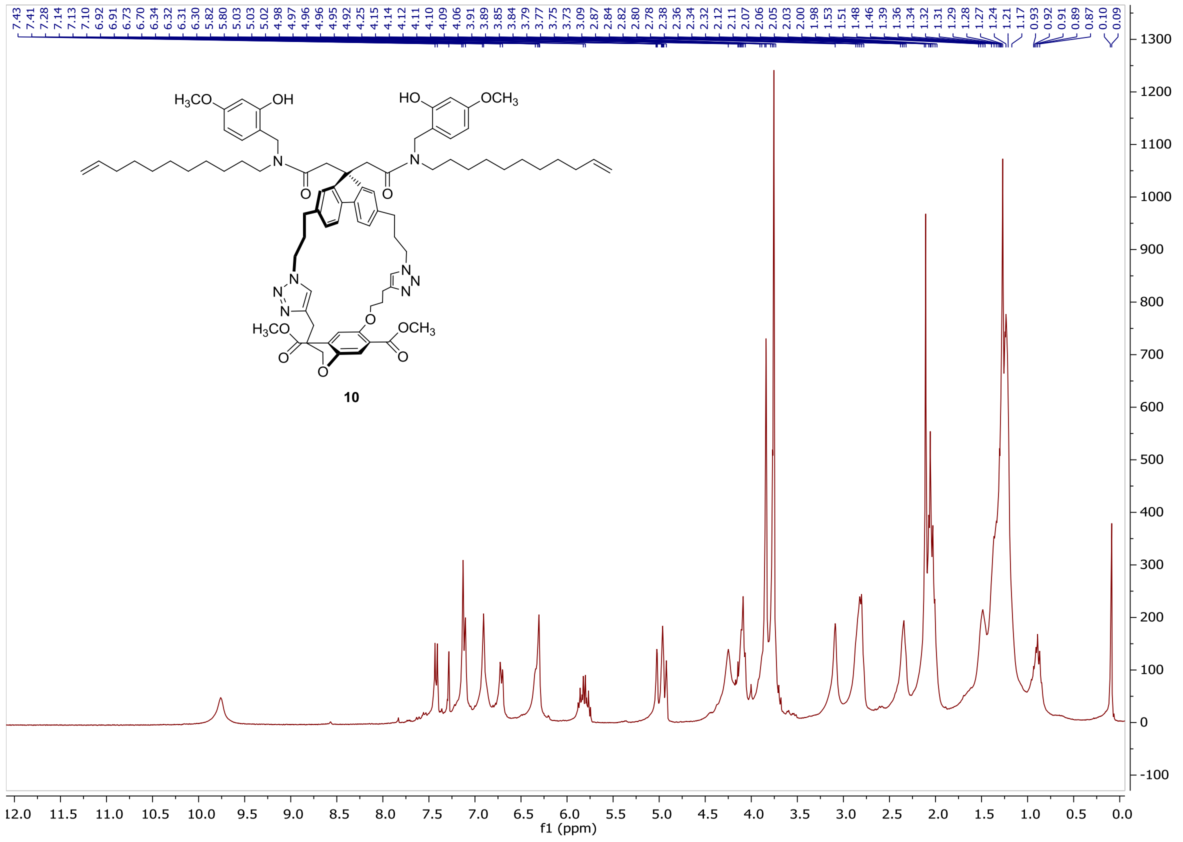

Supplementary figure 49. ^{1}H-NMR spectrum of compound 10 ($CDCl_3$, 300 MHz)

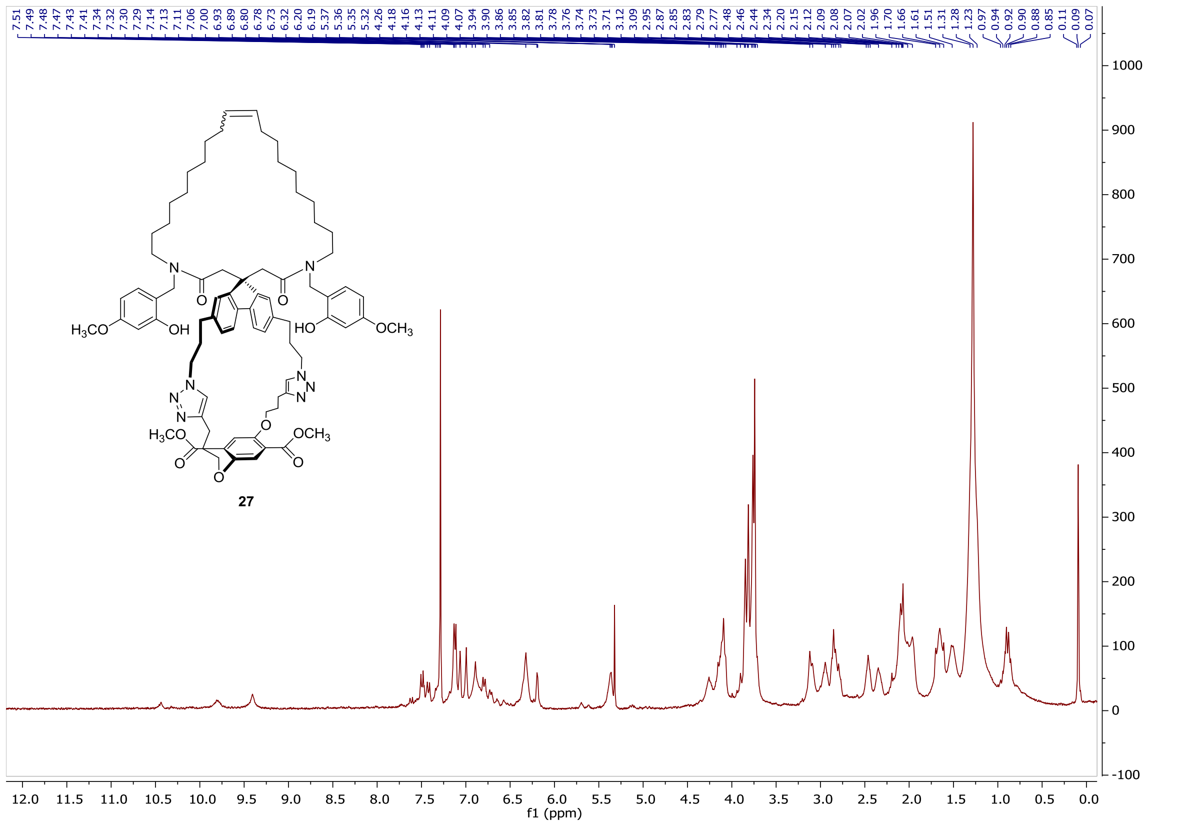

Supplementary figure 50. ^{1}H-NMR spectrum of compound 27 ($CDCl_3$, 300 MHz)

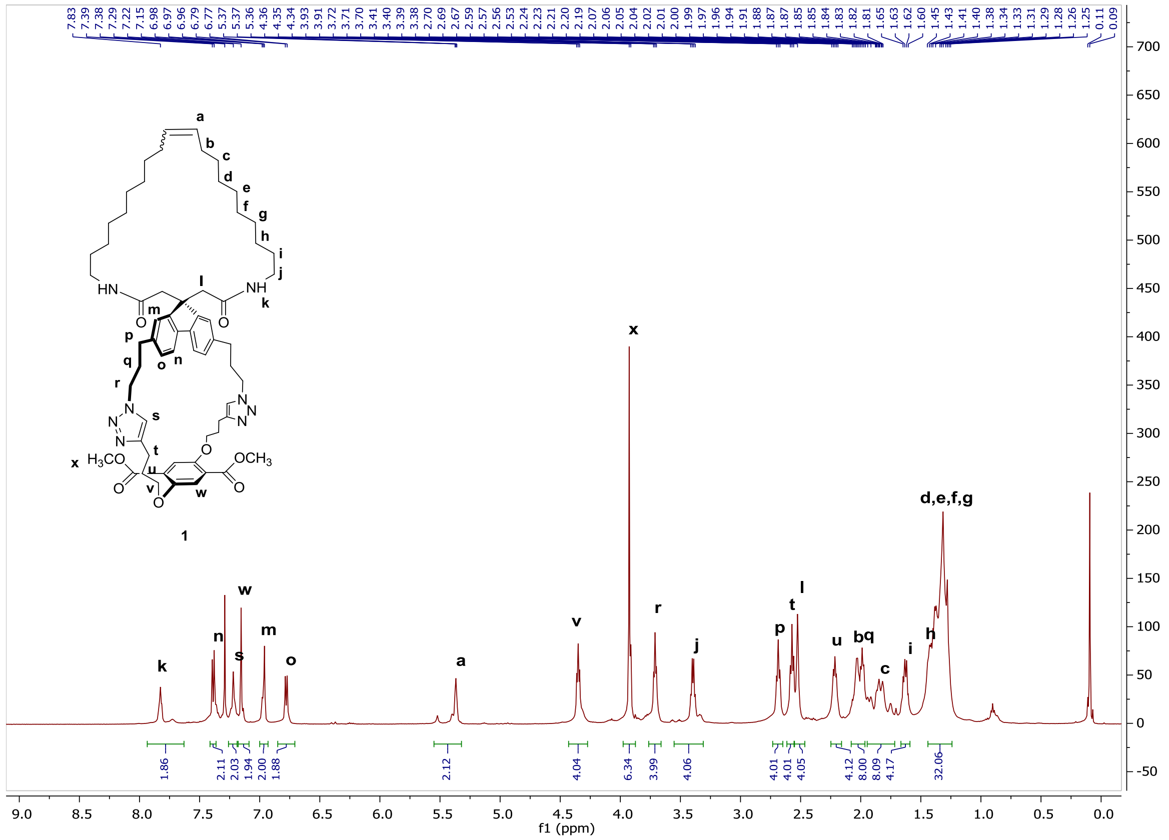

Supplementary figure 51. ^{1}H-NMR spectrum of compound 1 ($CDCl_3$, 500 MHz)

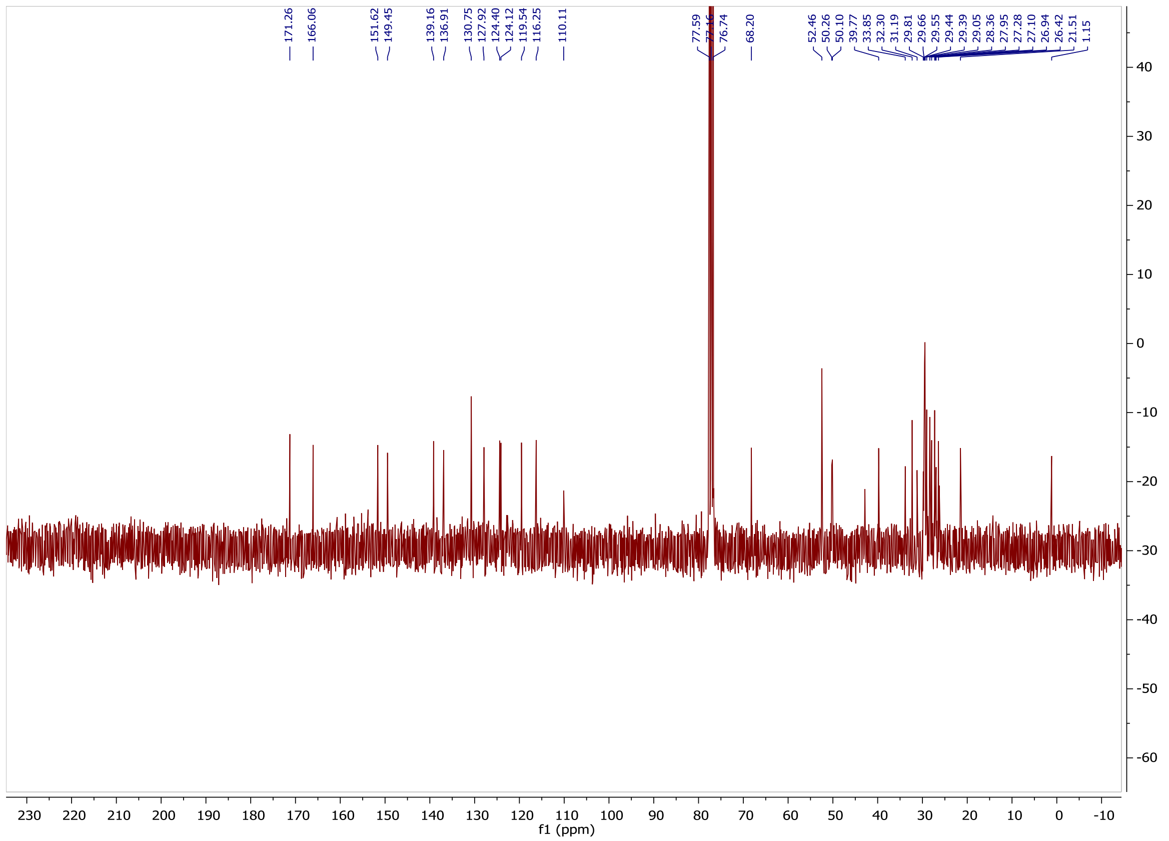

Supplementary figure 52. ^{13}C-NMR spectrum of compound 1 ($CDCl_3$, 125 MHz)

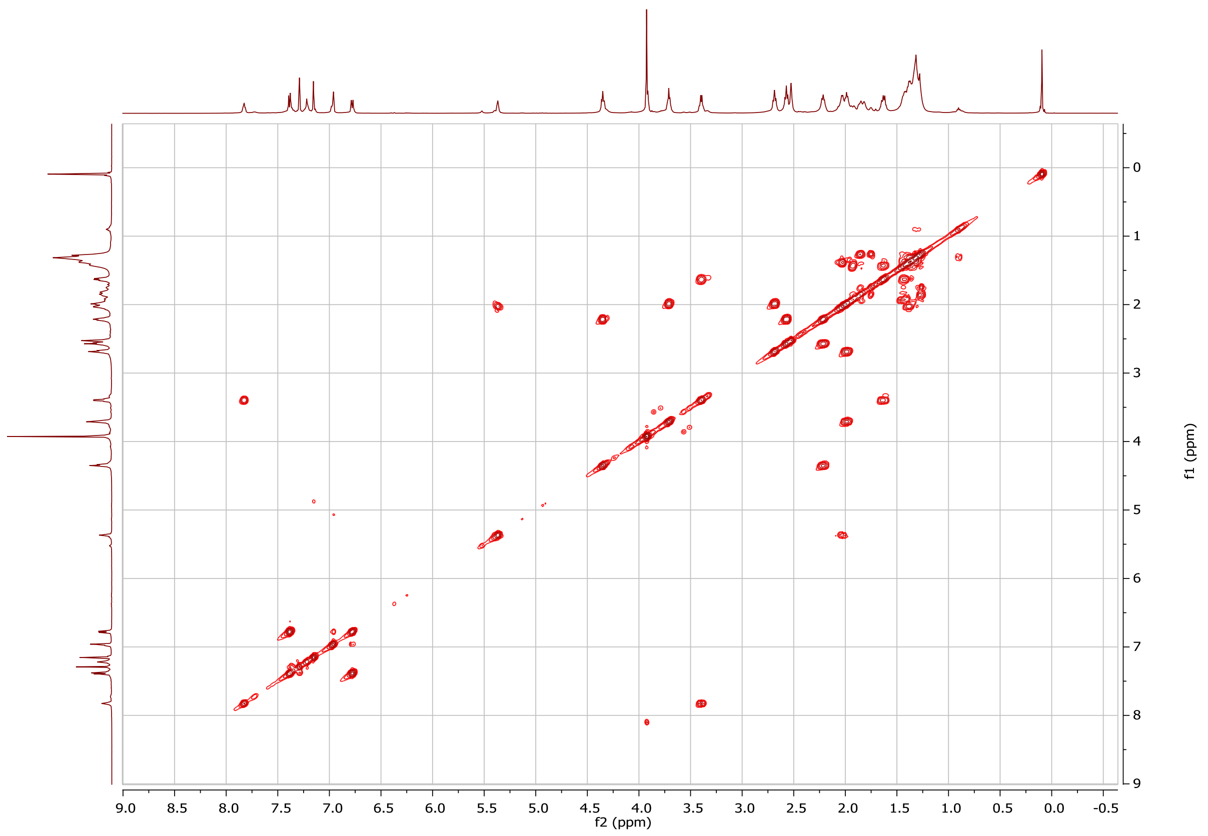

Supplementary figure 53. COSY spectrum of compound 1 ($CDCl_3$, 500 MHz)

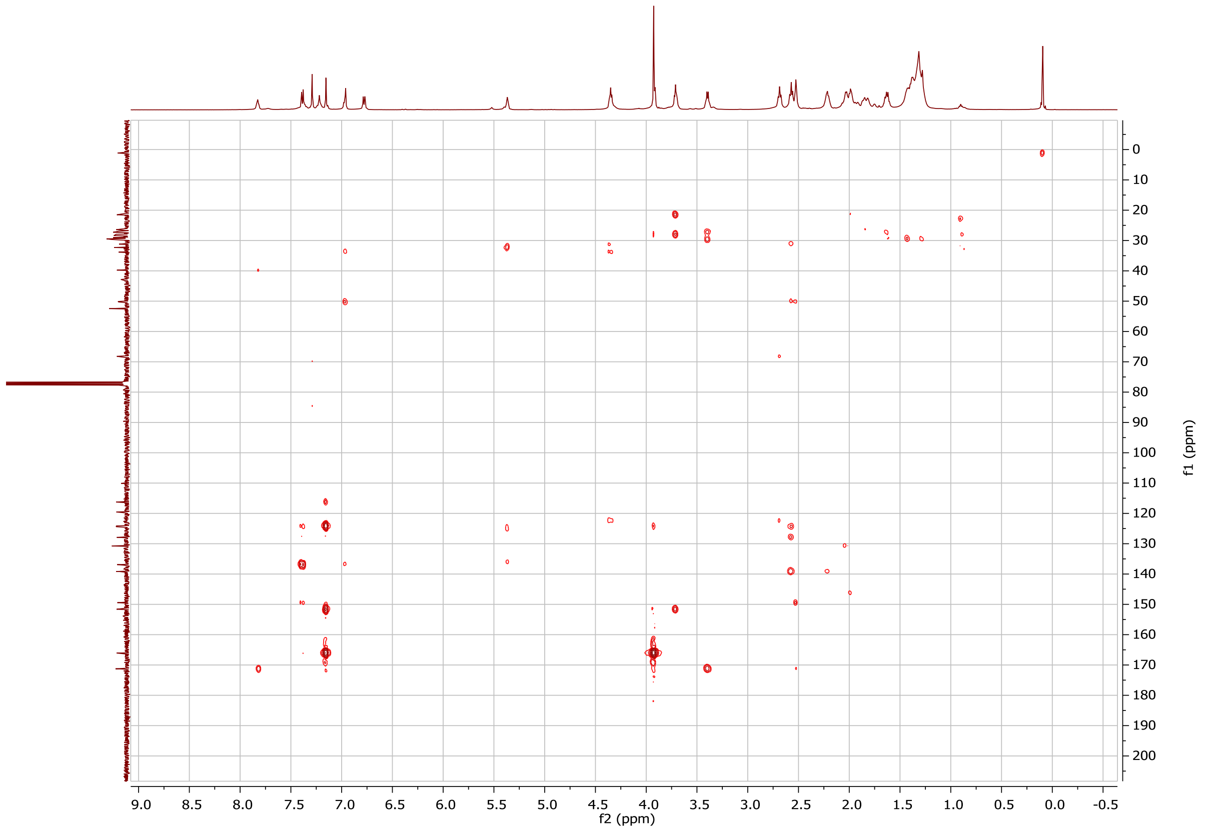

Supplementary figure 54. HMBC spectrum of compound 1 ($CDCl_3$, 500 MHz)

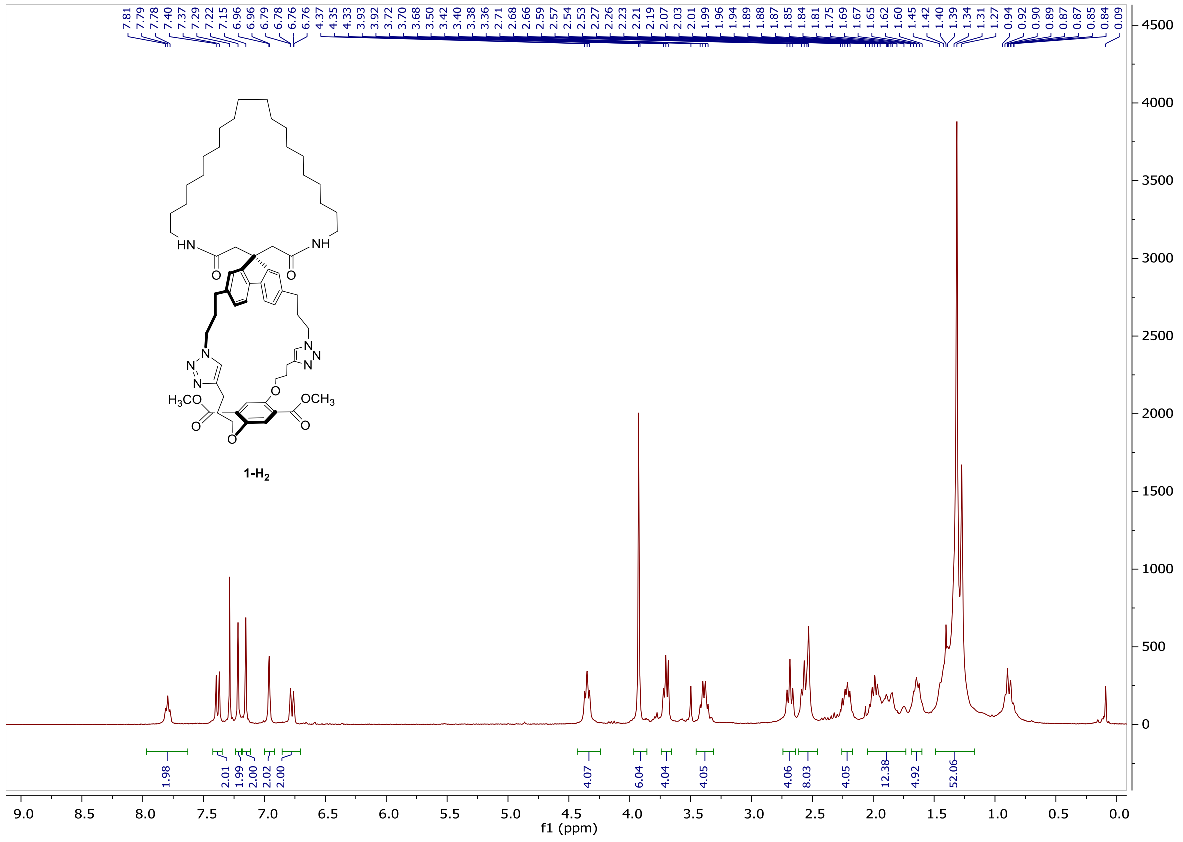

Supplementary figure 55. ^{1}**H-NMR spectrum of compound 1-H$_2$ (CDCl$_3$, 300 MHz)**

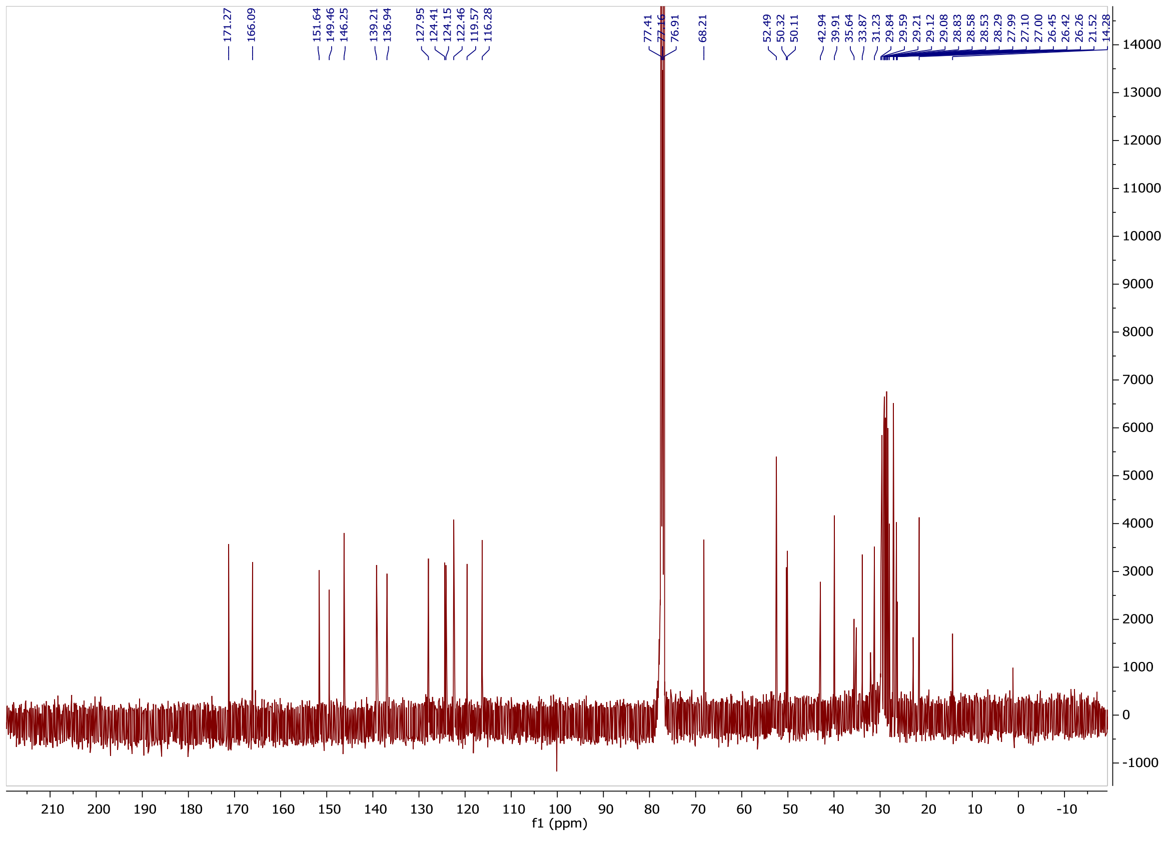

Supplementary figure 56. ^{13}**C-NMR spectrum of compound 1-H$_2$ (CDCl$_3$, 75 MHz)**

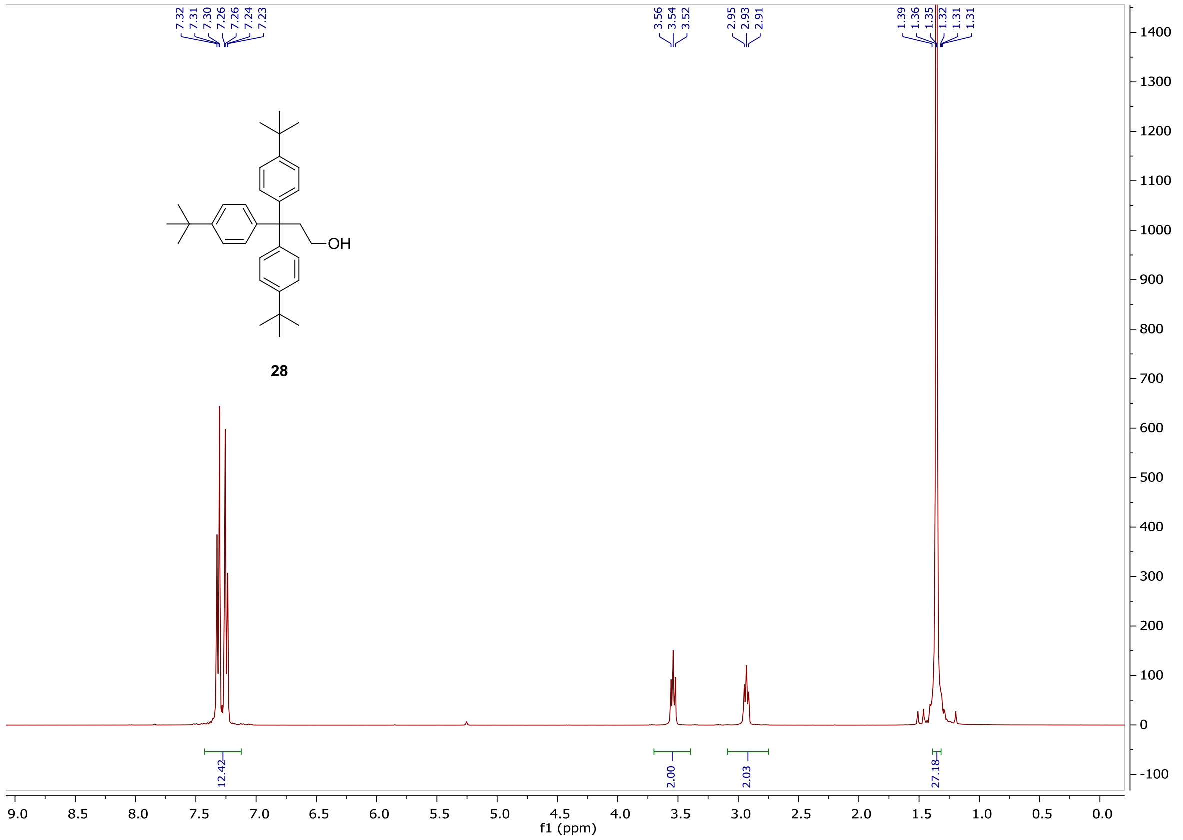

Supplementary figure 57. ^{1}H-NMR spectrum of compound 28 ($CDCl_3$, 300 MHz)

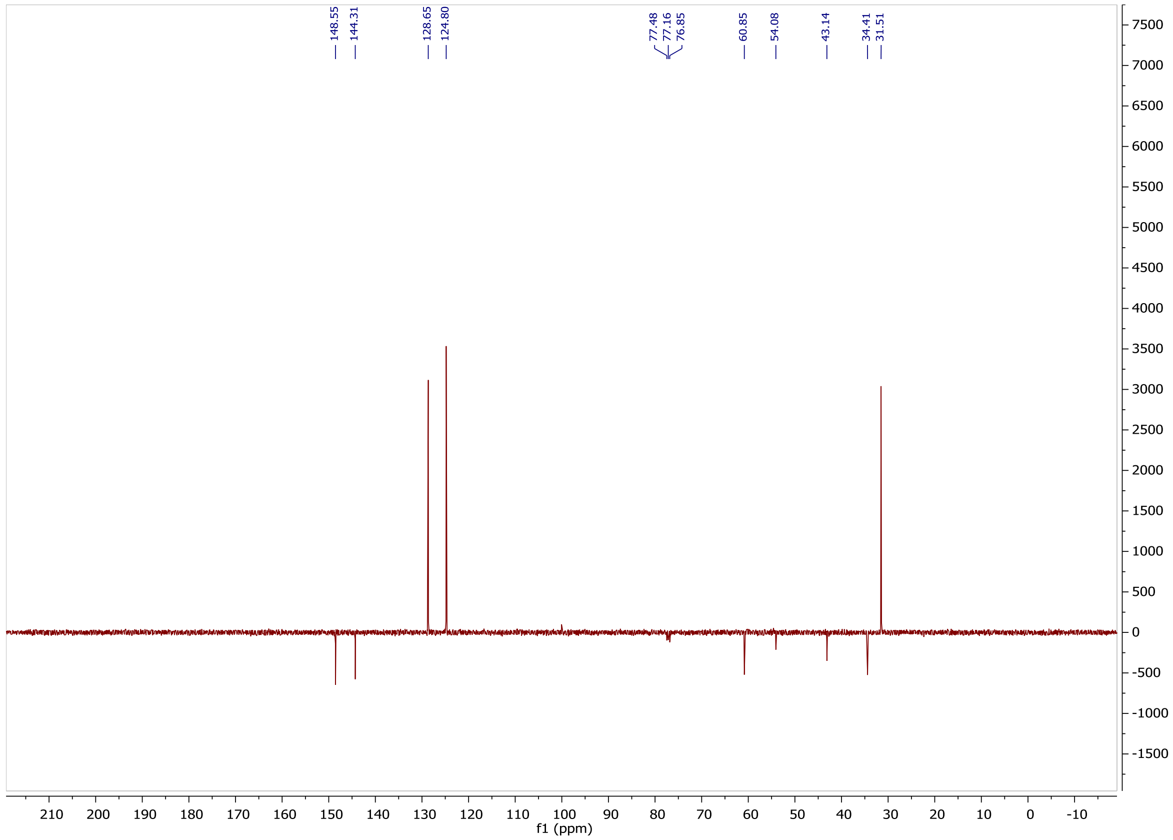

Supplementary figure 58. ^{13}C-NMR spectrum of compound 28 ($CDCl_3$, 75 MHz)

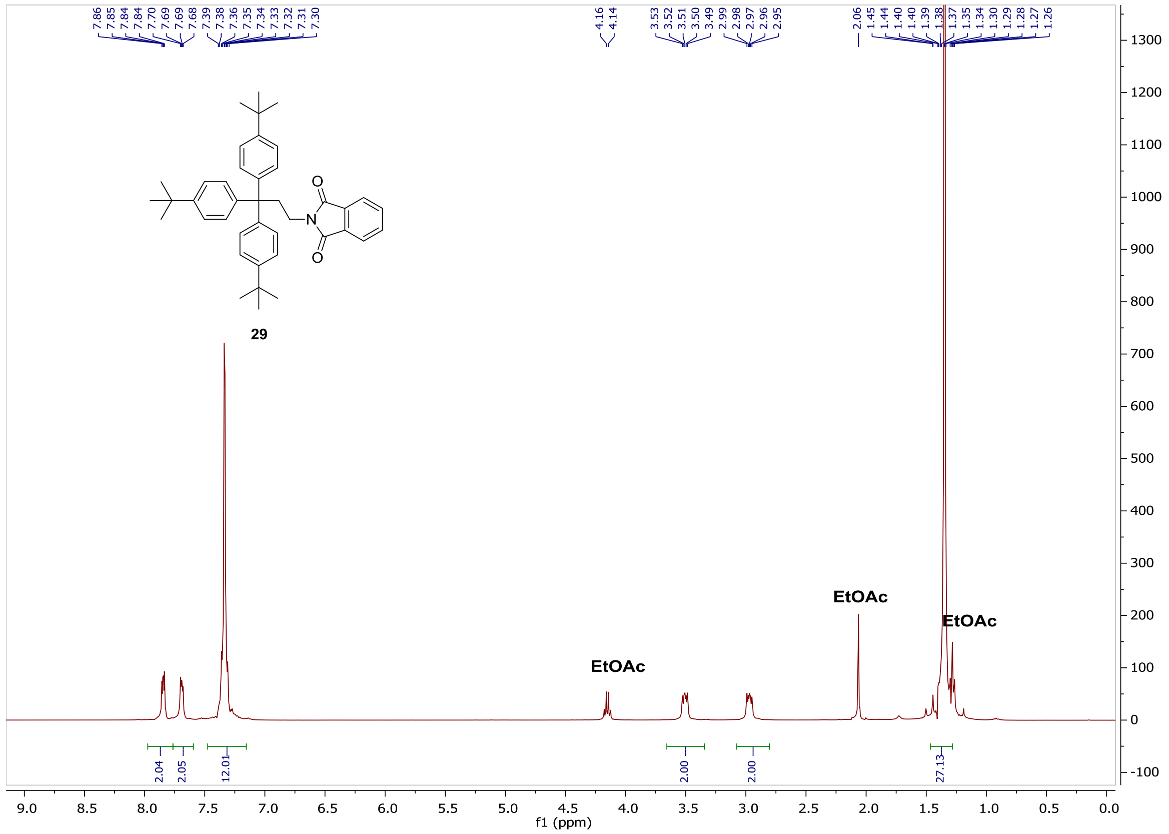

Supplementary figure 59. **^{1}H-NMR spectrum of compound 29 ($CDCl_3$, 300 MHz)**

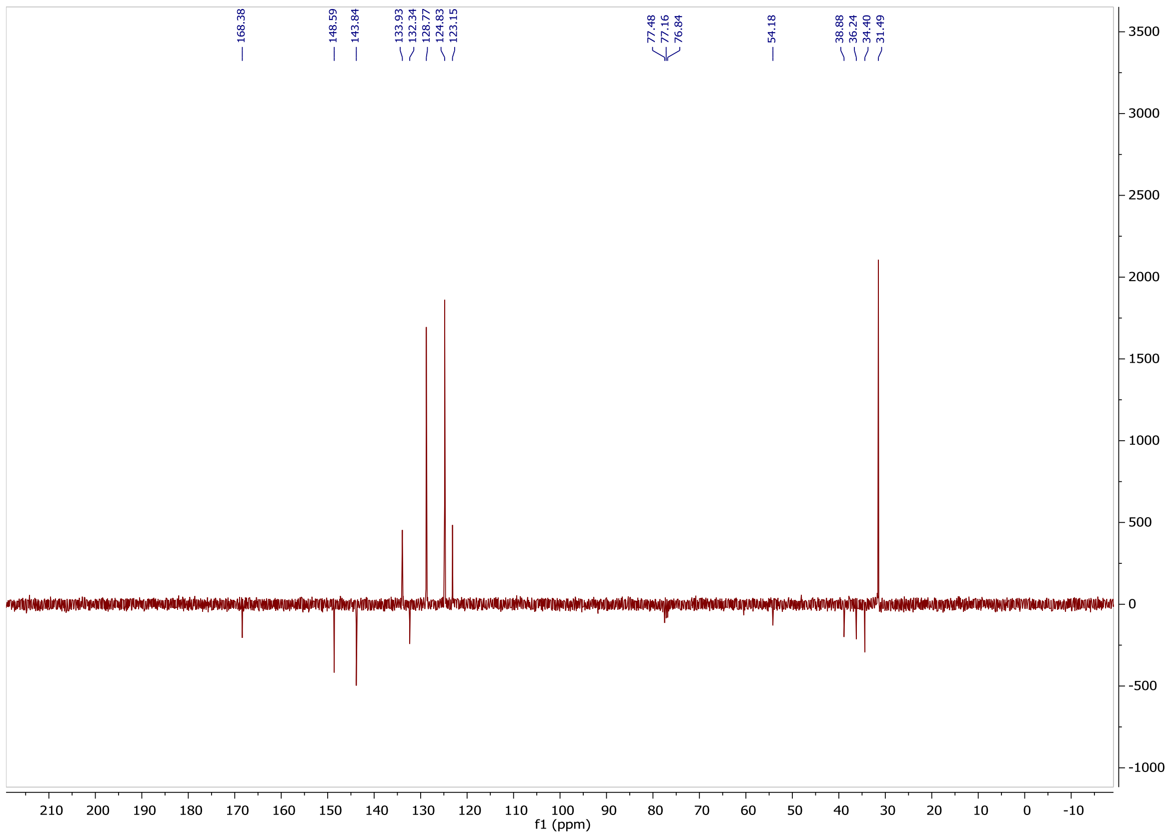

Supplementary figure 60. **^{13}C-NMR spectrum of compound 29 ($CDCl_3$, 75 MHz)**

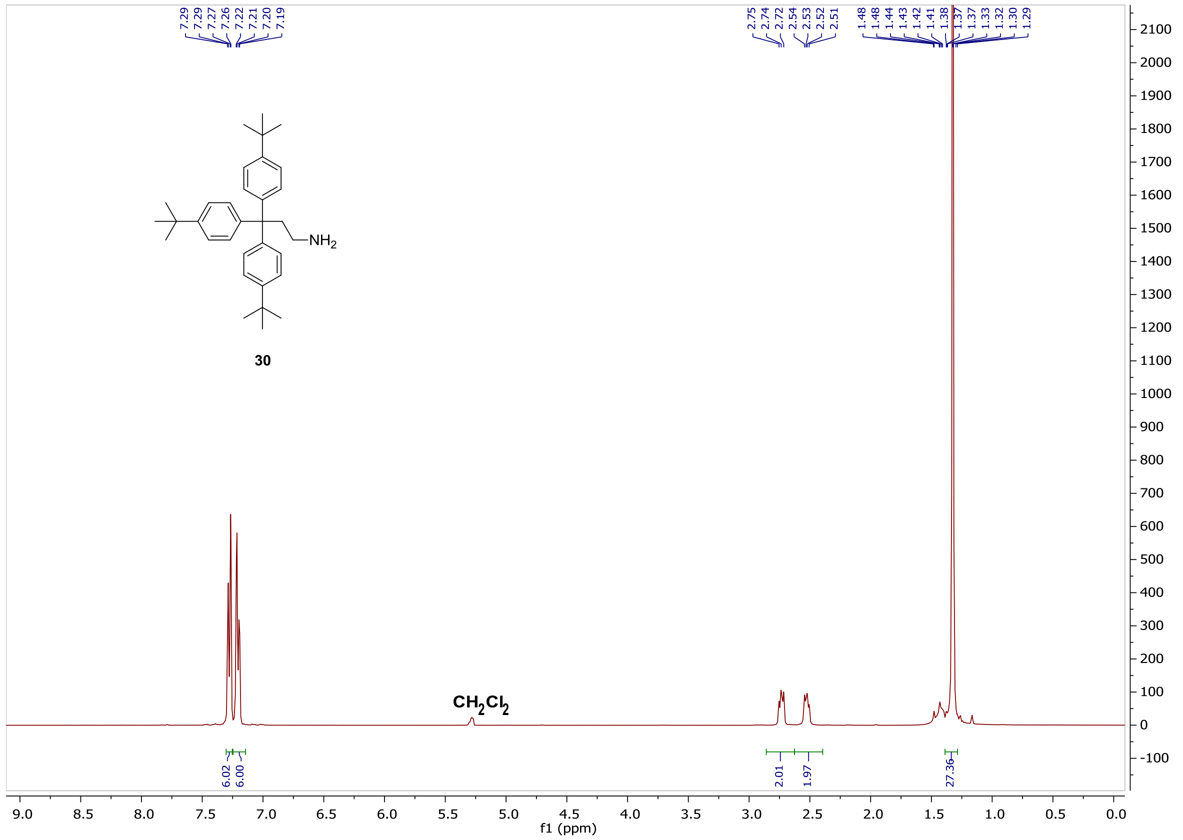

Supplementary figure 61. ^{1}**H-NMR spectrum of compound 30 ($CDCl_3$, 300 MHz)**

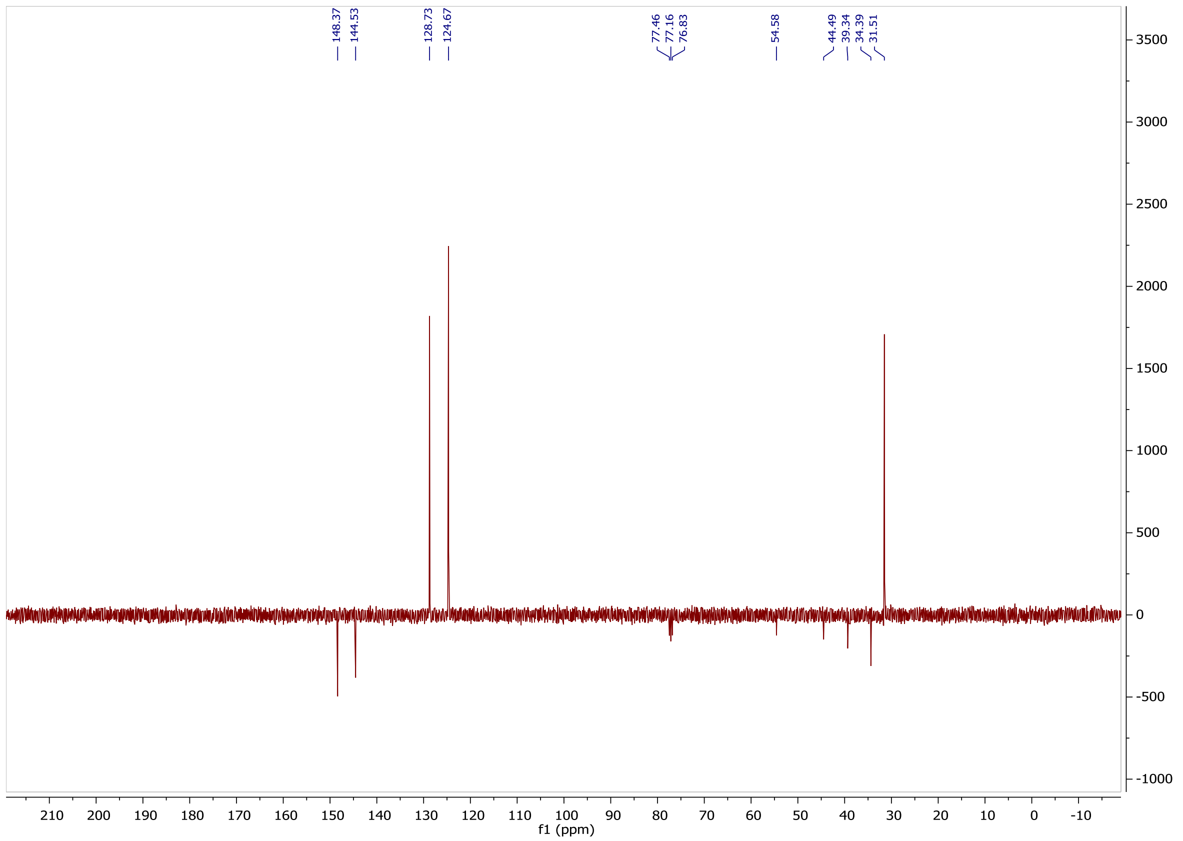

Supplementary figure 62. ^{13}**C-NMR spectrum of compound 30 ($CDCl_3$, 75 MHz)**

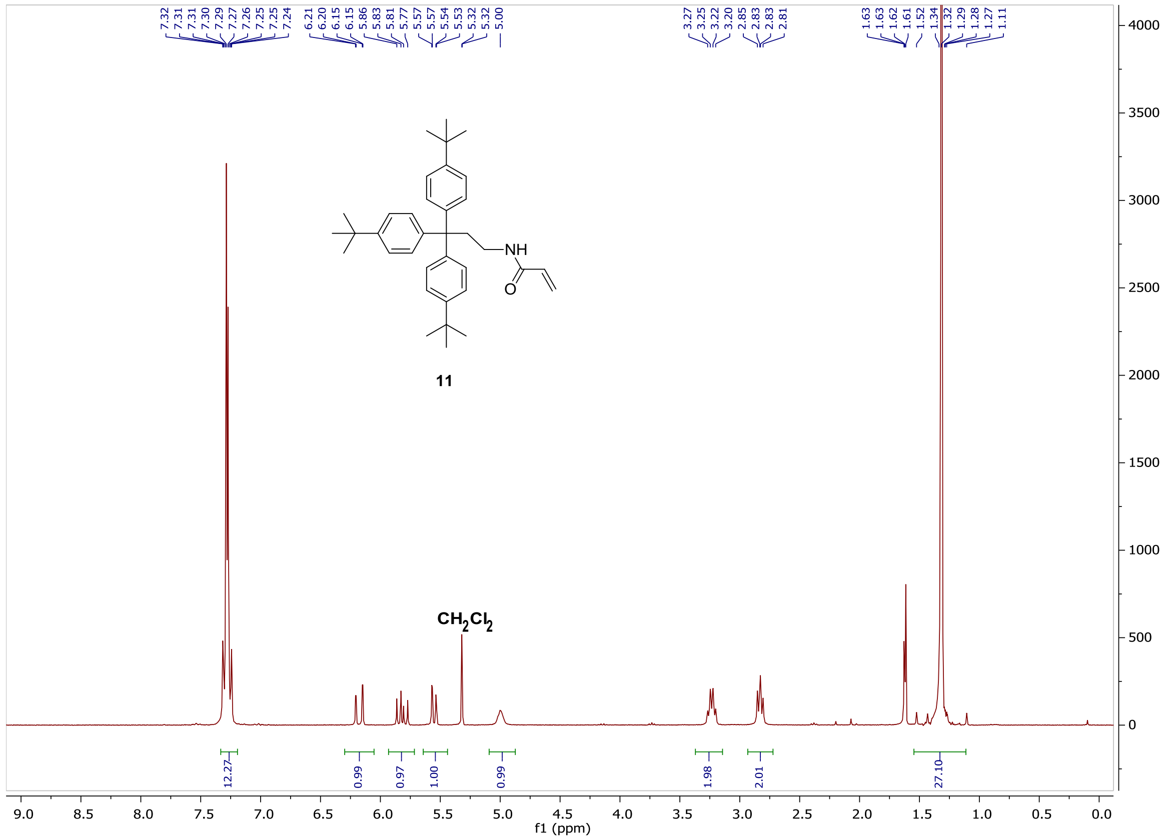

Supplementary figure 63. ^{1}H-NMR spectrum of compound 11 ($CDCl_3$, 300 MHz)

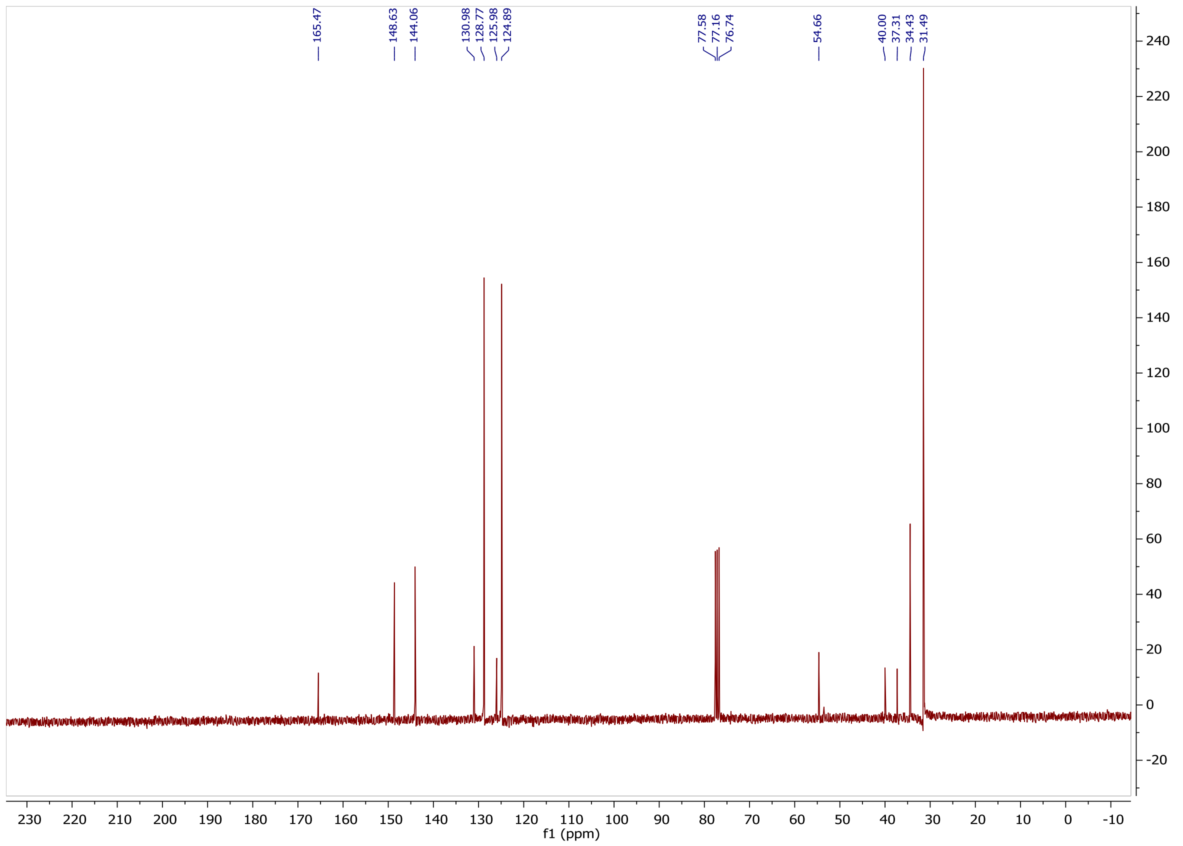

Supplementary figure 64. ^{13}C-NMR spectrum of compound 11 ($CDCl_3$, 75 MHz)

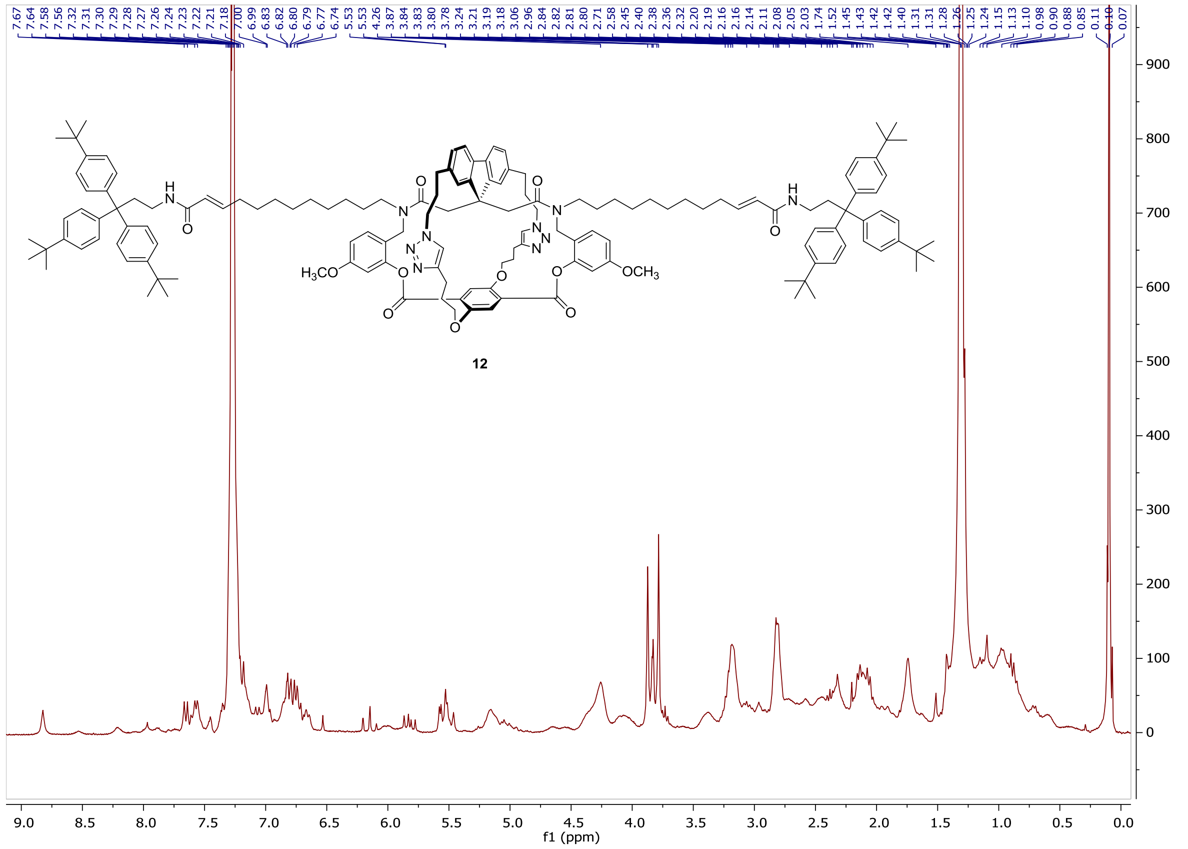

Supplementary figure 65. [1]**H-NMR spectrum of compound 12 (CDCl$_3$, 300 MHz)**

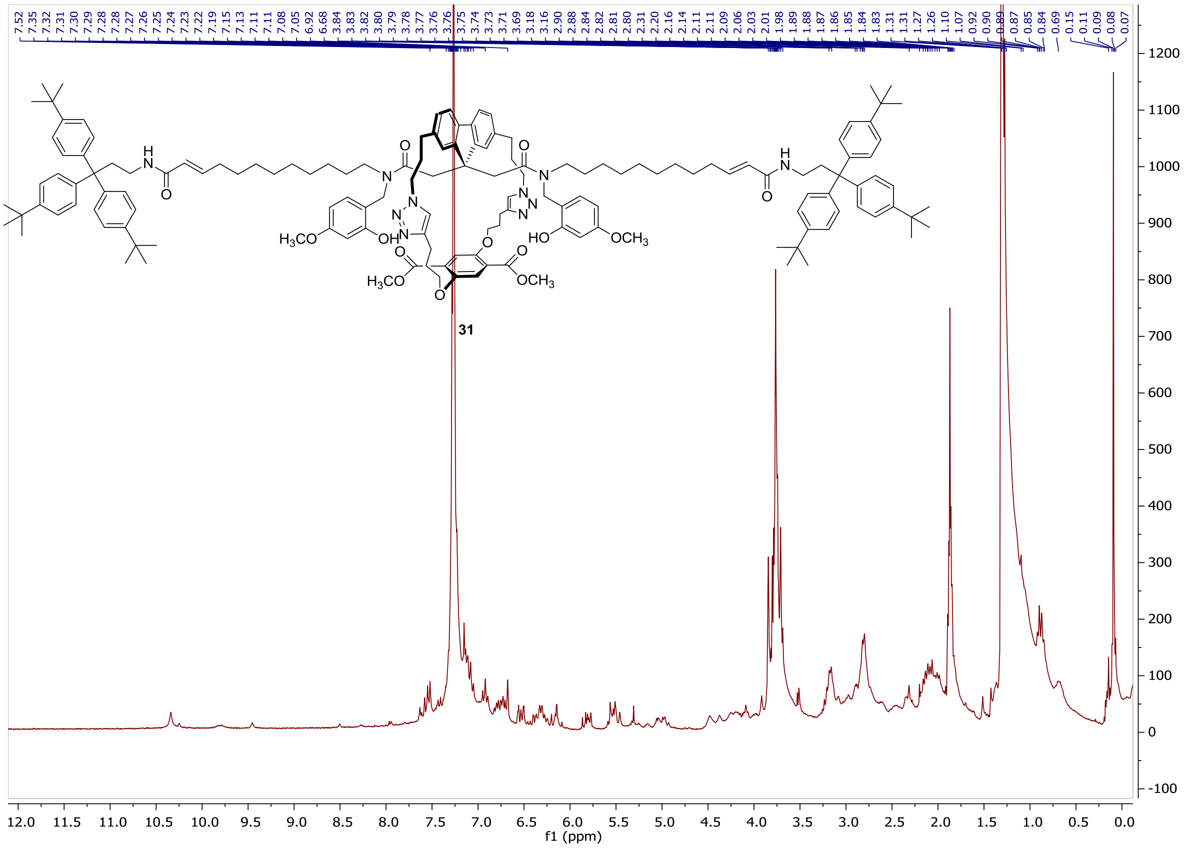

Supplementary figure 66. [1]**H-NMR spectrum of compound 31 (CDCl$_3$, 300 MHz)**

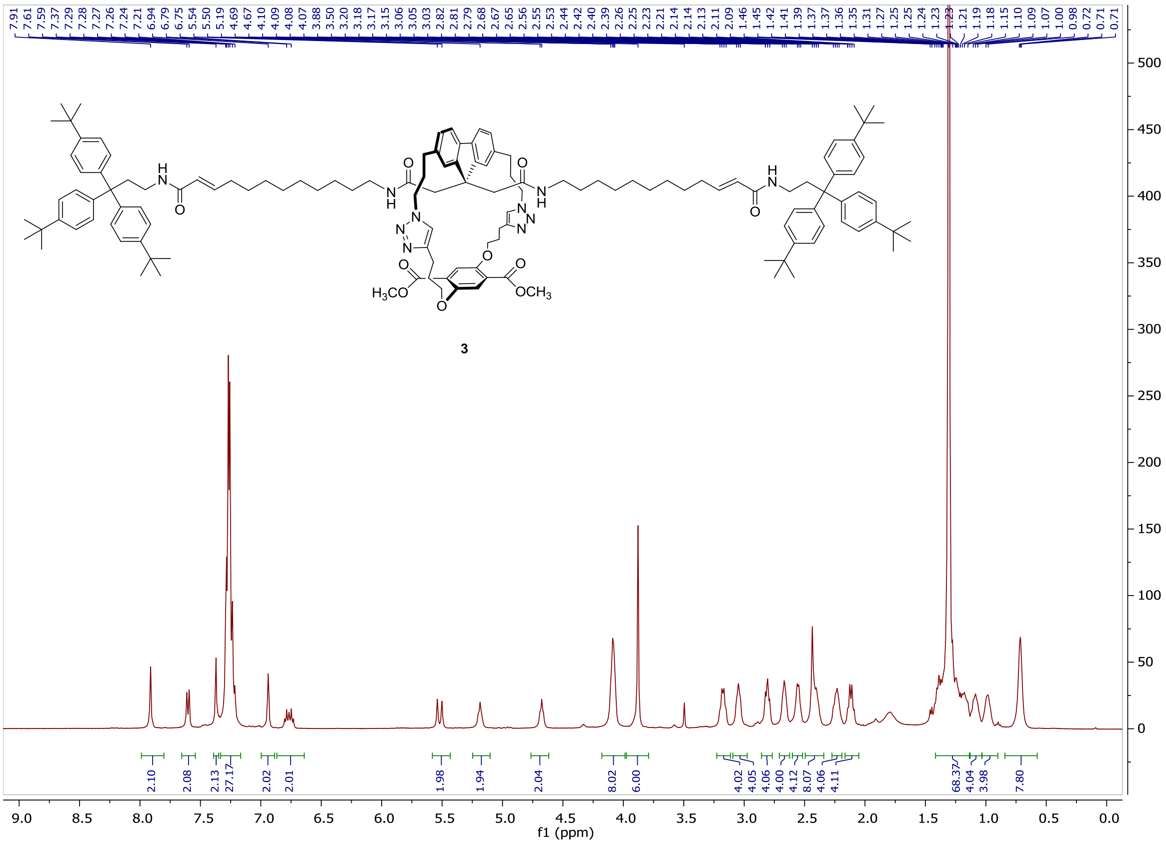

Supplementary figure 67. ^{1}H-NMR spectrum of compound 3 ($CDCl_3$, 500 MHz)

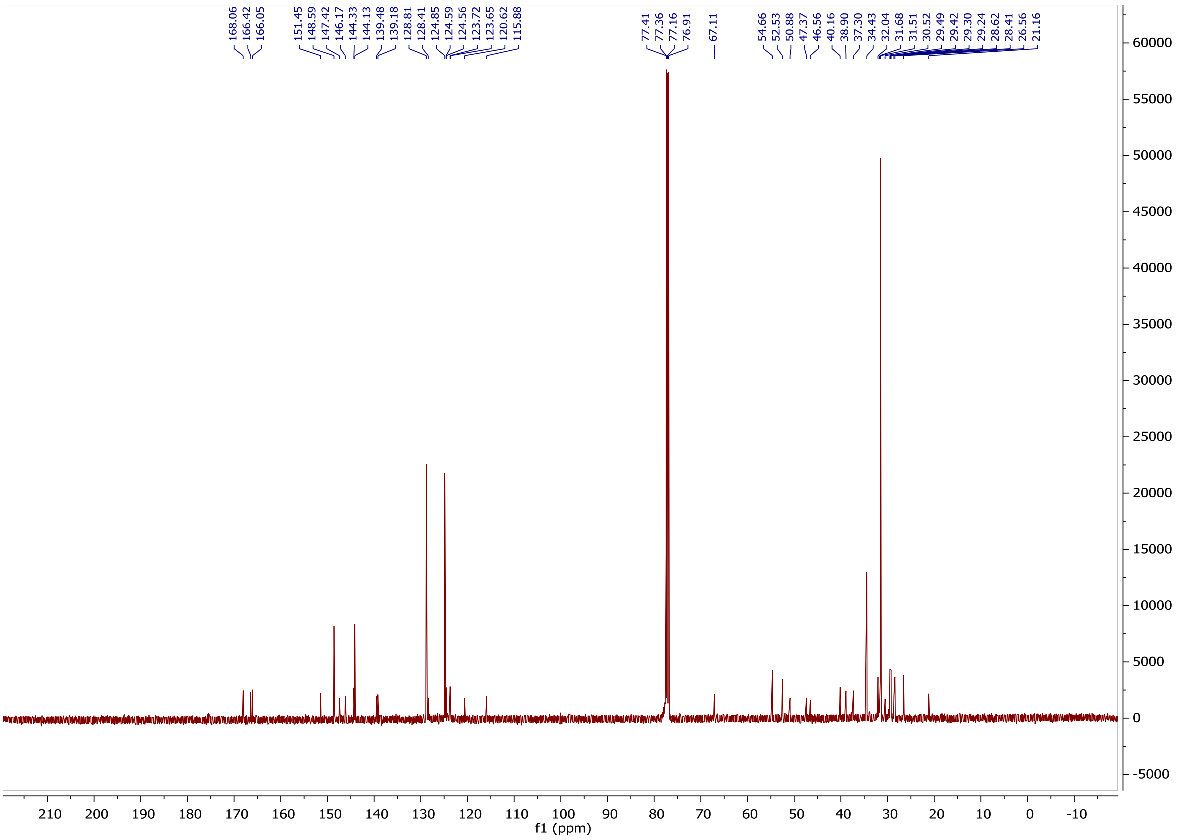

Supplementary figure 68. ^{13}C-NMR spectrum of compound 3 ($CDCl_3$, 125 MHz)

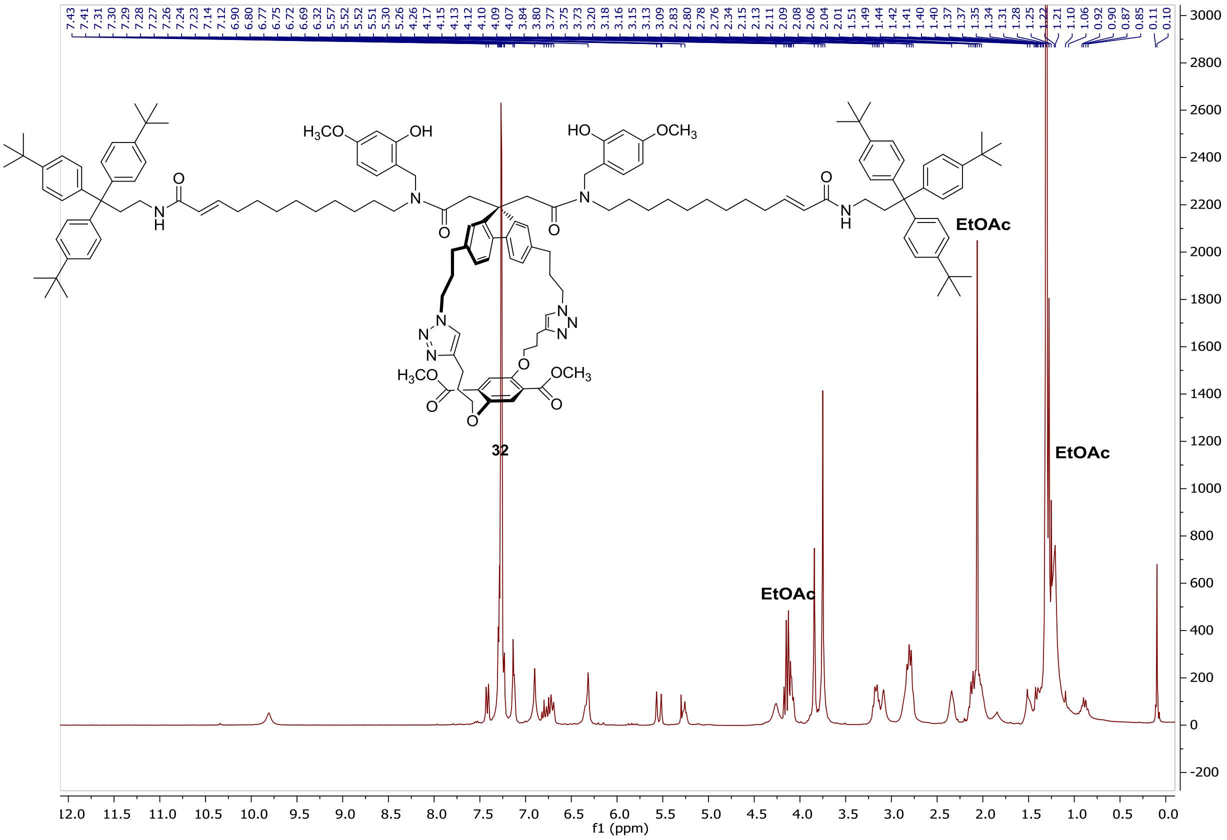

Supplementary figure 69. ^{1}H-NMR spectrum of compound 32 ($CDCl_3$, 300 MHz)

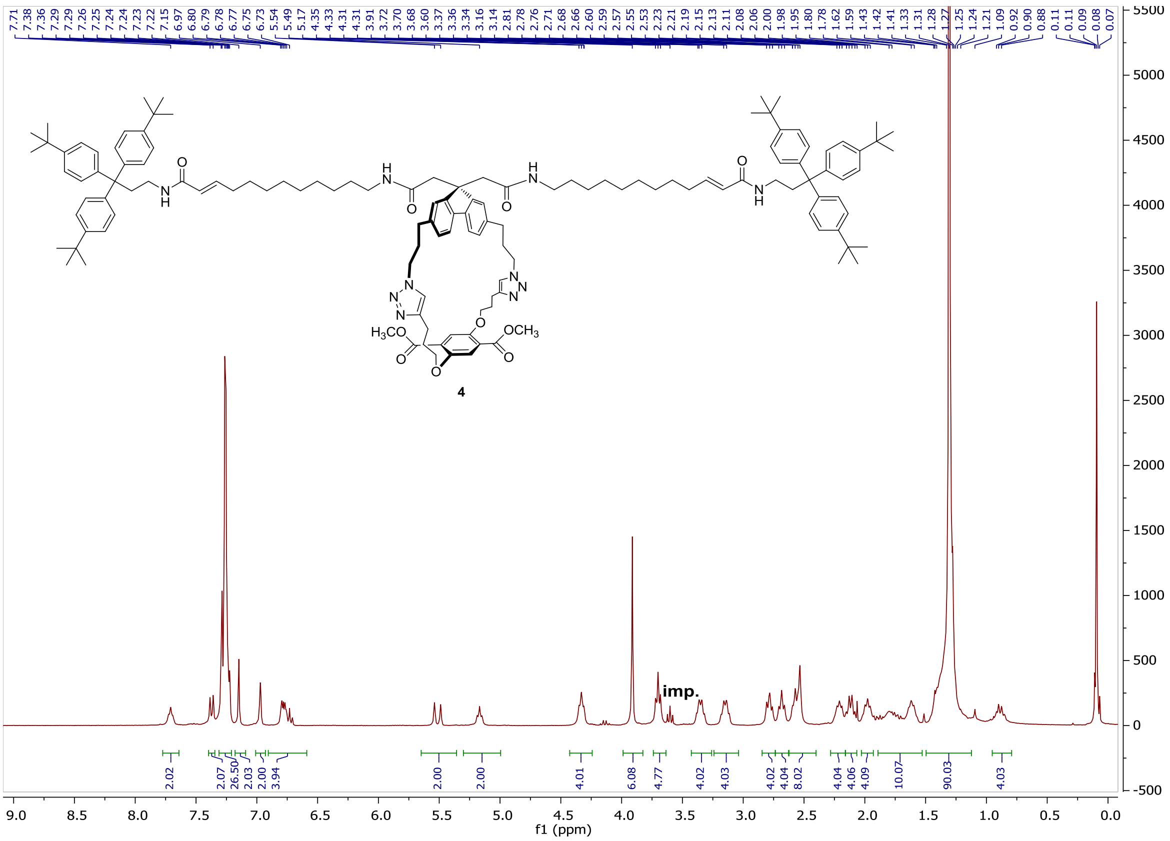

Supplementary figure 70. ^{1}H-NMR spectrum of compound 4 ($CDCl_3$, 500 MHz)

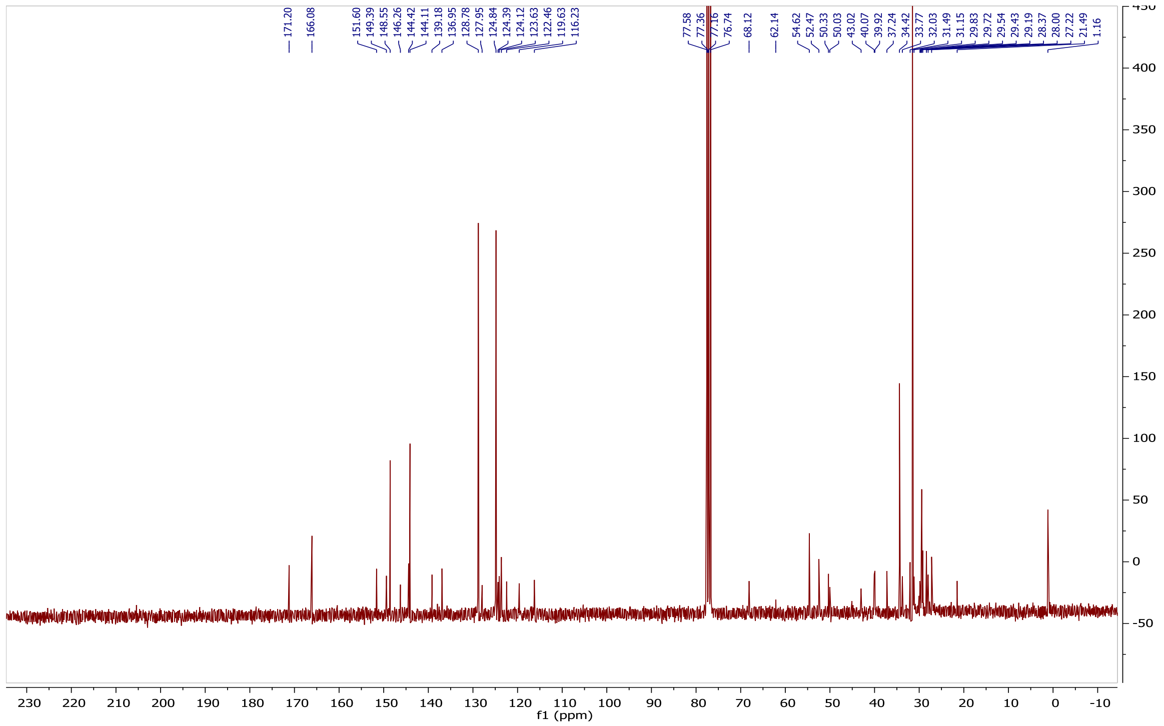

Supplementary figure 71. ^{13}**C-NMR spectrum of compound 4 ($CDCl_3$, 125 MHz)**

Supplementary methods

General information

Unless stated otherwise, reactions were performed without special precautions like drying or N_2/Argon atmosphere. Dried CH_2Cl_2 and CH_3CN were obtained by distilling these solvents with CaH_2 as drying agent. Dried THF and Et_2O were obtained by distillation with sodium. All dried solvents were stored under N_2 atmosphere. Dry DMF on 4 Å molecular sieves was obtained from Sigma-Aldrich and stored under N_2 atmosphere. Reagents were purchased with the highest purity (usually >98%) from Sigma Aldrich and Fluorochem and used as received. Grubbs 2nd generation catalyst was purchased from AK Scientific and TBTA was purchased from TCI Europe. Reactions were monitored with thin layer chromatography (TLC) carried out on 0.25 mm E. Merck silica gel plates (60F-254). SilaFlash® P60 (particle size 40-63 µm) was used for silica column chromatography. NMR spectra were recorded on Bruker DRX-500, 400 and 300 MHz instruments and calibrated on residual undeuterated solvent signals as internal standard. The ^{1}H-NMR multiplicities were abbreviated as followed: s = singlet, d = doublet, t = triplet, q = quartet, quint = quintet, m = multiplet. High resolution mass spectra (HRMS) were recorded on an AccuTOF GC v 4g, JMS-T100GCV Mass spectrometer (JEOL, Japan). FD/FI probe equipped with FD Emitter, Carbotec or Linden (Germany), FD 10 µm. Current rate 51.2 mA/min over 1.2 min machine using field desorption (FD) as ionization method. Depending on the molecule, either the $(M)^+$ or $(M+H)^+$ were observed; often the $(M+Na)^+$ signal was also observed. Melting points were recorded on a Wagner & Munz Polytherm A melting point apparatus and are uncorrected. IR spectra were recorded on a Bruker Alpha FTIR machine. 2,7-diiodofluorene[1] and 3,3,3-tris(tert-butylphenyl)propionic acid[2] were synthesized according to literature procedures. For the purification of quasi-rotaxane **3**, a Waters Prep 100 SFC UV directed system was used; Column: Waters Viridis Prep Silica 2-EP, OBD (100x19 mm, 5 µm), Flow: 70 mL/min, Column temp: 35°C; ABPR: 120 bar; Eluent A: CO_2, Eluent B: 20 mM Ammonia in EtOH, Isocratic: 18 % B for 10 min, Injection: Sandwich 100 µL methanol, Collection: Based on PDA TIC.

Synthetic procedures

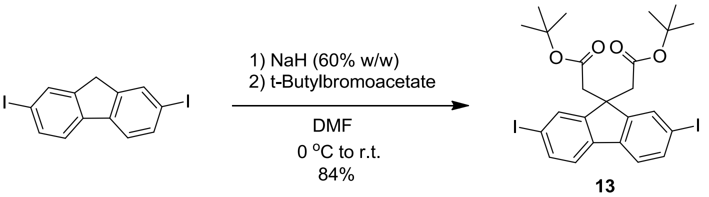

13

Compound **13**

4.18 g 2,7-diiodofluorene[1] (10 mmol) was dissolved in 60 mL dry DMF, under N_2 atmosphere and cooled to 0 °C. After cooling, 880 mg NaH (60% w/w in mineral oil, 22 mmol, 2.2 equiv) was added; the suspension was stirred for 5 minutes and then 3.25 mL tert-butyl bromoacetate (22 mmol, 2.2 equiv) was added dropwise. The reaction was stirred for 1h at 0 °C and overnight at room temperature, and then concentrated *in vacuo*. The residue was partitioned between 100 mL EtOAc and 50 mL 1M HCl. The water layer was extracted with 2 x 50 mL EtOAc and the combined organic layers were washed with 2 x 50 mL H_2O, 50 mL brine, dried over $MgSO_4$ and concentrated *in vacuo*. The crude product was dry-loaded on silica and purified by column chromatography (PE:EtOAc 30:1 → 25:1 → 20:1) to give compound **13** (5.44 g, 8.42 mmol, 84%) as a yellow solid. Melting point: 110-115 °C; ^{1}H-NMR (300 MHz, $CDCl_3$,): δ 7.88 (s, 2H), 7.70 (d, 2H), 7.42 (d, 2H), 2.93 (s, 4H), 1.13 (s, 18H); ^{13}C-NMR (75 MHz, $CDCl_3$) δ 168.80, 150.16, 139.26, 136.98, 133.38, 121.61, 92.90, 80.77, 50.69, 44.09, 27.68; IR (cm^{-1}): 2977, 1717, 1367, 1142

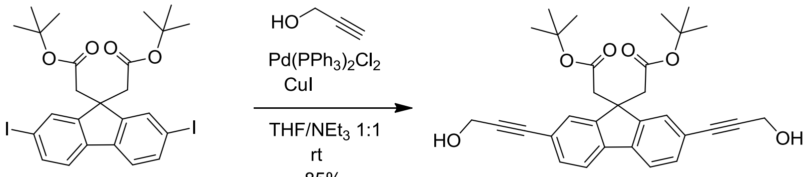

13 **14**

Compound **14**

4.35 g **13** (6.73 mmol) and 1.55 mL propargylalcohol (26.93 mmol, 4 equiv) were dissolved in 60 mL dry THF/NEt_3 1:1 and the mixture was degassed with three vacuum/N_2 cycles. After degassing, 189 mg $Pd(PPh_3)_2Cl_2$ (0.269 mmol, 0.04 equiv) and 102 mg CuI (0.538 mmol, 0.08 equiv) were added. The reaction was stirred overnight at room temperature and concentrated *in vacuo*. The residue was partitioned between 50 mL EtOAc and 50 mL 1M HCl. The water layer was extracted with 2 x 25 mL EtOAc and the combined organic layers were washed with 25 mL brine, dried over $MgSO_4$ and concentrated *in vacuo*. The residue was purified by column chromatography (PE:EtOAc 5:2 → 2:1 → 1:1) to give **14** (2.88 g, 5.73 mmol, 85%) as a beige solid. Melting point: 175-178 °C (dec.); ^{1}H-NMR (300 MHz, $CDCl_3$,): δ = 7.64-7.61 (m, 4H), 7.46 (d, 2H), 4.53 (s, 4H), 2.94 (s, 4H), 2.04 (bs, 2H), 1.03 (s, 18H); ^{13}C-NMR (75 MHz, $CDCl_3$): δ = 168.91, 148.79, 140.36, 131.62, 127.47, 121.69, 120.02, 87.88, 86.21, 80.66, 51.77, 50.50, 44.63, 27.55; IR (cm^{-1}): 3504, 2978, 1697, 1468, 1347, 1151

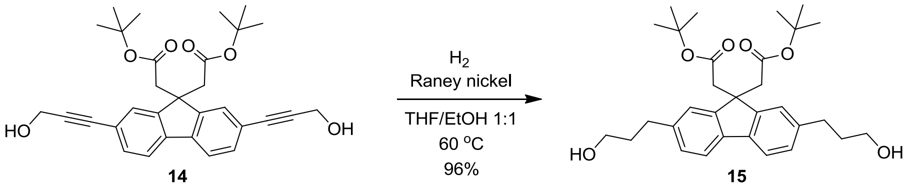

14 **15**

Compound **15**

2.98 g **14** (5.92 mmol) was dissolved in 50 mL THF/EtOH 1:1 and 15 mL of a slurry of Raney nickel in H_2O was added. The mixture was degassed by 5 cycles of vacuum/H_2 and the mixture was stirred overnight at 60 °C under H_2 atmosphere. After completion, the mixture was purged with N_2 gas for 15 minutes, and then filtered over Celite. The filter cake was washed with 2 x 10 mL EtOAc and the combined organic layers were concentrated *in vacuo*. The remaining water layer was partitioned with 20 mL EtOAc and separated. The water layer was extracted with 2 x 10 mL EtOAc and the combined organic layers were washed with 20 mL brine, dried over $MgSO_4$ and concentrated *in vacuo* to give **15** (2.91 g, 5.69 mmol, 96%) as a viscous yellow oil and was used without further purification. ^{1}H-NMR (300 MHz, $CDCl_3$) δ 7.55 (d, 2H), 7.35 (s, 2H), 7.16 (d, 2H), 3.66 (t, 4H), 2.93 (s, 4H), 2.78 (t, 4H), 2.16 (bs, 2H), 1.94 (quint, 4H), 1.01 (s, 18H); ^{13}C-NMR (75 MHz, $CDCl_3$) δ 169.62, 148.52, 140.68, 138.57, 127.89, 124.27, 119.37, 80.21, 61.84, 50.34, 44.97, 34.43, 32.35, 27.51; IR (cm^{-1}): 3394, 2977, 2932, 1718, 1471, 1367, 1155

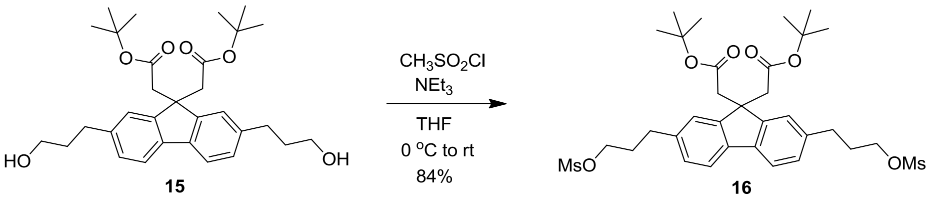

Compound **16**

2.77 g **15** (5.43 mmol) and 2.50 mL Et_3N (17.92 mmol, 3.3 equiv) were dissolved in 25 mL dry THF under N_2 atmopshere, cooled to 0 °C and 1.27 mL methanesulfonylchloride (16.29 mmol, 3 equiv) was added dropwise. The icebath was removed and the reaction was stirred overnight at room temperature, and was subsequently quenched with 5 mL H_2O and stirred for 15 min. The mixture was diluted with 50 mL Et_2O and 50 mL H_2O. The water layer was extracted with 25 mL Et_2O and the combined organic layers were washed with 25 mL 1M HCl, 25 mL brine, dried over $MgSO_4$ and concentrated *in vacuo*. The crude product was purified by column chromatography (PE/EtOAc 4:2 → 3:2 → 2:2) to give **16** (3.04 g, 4.56 mmol, 84%) as a yellow solid. Melting point: 96-100 °C; ^{1}H-NMR (300 MHz, $CDCl_3$) δ 7.57 (d, 2H), 7.34 (s, 2H), 7.16 (d, 2H), 4.25 (t, 4H), 3.03 (s, 6H), 2.93 (s, 4H), 2.82 (t, 4H), 2.10 (quint , 4H), 0.99 (s, 18H); ^{13}C-NMR (75 MHz, $CDCl_3$,) δ 169.18, 148.77, 139.16, 138.83, 127.95, 124.14, 119.60, 80.01, 69.22, 50.38, 44.86, 37.26, 31.72, 30.98, 27.43; IR (cm^{-1}): 2974, 2932, 1714, 1353, 1171

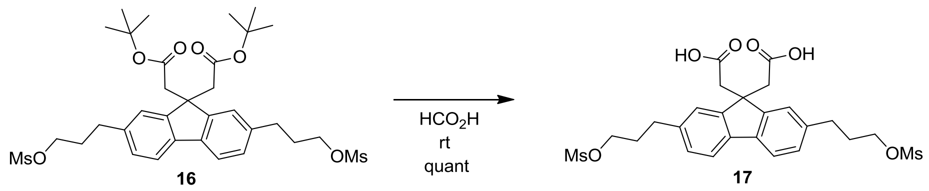

Compound **17**

3.21 g **16** (4.81 mmol) was dissolved in 30 mL HCO_2H and stirred overnight at room temperature. The mixture was concentrated *in vacuo* and dried thoroughly on a high vacuum pump to give **17** (2.66 g 4.80 mmol, quant) as a thick yellow oil. ^{1}H-NMR (300 MHz, $CDCl_3$) δ 7.62 (d, 2H), 7.37 (s, 2H), 7.20 (d, 2H), 4.21 (t, 4H), 3.08 (s, 4H), 3.00 (s, 6H), 2.81 (t, 4H), 2.09 (quint, 4H); ^{13}C-NMR (75 MHz, $CDCl_3$) δ 175.94, 148.63, 139.64, 138.01, 128.60, 124.03, 120.24, 69.08, 49.03, 41.46, 37.36, 31.61, 30.67; IR (cm^{-1}): 3028, 2940, 1708, 1346, 1170

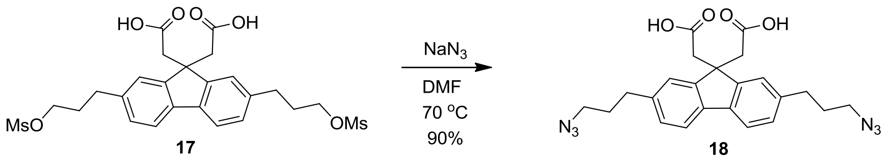

Compound **18**

2.66 g **17** (4.79 mmol) and 1.24 g NaN_3 (19.18 mmol, 4 equiv) were dissolved in 20 mL DMF and stirred overnight at 70 °C. The mixture was cooled to room temperature and diluted with 40 mL Et_2O and 40 mL 1M HCl. The water layer was extracted with 20 mL Et_2O and the combined organic layers were washed with 30 mL brine, dried over $MgSO_4$ and concentrated *in vacuo*. The product was dried further on a high vacuum pump to give **18** (1.94 g, 4.32 mmol, 90%) as a yellow solid. Melting point: 101-104 °C; ^{1}H-NMR (300 MHz, $CDCl_3$) δ 7.62 (d, 2H), 7.39 (s, 2H), 7.20 (d, 2H), 3.27 (t, 4H), 3.10 (s, 4H), 2.77 (t, 4H), 1.92 (quint, 4H); ^{13}C-NMR (75 MHz, $CDCl_3$) δ 176.79, 148.82, 140.31, 137.88, 128.56, 124.08, 120.11, 50.57, 48.89, 41.23, 33.02, 30.62; IR (cm^{-1}): 2933, 2858, 2095, 1695, 1261, 1196

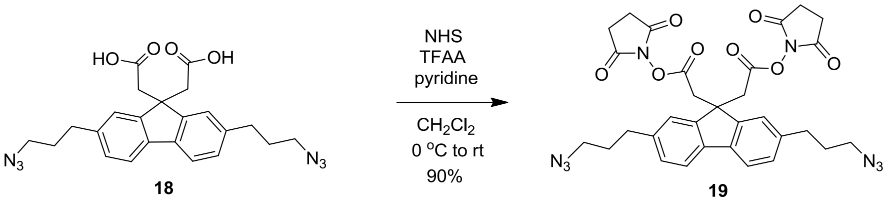

Compound **19**

For the synthesis of bis-OSu ester **19** the protocol of Brunckova *et al* was used.[3]: 1.93 g **18** (4.30 mmol) was dissolved in 40 mL dry CH_2Cl_2 under N_2 atmosphere and cooled to 0 °C, after which 3.47 mL pyridine (43 mmol, 10 equiv) and 2.03 g *N*-hydroxysuccinimide (17.63 mmol, 4.1 equiv) were added, followed by dropwise addition of 2.39 mL TFAA (17.20 mmol, 4 equiv). The mixture was stirred overnight at room temperature and quenched by addition of 20 mL 1M HCl and stirred for 15 min. The water layer was extracted with 2 x 10 mL CH_2Cl_2 and the combined organic layers were washed with 20 mL 1M HCl and 2 x 20 mL $NaHCO_3$, dried over $MgSO_4$ and concentrated in *vacuo*. The crude product was purified by column chromatography (PE/EtOAc 3:2 → 2:3 → 2:4). The product was dried on a high vacuum pump to give **19** (2.47 g, 3.85 mmol, 90%) as a colorless foam. ^{1}H-NMR (300 MHz, $CDCl_3$) δ 7.65 (d, 2H), 7.50 (s, 2H), 7.25 (d, 2H), 3.37 (s, 4H), 3.33 (t, 4H), 2.82-2.78 (m, 12H), 1.97 (quint, 4H); ^{13}C-NMR (75 MHz, $CDCl_3$) δ

169.00, 165.92, 147.13, 140.84, 137.72, 129.09, 124.17, 120.11, 50.56, 48.59, 38.30, 32.87, 30.39, 25.60; IR (cm^{-1}): 2944, 2865, 2094, 1814, 1784, 1734, 1162, 1061

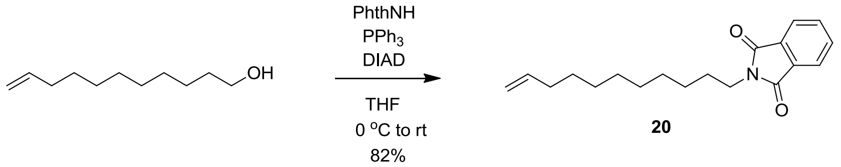

Compound **20**

6.01 mL 10-undecylalcohol (30.0 mmol), 7.87 g PPh_3 (30.0 mmol, 1 equiv) and 4.41 g phthalimide (30.0 mmol, 1 equiv) were dissolved in 150 mL dry THF under N_2 atmosphere and cooled to 0 °C, followed by dropwise addition of 5.91 mL DIAD (30.0 mmol, 1 equiv). The reaction was stirred overnight at room temperature and the mixture was concentrated *in vacuo* and loaded on silica. The dry-loaded product was purified by column chromatography (PE/EtOAc 30:1 → 25:1) to give a slightly yellow oil, which slowly crystallized to give **20** (7.38 g, 24.6 mmol, 82%) as a waxy solid. Melting point: 39-40 °C; ^{1}H-NMR (300 MHz, $CDCl_3$) δ 7.82 (m, 2H), 7.71 (m, 2H), 5.81 (m, 1H), 4.95 (dd, 2H), 3.67 (t, 2H), 2.02 (q, 2H), 1.67 (quint, 2H), 1.33-1.27 (m, 12H); ^{13}C-NMR (75 MHz, $CDCl_3$) δ 168.52, 139.27, 133.89. 132.26, 123.21, 114.19, 38.14, 33.87, 29.49, 29.45, 29.15, 28.97, 28.67, 26.93; IR (cm^{-1}): 2916, 2848, 1693, 1398, 1052

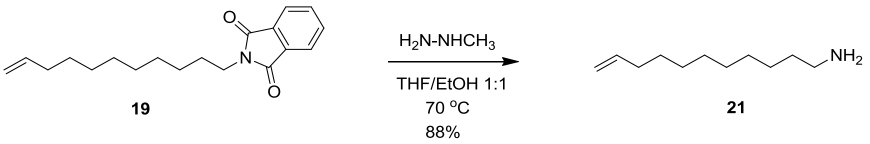

Compound **21**

7.31 g **20** (24.4 mmol) was dissolved in 100 mL THF/EtOH 1:1 and 2.57 mL methylhydrazine (48.9 mmol, 2 equiv) was added. The mixture was stirred overnight at 70 °C and the mixture was concentrated *in vacuo* and to the residue was added 100 mL Et_2O. The precipitate was filtered off and washed with Et_2O. The combined organic layers were concentrated *in vacuo* and the crude product was purified by Kugelrohr distillation (0.02 mbar, 90-110 °C) to give **21** (3.66 g, 21.6 mmol, 88%) as a clear colorless oil. ^{1}H-NMR (300 MHz, $CDCl_3$) δ 5.78 (m, 1H), 4.93 (dd, 2H), 2.65 (t, 2H), 2.02 (q, 2H), 1.40-1.26 (m, 16H); ^{13}C-NMR (75 MHz, $CDCl_3$) δ 139.23, 114.14, 42.31, 33.92, 33.85, 29.62, 29.54, 29.49, 29.17, 28.97, 26.93; IR (cm^{-1}): 3333, 2920, 2852, 1568, 1487

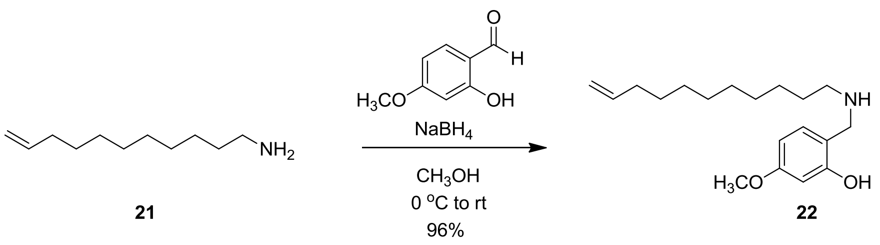

Compound **22**

1.02 g **21** (6.00 mmol) and 913 mg 2-hydroxy-4-methoxybenzaldehyde (6.00 mmol, 1 equiv) were dissolved in 30 mL absolute CH_3OH and stirred overnight at room temperature. The mixture was cooled to 0 °C and 454 mg $NaBH_4$ (12.0 mmol, 2 equiv) was added in one portion. The reaction was stirred for 1h at 0 °C, 1h at room temperature and then concentrated *in vacuo*. The residue was partitioned between 40 mL EtOAc and 20 mL $NaHCO_3$. The water layer was extracted with 2 x 10 mL EtOAc and the combined organic layers were washed with 20 mL brine, dried over $MgSO_4$ and concentrated *in vacuo* to give **22** (1.77 g, 5.78 mmol, 96%) as a faint yellow oil, which was used without further purification. ^{1}H-NMR (300 MHz, $CDCl_3$) δ 6.88 (d, 1H), 6.44 (s, 1H), 6.35 (s, 1H), 5.82 (m, 1H), 4.97 (dd, 2H), 3.95 (s, 2H), 3.78 (s, 3H), 2.68 (t, 2H), 2.06 (q, 2H), 1.54 (quint, 2H), 1.39-1.30 (m, 12H); ^{13}C-NMR (75 MHz, $CDCl_3$) δ 160.46, 159.62, 139.34, 128.78, 115.05, 114.25, 104.89, 102.02, 55.34, 52.28, 48.73, 33.92, 29.68, 29.60, 29.53, 29.21, 29.03, 27.25; IR (cm^{-1}): 2923, 2852, 1622, 1590, 1510, 1456, 1157

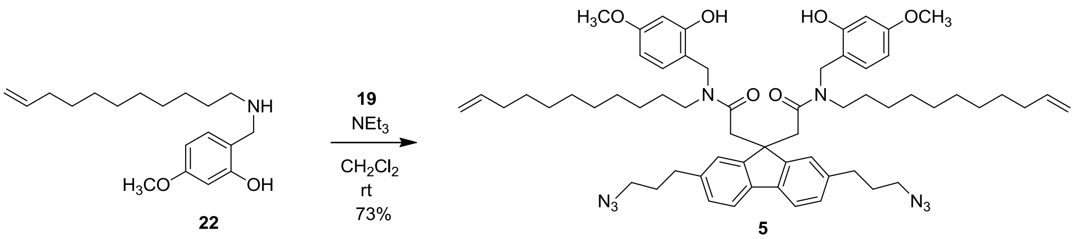

Compound **5**

1.80 g **19** (2.80 mmol), 1.76 g **22** (5.75 mmol, 2.05 equiv) and 0.93 mL Et_3N (6.71 mmol, 2.4 equiv) were dissolved in 17 mL dry CH_2Cl_2 and the mixture was stirred for 3 days at room temperature. The reaction mixture was washed with 10 mL 1M HCl and the water layer was extracted with 10 mL CH_2Cl_2. The combined organic layers were washed with 10 mL $NaHCO_3$, dried over $MgSO_4$ and concentrated *in vacuo*. The crude product was purified by column chromatography (PE/EtOAc 6:1 → 5:1) to give a colorless oil, which slowly crystallized to give **5** (2.10 g, 2.05 mmol, 73%) as a colorless waxy solid. Melting point: 81-82 °C; ^{1}H-NMR (300 MHz, $CDCl_3$) δ 10.15 (s, 2H), 7.60 (d, 2H), 7.28 (s, 2H), 7.13 (d, 2H), 6.96 (d, 2H), 6.54 (s, 2H), 6.38 (d, 2H), 5.83 (m, 2H), 4.99 (dd, 4H), 4.39 (bs, 4H), 3.82 (s, 6H), 3.18 (m, 8H), 2.90 (t, 4H), 2.43 (t, 4H), 2.08 (q, 4H), 1.73 (quint, 4H), 1.46-1.12 (m, 30H); ^{13}C-NMR (75 MHz, $CDCl_3$) δ 173.56, 161.50, 157.82, 150.09, 140.53, 139.22, 137.30, 132.29, 128.11, 124.23, 119.87, 114.95, 114.24, 105.19, 102.66, 55.31, 50.88, 50.67, 47.51, 46.23, 37.80, 33.85, 32.82, 30.53, 29.43, 29.12, 28.95, 27.77, 26.69; IR (cm^{-1}): 2925, 2854, 2094, 1595, 1466, 1158

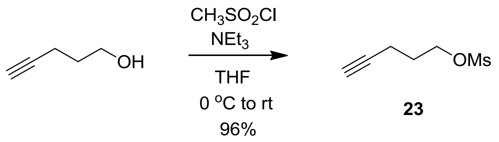

Compound **23**

1.86 mL 4-pentyn-1-ol (20.0 mmol) and 3.33 mL Et_3N (24.0 mmol, 1.2 equiv) were dissolved in 40 mL dry THF and cooled to 0 °C and 1.70 mL methanesulfonyl chloride (22.0 mmol, 1.1 equiv) was added dropwise. The icebath was removed and the reaction was stirred overnight at room temperature and was then quenched with 20 mL H_2O and stirred for 15 min. The mixture was diluted with 40 mL Et_2O and 20 mL 1M HCl and the water layer was extracted with 2 x 20 mL Et_2O. The combined organic layers were washed with 40 mL brine, dried over $MgSO_4$ and concentrated *in vacuo* to give **23** (3.11 g, 19.18 mmol, 96%) as a slightly yellow oil, which was used without further purification. ^{1}H-NMR (300 MHz, $CDCl_3$) δ 4.37 (t, 2H), 3.05 (s, 3H), 2.38 (dt, 2H), 2.03 (t, 1H), 1.98 (quint, 2H); ^{13}C-NMR (75 MHz, $CDCl_3$) δ 82.21, 69.92, 68.38, 37.37, 27.87, 14.79; IR (cm^{-1}): 3287, 3029, 2940, 1346, 1331, 1168

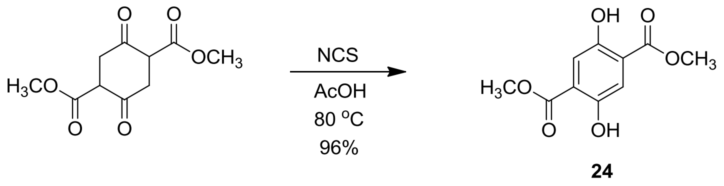

Compound **24**

5.70 g dimethyl-2,5-dioxocyclohexane-1,4-dicarboxylate (25.0 mmol) was suspended in 25 mL AcOH and heated to 80 °C and 3.41 g *N*-chlorosuccinimide (25.5 mmol, 1.02 equiv) was added portionwise over 30 minutes. The reaction was stirred at 80 °C for 90 minutes and was then cooled to room temperature and diluted with 25 mL H_2O. The solid was filtered and washed with 2 x 25 mL H_2O, 2 x 5 mL CH_3OH and dried on air and vacuum to give **24** (5.43 g, 24.0 mmol, 96%) as a yellow powder. Melting point: 173-175 °C; ^{1}H-NMR (300 MHz, $CDCl_3$) δ 10.05 (s, 2H), 7.45 (s, 2H), 3.97 (s, 6H); ^{13}C-NMR (75 MHz, $CDCl_3$) δ 169.57, 152.99, 118.40, 117.84, 52.88; IR (cm^{-1}): 3234, 2957, 1672, 1440, 1328, 1193, 1172

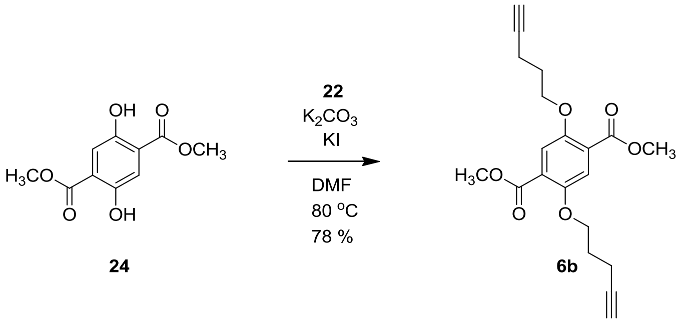

Compound **6b**

2.93 g **23** (18.0 mmol, 2.4 equiv), 1.70 g **24** (7.52 mmol, 1.0 equiv), 2.49 g K_2CO_3 (18.0 mmol, 2.4 equiv) and 124 mg KI (0.75 mmol, 0.1 equiv) were dissolved in 50 mL dry DMF and stirred overnight at 80 °C. The mixture was cooled to room temperature, concentrated *in vacuo* and to the residue was added EtOAc and the suspension was filtered. The filter cake was washed with EtOAc and the combined organic layers were washed with 2 x 20 mL H_2O and 20 mL brine, dried over $MgSO_4$ and concentrated *in vacuo*. The crude product was purified by column chromatography (PE/EtOAc 4:1) to give **6b** (2.11 g, 5.89 mmol, 78%) as a slightly yellow solid. Note: the product is slightly contaminated (see ^{1}H-NMR spectrum at page 51) with the methyl-pentynol-ester. This has no consequence for the next step

(saponification). Melting point: 89-93 °C; ^{1}H-NMR (300 MHz, $CDCl_3$) δ 7.42 (s, 2H), 4.14 (t, 4H), 3.92 (s, 6H), 2.47 (dt, 4H), 2.06 (quint, 4H), 1.98 (t, 2H); ^{13}C-NMR (75 MHz, $CDCl_3$) δ 166.15, 151.92, 124.59, 117.04, 83.57, 69.04, 68.16, 52.46, 28.33, 15.18; IR (cm^{-1}): 3299, 2884, 2116, 1725, 1504, 1408, 1201

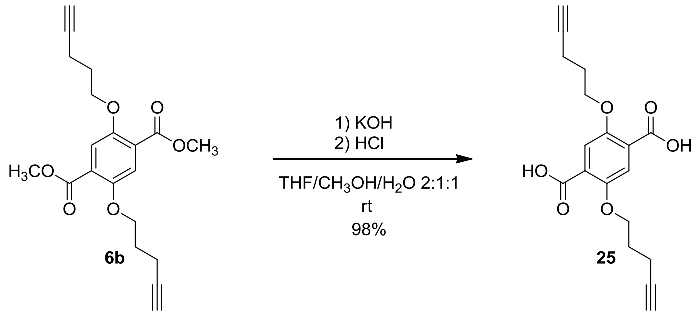

Compound **25**

1.44 g **6b** (4.02 mmol) was dissolved in 30 mL $THF/CH_3OH/H_2O$ 2:1:1 and 901 mg KOH (15.1 mmol, 4 equiv) was added. The mixture was stirred overnight at room temperature and was acidified with 3 mL 37% HCl and stirred for 15 minutes. The mixture was diluted with 20 mL H_2O and 20 mL EtOAc. The water layer was extracted with 3 x 20 mL EtOAc and the combined organic layers were washed with 20 mL brine, dried over $MgSO_4$ and concentrated *in vacuo* to give **24** (1.30 g, 3.94 mmol, 98%) as a faint yellow solid. Melting point: 175-178 °C; Yield: ^{1}H-NMR (300 MHz, CD_3OD) δ 7.48 (s, 2H), 4.17 (t, 4H), 2.45 (dt, 4H), 2.25 (t, 2H), 2.02 (quint, 4H); ^{13}C-NMR (75 MHz, CD_3OD) δ 168.75, 152.91, 126.30, 117.60, 84.14, 70.04, 69.34, 29.42, 15.72; IR (cm^{-1}): 3267, 2938, 2875, 1673, 1505, 1217

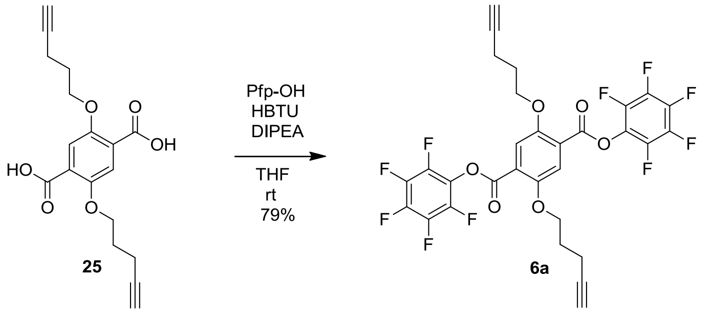

Compound **6a**

1.30 g **25** (3.94 mmol), 2.17 g pentafluorophenol (11.8 mmol, 3 equiv), 4.11 mL DIPEA (23.6 mmol, 6 equiv) and 4.48 g HBTU (11.8 mmol, 3 equiv) were dissolved in 80 mL dry THF and stirred overnight at room temperature. The mixture was concentrated *in vacuo* and dry-loaded on silica and purified by column chromatography (PE/EtOAc 10:1 → 8:1 → 6:1) to give **6a** (2.07 g, 3.13 mmol, 79%) as an off-white solid. Melting point: 113-115 °C; ^{1}H-NMR (300 MHz, $CDCl_3$) δ 7.67 (s, 2H), 4.26 (t, 4H), 2.46 (dt, 4H), 2.08 (quint, 4H), 1.98 (t, 2H); ^{13}C-NMR (75 MHz, $CDCl_3$) δ 160.92, 152.90, 143.20, 139.84, 138.34, 136.50, 122.35, 117.31, 83.21, 69.24, 68.17, 28.13, 15.08; IR (cm^{-1}): 3316, 2943, 1755, 1519, 1416, 1390, 1230, 1201

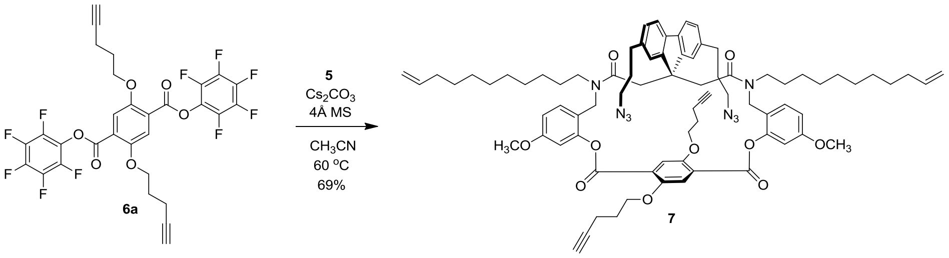

Compound **7**

1.94 g **5** (1.89 mmol), 1.38 g **6a** (2.08 mmol, 1.1 equiv), 2.45 g Cs_2CO_3 (7.58 mmol, 4 equiv) and 1.00 g 4 Å MS were dissolved in 750 mL dry CH_3CN and the mixture was stirred overnight at 60 °C under N_2 atmosphere. The solvent was evaporated and the residue was taken in ca. 20 mL CH_2Cl_2 and filtered through a plug of Celite, which was washed with CH_2Cl_2, The organic layer was concentrated in *vacuo* and dry-loaded on silica and purified by column chromatography (PE:EtOAc 4:1 → 3:1) to give **7** (1.72 g, 1.31 mmol, 69%) as a thick colorless oil. Due to the complexity of the ^{1}H-NMR spectrum, please consult supplementary figure 37; IR (cm^{-1}): 2924, 2853, 2093, 1740, 1637, 1618, 1504, 1410, 1183, 1108; HRMS (FD) calcd for $C_{79}H_{96}N_8O_{10}$ [M$^+$]: 1316.7249, found: 1316.7239

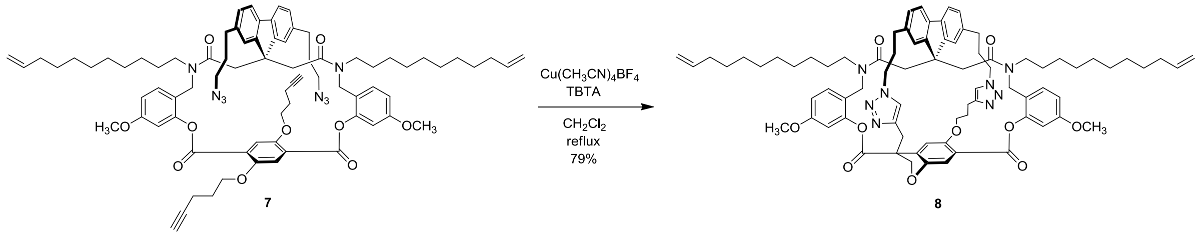

Compound **8**

For the CuAAC reaction the ligand TBTA was used, as first described by Sharpless *et al.*[4]: 420 mg **7** (0.319 mmol) and 42 mg TBTA (0.080 mmol, 0.25 equiv) were dissolved in 65 mL dry CH_2Cl_2 and degassed with five vacuum/N_2 cycles, after which 25 mg $Cu(CH_3CN)_4BF_4$ (0.080 mmol, 0.25 equiv) was added and the mixture was stirred overnight at reflux under N_2 atmosphere. The reaction mixture was concentrated in *vacuo* and dry-loaded on silica and purified by column chromatography (PE:EtOAc 1:1 → 1:2 → 0:1) to give **8** (333 mg, 0.253 mmol, 79%) as an a colorless foam. Melting point: 113-119 °C; Due to the complexity of the ^{1}H-NMR spectrum, please consult supplementary figure 38; IR (cm^{-1}): 2925, 2853, 1746, 1640, 1617, 1504, 1411, 1187, 1100; HRMS (FD) calcd for $C_{79}H_{96}N_8O_{10}$ [M$^+$]: 1316.7249, found: 1316.7243

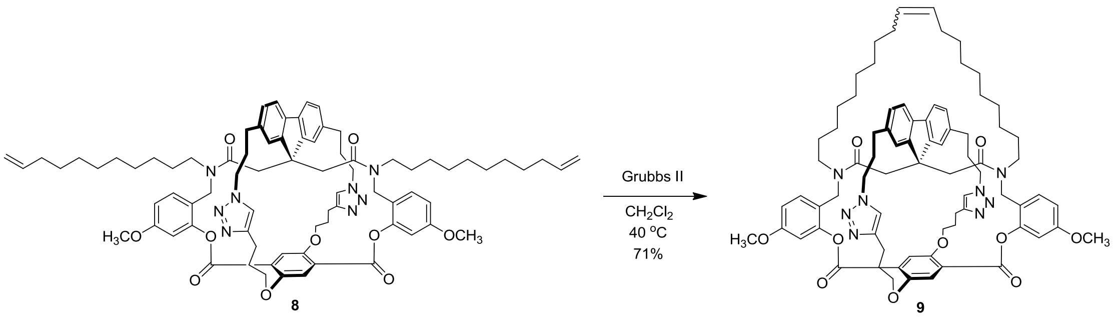

Compound **9**

79 mg **8** (0.060 mmol) was dissolved in 60 mL dry CH_2Cl_2 and degassed with five vacuum / N_2 cycles. To the solution was added 10 mg Grubbs II catalyst (0.012 mmol, 0.2 equiv) and the mixture was stirred overnight at 40 °C. The mixture was concentrated in *vacuo*, dry-loaded on silica and purified by column chromatography (PE:EtOAc 1:2 → 1:3 → 1:4) to give **9** (55 mg, 0.043 mmol, 71%) as a beige solid. Due to the complexity of the ^{1}H-NMR spectrum, please consult supplementary figure 39; IR (cm^{-1}): 2924, 2853, 1749, 1618, 1505, 1411, 1187, 1111; HRMS (FD) calcd for $C_{77}H_{92}N_8O_{10}$ [M$^+$]: 1288.6936, found: 1288.6911

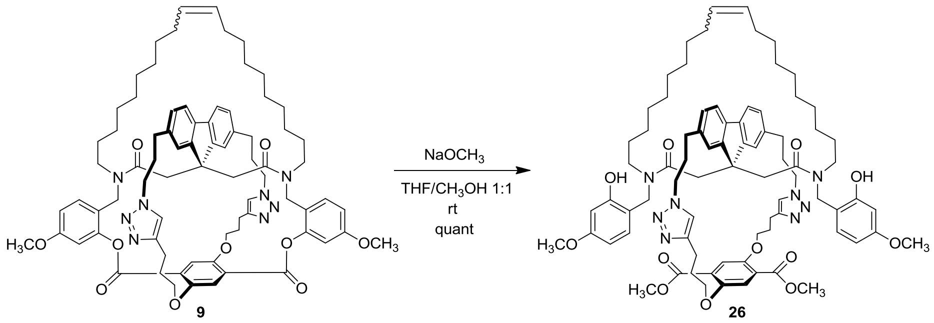

Compound **26**

55 mg **9** (0.043 mmol) was dissolved in 2 mL dry THF/CH_3OH 1:1 and 12 mg anhydrous $NaOCH_3$ (0.21 mmol, 5 equiv) was added and the mixture was stirred at room temperature for 1h. The reaction was quenched by addition of 0.5 mL AcOH and the reaction was diluted with 15 mL EtOAc and 10 mL saturated $NaHCO_3$. The water layer was extracted with 2 x 5 mL EtOAc and the combined organic layers were dried over $MgSO_4$ and concentrated in *vacuo* to give **26** (58 mg , 0.043 mmol, quant) as a slight yellow film. Due to the complexity of the ^{1}H-NMR spectrum, please consult supplementary figure 40; IR (cm^{-1}): 2923, 2853, 1726, 1619, 1599, 1436, 1203; HRMS (FD) calcd for $C_{79}H_{101}N_8O_{12}$ [(M+H)$^+$]: 1353.7539, found: 1353.7597

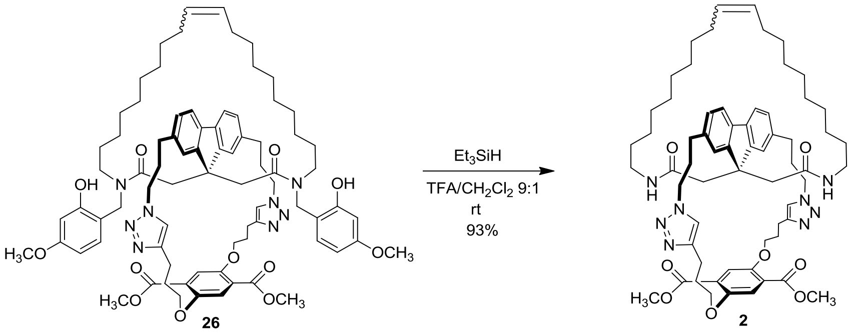

Compound **2**

58 mg **26** (0.043 mmol) was dissolved in 5 mL TFA/CH_2Cl_2 9:1 and 0.136 mL Et_3SiH (0.854 mmol, 20 equiv) was added. The mixture was stirred overnight at room temperature and concentrated in *vacuo*. The residue was dissolved in 10 mL CH_2Cl_2 and 0.5 mL NEt_3 was added and stirred for 5 minutes. The organic layer was washed with 10 mL 1M HCl, 10 mL $NaHCO_3$, dried over $MgSO_4$ and concentrated in *vacuo*. The crude product was dry-loaded on silica and purified by column chromatography (CH_2Cl_2/ CH_3OH 96:4 → 94:6) to give **2** (43 mg, 0.0397 mmol, 93%) as a colorless foam. ^{1}H-NMR (300 MHz, $CDCl_3$) δ 7.98 (s, 2H), 7.57 (d, 2H), 7.35 (s, 2H), 7.21 (d, 2H), 6.95 (s, 2H), 5.52 (m, 2H), 4.49 (t, 2H), 4.07 (m, 8H), 3.85 (s, 6H), 3.05 (t, 4H), 2.67 (t, 4H), 2.52 (q, 4H), 2.44 (s, 4H), 2.41 (m, 4H), 2.21 (quint, 4H), 1.43 (quint, 4H), 1.35 (quint, 4H), 1.22 (sext, 4H), 1.05 (quint, 4H), 0.92 (sext, 4H), 0.66-0.42 (m, 8H); ^{13}C-NMR (75 MHz, $CDCl_3$) δ 167.66, 166.18, 151.40, 146.77, 146.07, 139.61, 139.17, 130.52, 128.52, 124.59, 124.38, 123.55, 120.51, 115.81, 66.89, 52.34, 50.87, 47.12, 38.70, 32.58, 31.57, 30.41, 29.65, 29.55, 29.36, 29.23, 29.10, 28.97, 28.52, 26.28, 20.96; IR (cm^{-1}): 3325, 2925, 2853, 1726, 1659, 1436, 1205; HRMS (FD) calcd for $C_{63}H_{84}N_8O_8$ [M^+]: 1080.6412, found: 1080.6432

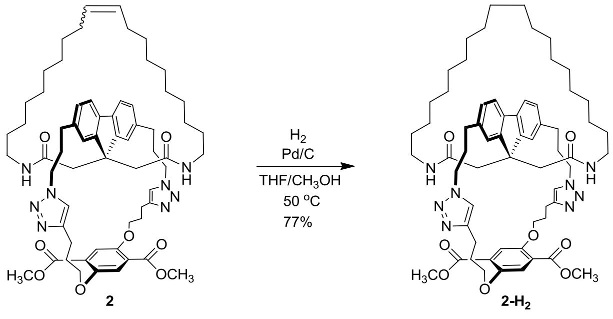

Compound **2-H$_2$**

40 mg **2** (0.037 mmol) was dissolved in 3 mL THF/EtOH 1:1 and 20 mg Pd/C (10 % w/w) was added. Through the solution was bubbled H_2 gas (balloon) for 5 minutes and the reaction was stirred overnight under H_2 atmosphere at 50 °C. The mixture was filtered through a plug of Celite and concentrated in *vacuo*. The crude product was dry-loaded on silica and purified by column chromatography (CH_2Cl_2/CH_3OH 96:4 → 94:6) to give **2-H$_2$** (31 mg, 0.029 mmol, 77%) as a colorless solid.

A crystal was grown in the following way: The purified product was dissolved in ca. 0.5 mL EtOAc and transferred to a test tube, which was put in a closed container filled with pentane, allowing for diffusion of the solvents. After standing overnight, small crystals had formed on the walls of the test tube, which were suitable for the X-ray analysis.

Melting point: 164-166 °C; ^{1}H-NMR (300 MHz, $CDCl_3$) δ 7.98 (s, 2H), 7.59 (d, 2H), 7.36 (s, 2H), 7.23 (d, 2H), 6.96 (s, 2H), 4.50 (t, 2H), 4.09 (m, 8H), 3.86 (s, 6H), 3.06 (t, 4H), 2.68 (t, 4H), 2.53 (q, 4H), 2.44-2.38 (m, 8H), 2.22 (quint, 4H), 1.44-1.34 (m, 16H), 1.22 (quint, 4H), 1.08 (quint, 4H), 0.95 (quint, 4H), 0.67-0.48 (m, 8H); ^{13}C-NMR (75 MHz, $CDCl_3$) δ 167.69, 166.32, 151.50, 146.91, 146.20, 139.77, 139.30, 128.60, 124.71, 124.48, 123.67, 120.56, 115.90, 67.00, 52.47, 51.03, 47.26, 47.14, 38.78, 31.69, 30.53, 29.46, 29.44, 29.31, 29.16, 29.04, 28.93, 28.81, 28.60, 26.31, 21.06; IR (cm^{-1}): 3413, 2925, 2853, 1727, 1660, 1436, 1207; HRMS (FD) calcd for $C_{63}H_{86}N_8O_8$ [M$^+$]: 1082.6569, found: 1082.6533

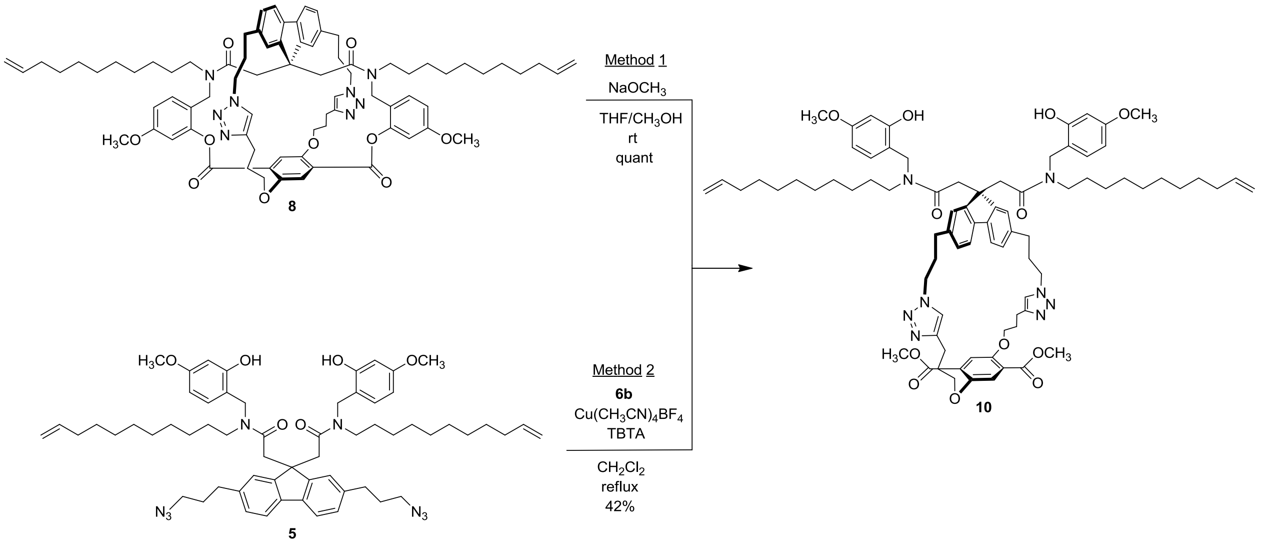

Compound **10**

Method 1: 129 mg **8** (0.0980 mmol) was dissolved in 4 mL dry THF/CH_3OH 1:1 and 105 mg $NaOCH_3$ (1.96 mmol, 20 equiv) was added. The reaction was stirred for at room temperature for 2h and was quenched with 0.2 mL AcOH. The mixture was diluted with 15 mL EtOAc and 10 mL H_2O. The water layer was extracted with 2 x 5 mL EtOAc and the combined organic layers were washed with 10 mL saturated NaCl, dried over $MgSO_4$ and concentrated in *vacuo* to give **10** (138 mg, 0.0980 mmol, quant) as a colorless film.

Method 2: 112 mg **5** (0.110 mmol, 1.1 equiv), 36 mg **6b** (0.10 mmol) and 11 mg TBTA (0.020 mmol, 0.2 equiv) were dissolved in 100 mL dry CH_2Cl_2 and the mixture was degassed with five vacuum/N_2 cycles. After degassing, 6 mg $Cu(CH_3CN)_4BF_4$ (0.02 mmol, 0.2 equiv) was added and the mixture was stirred overnight at reflux under N_2 atmosphere. The reaction was concentrated *in vacuo* and dry-loaded on silica and purified by column chromatography (PE:EtOAc 1:2 → 1:3) to give **10** (58 mg, 0.042 mmol, 42%) as a colorless film. Due to the complexity of the ^{1}H-NMR spectrum, please consult supplementary figure 49; IR (cm^{-1}): 2926, 2854, 1726, 1600, 1505, 1437, 1202; HRMS (FD) calcd for $C_{81}H_{104}N_8O_{12}$ [(M+H)$^+$]: 1381.8, found: 1381.5

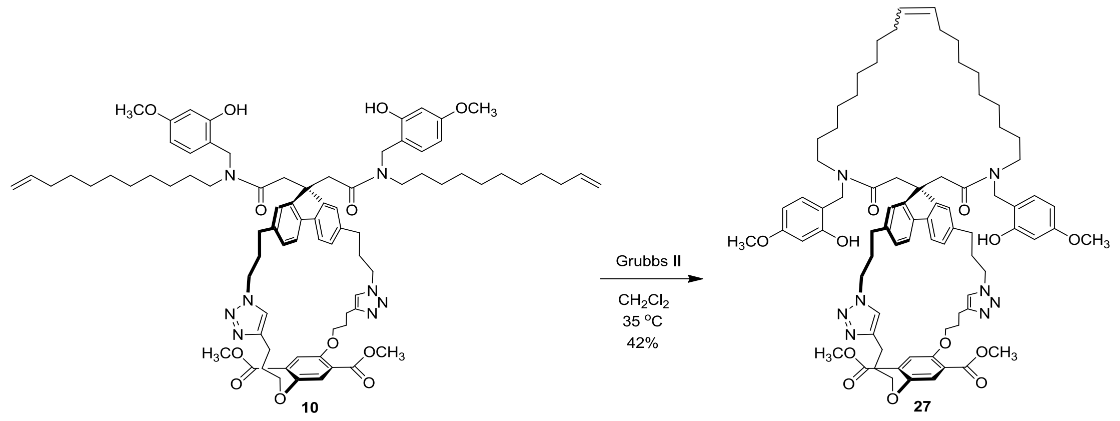

Compound **27**

151 mg **10** (0.109 mmol) was dissolved in 110 mL dry CH_2Cl_2 and was degassed with five vacuum/N_2 cycles. After degassing, 18 mg Grubbs II catalyst (0.021 mmol, 0.2 equiv) was added and the mixture was stirred overnight at 35 °C. The mixture concentrated *in vacuo* and the crude product was dry-loaded on silica and purified by column chromatography (PE/EtOAc 1:3 → 1:5 → 1:7 → 0:1) to give **27** (62 mg, 0.046 mmol, 42%) as a slightly brown film. Due to the complexity of the ^{1}H-NMR spectrum, please consult supplementary figure 50; IR (cm^{-1}): 2923, 2852, 1728, 1599, 1435, 1201; HRMS (FD) calcd for $C_{79}H_{101}N_8O_{12}$ [(M+H)$^+$]: 1353.7539; found: 1353.7556

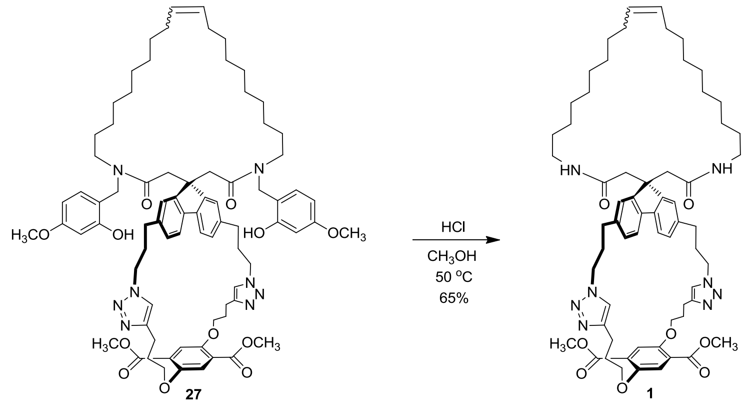

Compound **1**

62 mg **27** (0.046 mmol) was dissolved in 1 mL dry CH_3OH and added dropwise to a 5 mL 3M HCl in CH_3OH solution (made via addition of acetyl chloride to CH_3OH at 0 °C). The reaction was stirred overnight at 50 °C and was concentrated in *vacuo*. The residue was partitioned between 20 mL CH_2Cl_2 and 15 mL $NaHCO_3$. The water layer was extracted with 2 x 5 mL CH_2Cl_2 and the combined organic layers were dried over $MgSO_4$ and concentrated in *vacuo*. The crude product was dry-loaded on silica and purified by column chromatography (EtOAc/CH_3OH 97:3 → 96:4) to give **1** (32 mg, 0.030 mmol, 65%) as a colorless film. ^{1}H-NMR (300 MHz, $CDCl_3$) δ 7.84 (t, 2H), 7.39 (d, 2H), 7.22 (s, 2H), 7, 15 (s, 2H), 6.96 (s, 2H), 6.78 (d, 2H), 5.37 (s, 2H), 4.35 (t, 4H), 3.93 (s, 6H), 3.71 (t, 4H), 3.40 (q, 4H), 2.69 (t, 4H), 2.57 (t, 4H), 2.53 (s, 4H), 2.20 (quint, 4H), 2.07-1.94 (m, 8H), 1.91-1.81 (m, 8H), 1.63 (quint, 4H), 1.48-1.25 (m, 32H); ^{13}C-NMR (75 MHz, $CDCl_3$) δ 171.26, 166.07, 151.62, 149.46, 139.17, 136.91, 130.75, 127.91, 124.40, 124.13, 119.54, 116.26, 68.19, 52.45, 50.25, 50.10, 42.84, 39.77, 33.84, 32.29, 31.19, 29.65, 29.53, 29.43, 29.04, 28.35, 27.94, 27.27, 27.09, 26.93, 26.41, 26.24, 21.50; IR (cm^{-1}): 3256, 2925, 2853, 1727, 1637, 1436, 1206; HRMS (FD) calcd for $C_{63}H_{84}N_8O_8$ [M$^+$]: 1080.6412; found: 1080.6391

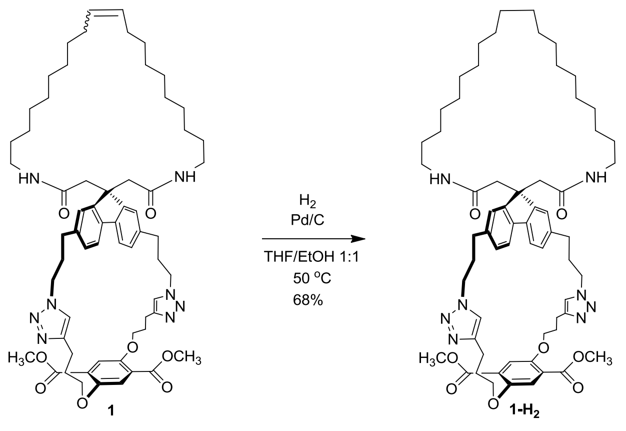

Compound **1-H₂**

19 mg **1** (0.018 mmol) was dissolved in 2 mL THF/EtOH 1:1 and 8 mg Pd/C (10% w/w) was added. H_2 gas (balloon) was bubbled through the solution for 5 minutes, and the reaction was stirred overnight at 50 ^oC under H_2 atmosphere. The solution was filtered through a plug of Celite (washed with EtOH) and the combined organic layers were concentrated in *vacuo*. The crude product was dry-loaded on silica and purified by column chromatography (EtOAc/CH_3OH 96:4) to give **1-H₂** (13 mg, 0.012 mmol, 68%) as a colorless film. ^{1}H-NMR (300 MHz, $CDCl_3$) δ 7.79 (t, 2H), 7.39 (d, 2H), 7.22 (s, 2H), 7.15 (s, 2H), 6.96 (s, 2H), 6.77 (d, 2H), 4.35 (t, 4H), 3.93 (s, 6H), 3.70 (t, 4H), 3.39 (q, 4H), 2.68 (t, 4H), 2.57 (t, 4H), 2.53 (s, 4H), 2.26 (quint, 4H), 2.03-1.60 (m, 16H), 1.45-1.27 (m, 52H); ^{13}C-NMR (125 MHz, $CDCl_3$) δ 171.27, 166.09, 151.64, 149.46, 146.25, 139.21, 136.94, 127.95, 124.41, 124.15, 122.46, 119.57, 116.28, 68.21, 52.49, 50.32, 50.11, 42.94, 39.91, 35.64, 35.16, 33.87, 32.07, 31.23, 29.84, 29.59, 29.21, 29.12, 29.08, 28.83, 28.58, 28.53, 28.29, 27.99, 27.10, 27.00, 26.45, 26.42, 26.26, 22.83, 21.52; IR (cm^{-1}): 3262, 2924, 2853, 1728, 1637, 1410, 1206; HRMS (FD) calcd for $C_{63}H_{86}N_8O_8$ [M^+]: 1082.6569; found: 1082.6559

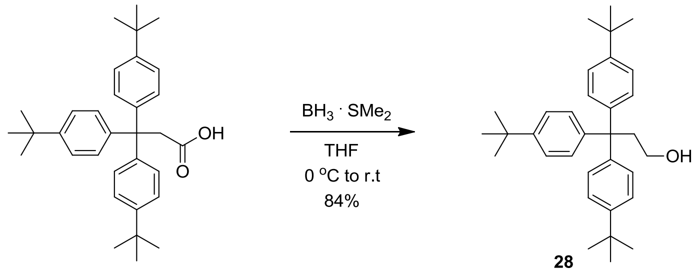

Compound **28**

6.20 g 3,3,3-tris(4-tertbutylphenyl)propionic acid[2] (13.2 mmol) was dissolved in 100 mL dry THF under N_2 atmosphere and cooled to 0 °C. After cooling, 3.12 mL BH_3•SMe_2 (32.9 mmol, 2.5 equiv) was added dropwise and the icebath was removed. The reaction was stirred overnight at room temperature and was quenched carefully with 10 mL H_2O and stirred for 15 minutes. The mixture was concentrated in *vacuo* and the residue was partitioned between 150 mL Et_2O and 75 mL H_2O. The water layer was extracted with 30 mL Et_2O and the combined organic layers were washed with 75 mL brine and dried over $MgSO_4$ and concentrated in *vacuo*. The residue was purified by column chromatography (CH_2Cl_2) to give **28** (5.07 g, 11.1 mmol, 84%) as a colorless foam. Melting point: 98 – 110 °C; ^{1}H-NMR (400 MHz, $CDCl_3$) δ 7.31 (d, 6H), 7.24 (d, 6H), 3.54 (t, 2H), 2.93 (t, 2H), 1.35 (s, 27H); ^{13}C-NMR (100 MHz, $CDCl_3$) δ 148.55, 144.32, 128.65, 124.80, 60.84, 54.08, 43.13, 34.41, 31.51; IR (cm^{-1}): 3340, 2957, 2901, 2866, 1508, 1362, 1269

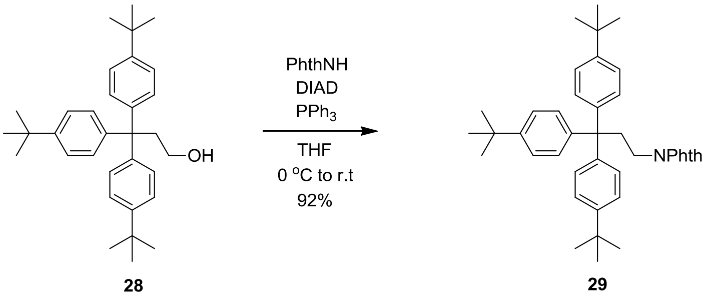

Compound **29**

2.73 g **28** (5.98 mmol), 1.32 g phthalimide (8.97 mmol, 1.5 equiv) and 1.80 g PPh_3 (6.88 mmol, 1.15 equiv) were dissolved in 60 mL dry THF under N_2 atmosphere and cooled to 0 °C. After cooling, 1.30 mL DIAD (6.58 mmol, 1.1 equiv) was added dropwise and the mixture was stirred at 0 °C for 2 h and overnight at room temperature. The mixture was concentrated in *vacuo,* dry-loaded on silica and purified by column chromatography (PE/EtOAc 8:1) to give **29** (3.24 g, 5.53 mmol, 92%) as a white powder. Melting-point: 256 – 264 °C; ^{1}H-NMR (400 MHz, $CDCl_3$) δ 7.84 (m, 2H), 7.72 (m, 2H), 7.33 (d, 6H), 7.28 (d, 6H), 3.47 (t, 2H), 2.93 (t, 2H), 1.32 (s, 27H); ^{13}C-NMR (100 MHz, $CDCl_3$) δ 168.38, 148.60, 143.85, 133.94, 132.35, 128.78, 124.83, 123.15, 54.18, 38.88, 36.24, 34.40, 31.48; IR (cm^{-1}): 2961, 2902, 2866, 1711, 1397, 1360

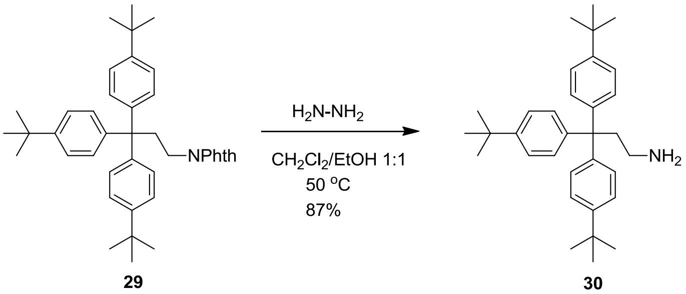

Compound **30**

2.32 g **29** (3.97 mmol) was dissolved in a mixture of 40 mL CH_2Cl_2/EtOH 1:1 and 1.93 mL hydrazine hydrate (39.7 mmol, 10 equiv) was added and the reaction was stirred at 50 °C for 3h. The mixture was cooled to room temperature and filtered (cake washed with 10 mL CH_2Cl_2) and the filtrate was concentrated in *vacuo*. The crude product was dry-loaded on silica and purified by column chromatography (CH_2Cl_2/CH_3OH 94:6 → 90:10) to give **30** (1.58 g, 3.46 mmol, 87%) as a colorless solid. Melting-point: 151 - 158 °C; ^{1}H-NMR (300 MHz, $CDCl_3$) δ 7.28 (d, 6H), 7.19 (d, 6H), 2.78 (t, 2H), 2.55 (t, 2H), 1.31 (s, 27H); ^{13}C-NMR (75 MHz, $CDCl_3$) δ 148.37, 144.53, 128.72, 124.66, 54.56, 44.48, 39.33, 34.38, 31.49; IR (cm^{-1}): 2958, 2901, 2866, 1507, 1362, 1269

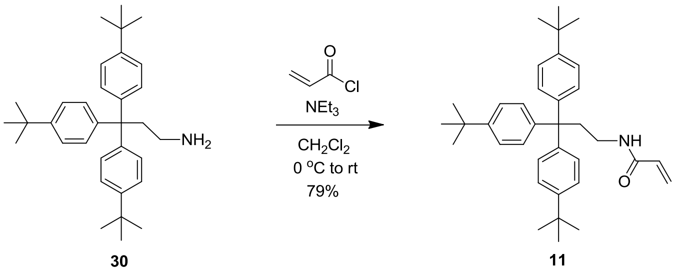

Compound **11**

455 mg **30** (1.00 mmol) and 0.166 mL NEt_3 (1.20 mmol, 1.2 equiv) were dissolved in 10 mL dry CH_2Cl_2 and cooled to 0 °C. After cooling, 0.097 mL acryloyl chloride (1.1 mmol, 1.1 equiv) was added dropwise and the mixture was stirred for 1h at 0 °C and overnight at room temperature. The organic layer was washed with 10 mL 1M HCl and the water layer was extracted with 5 mL CH_2Cl_2. The combined organic layers were dried over $MgSO_4$ and concentrated in *vacuo*. The crude product was purified by column chromatography (CH_2Cl_2) to give **11** (400 mg, 0.790 mmol, 79%) as a colorless powder. Melting point: 254-256 °C; ^{1}H-NMR (300 MHz, $CDCl_3$) δ 7.29 (d, 6H), 7.25 (d, 6H), 6.17 (d, 1H), 5.85 (dd, 1H), 5.53 (d, 1H), 5.22 (t, 1H), 3.20 (q, 2H), 2.82 (t, 2H), 1.32 (s, 27H); ^{13}C-NMR (75 MHz, $CDCl_3$) δ 165.47, 148.64, 144.06, 130.98, 128.77, 125.98, 124.89, 54.65, 40.00, 37.31, 34.42, 31.49; IR (cm^{-1}): 3279, 2953, 2902, 2866, 1656, 1555

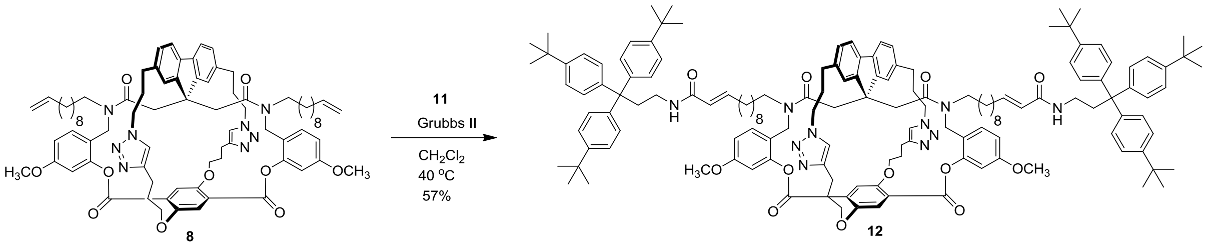

Compound **12**

66 mg **8** (0.050 mmol) and 102 mg **11** (0.200 mmol, 4 equiv) were dissolved in 10 mL dry CH_2Cl_2 and the mixture was degassed with 5 vacuum/N_2 cycles. After degassing, 9 mg Grubbs II catalyst (0.01 mmol, 0.2 equiv) was added and the mixture was stirred overnight at 40 °C. The solution was concentrated in *vacuo* and dry-loaded on silica and purified by column chromatography (PE/EtOAc 1:1 → 1:2) to give **12** (65 mg, 0.029 mmol, 57%) as a faint yellow solid film. Melting point: 153-160 °C; Due to the complexity of the ^{1}H-NMR spectrum, please consult supplementary figure 65; IR (cm^{-1}): 3293, 2961, 2928, 2858, 1746, 1668, 1618, 1506, 1201; HRMS (FD) calcd for $C_{147}H_{183}N_{10}O_{12}$ [(M+H)$^+$]: 2280.4017, found 2280.4065

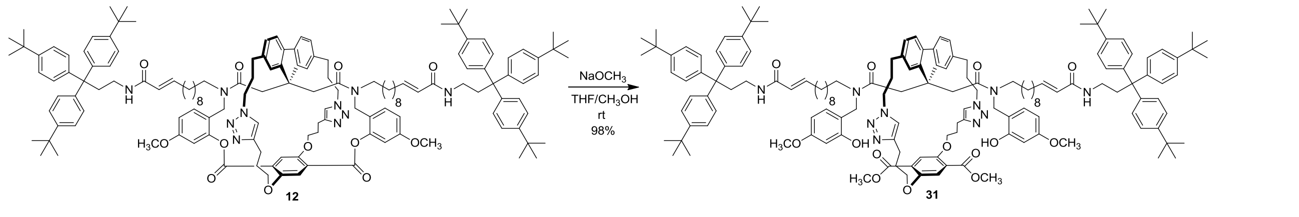

Compound **31**

65 mg **12** (0.029 mmol) was dissolved in 2 mL THF/CH_3OH 3:1 and 16 mg $NaOCH_3$ (0.29 mmol, 10 equiv) was added and the reaction was stirred at room temperature for 2h and was subsequently quenched with 0.1 mL AcOH. The reaction was diluted with 20 mL CH_2Cl_2 and 10 mL $NaHCO_3$. The water layer was extracted with 2 x 5 mL CH_2Cl_2 and the combined organic layers were dried over $MgSO_4$ and concentrated in *vacuo* to give **31** (66 mg, 0.028 mmol, 98%) as a faint yellow glassy solid. Melting point: 133-140 ^{o}C; Due to the complexity of the ^{1}H-NMR spectrum, please consult supplementary figure 66; IR (cm^{-1}): 2960, 2928, 2857, 1726, 1669, 1622, 1601, 1507, 1203; HRMS (FD) calcd for $C_{149}H_{191}N_{10}O_{14}$ [(M+H)$^{+}$]: 2344.4541, found: 2344.4529

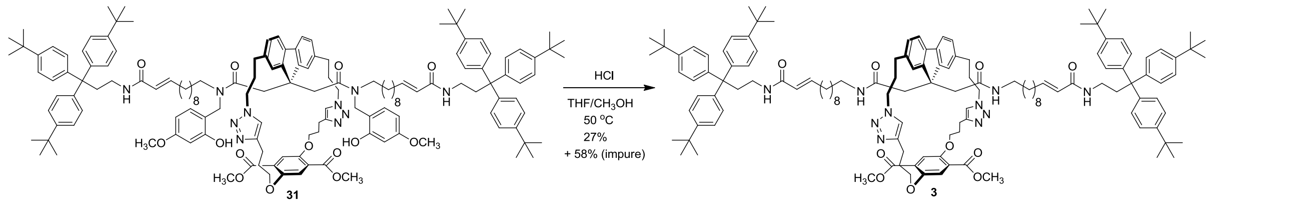

Compound **3**

66 mg **31** (0.028 mmol) was dissolved in 1 mL THF and added dropwise to 4 mL 3M HCl in CH_3OH (made via addition of acetyl chloride to CH_3OH at 0 ^{o}C). The reaction was stirred overnight at 50 ^{o}C and was subsequently concentrated in *vacuo*. The residue was partitioned between 20 mL CH_2Cl_2 and 15 mL $NaHCO_3$ and the water layer was extracted with 2 x 5 mL CH_2Cl_2. The combined organic layers were dried over $MgSO_4$ and concentrated in *vacuo*. The crude product was dry-loaded on silica and purified by column chromatography (EtOAc/CH_3OH 99:1 → 98:2 → 97:3) to give **3** (16 mg, 0.0077 mmol, 27%, together with 34 mg mixed fractions (< 0.016 mmol, < 58%)) as a faint yellow solid.

Note: two products are formed, which do separate on TLC, but badly on column resulting in only a limited amount of pure fractions of the desired product. To obtain analytically pure material, the mixed fractions of above were subjected to supercritical preparative HPLC using liquid CO_2 as an eluent (see general methods for details) to give a white powder. Melting point: 106-111°C (stays thick and oily); ^{1}H-NMR (400 MHz, $CDCl_3$) δ 7.92 (s, 2H), 7.61 (d, 2H), 7.37 (s, 2H), 7.30-7.21 (m, 26H), 6.94 (s, 2H), 6.78 (dt, 2H), 5.53 (d, 2H), 5.18 (t, 2H), 4.68 (t, 2H), 4.08 (m, 8H), 3.89 (s, 6H), 3.18 (q, 4H), 3.05 (t, 4H), 2.81 (t, 4H), 2.56 (q, 4H), 2.44 (m, 8H), 2.24 (quint, 4H), 2.12 (q, 4H), 1.46-1.18 (m, 68H), 1.09 (quint, 4H), 0.98 (quint, 4H), 0.70 (bs, 8H); ^{13}C-NMR (125 MHz, $CDCl_3$) δ 168.04, 166.42, 166.06, 151.44, 148.58, 147.38, 146.16, 144.38, 144.12, 139.47, 139.19, 128.80, 128.41, 124.86, 124.58, 124.51, 123.72, 123.68, 120.64, 115.84, 67.04, 54.64, 52.55, 50.86, 47.35, 46.56, 40.11, 38.89, 37.28, 34.44, 32.05, 31.66, 31.51, 30.51, 29.50, 29.43, 29.31, 29.24, 28.60, 28.41, 26.56, 21.13; IR (cm^{-1}): 3294, 2957, 2926, 2856, 1726, 1665, 1630, 1507, 1206; HRMS (FD) calcd for $C_{133}H_{175}N_{10}O_{10}$ [(M+H)$^{+}$]: 2072.3493, found 2072.3424

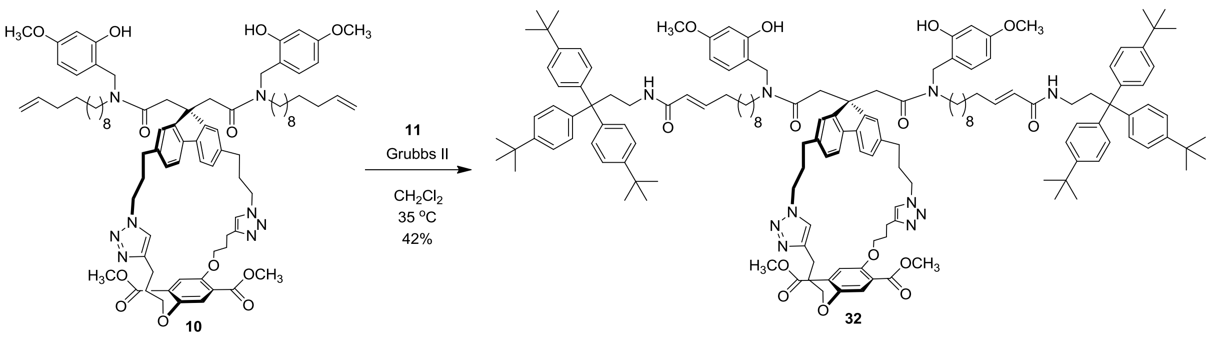

Compound **32**

58 mg **10** (0.042 mmol) and 51 mg **11** (0.10 mmol, 2.4 equiv) were dissolved in 4 mL dry CH_2Cl_2 and the mixture was degassed with five vacuum/N_2 cycles. After degassing, 7 mg Grubbs II catalyst (0.008 mmol, 0.2 equiv) was added and the mixture was stirred overnight at 35 °C. The solution was concentrated in *vacuo* and dry-loaded on silica and purified by column chromatography (PE/EtOAc 1:2 → 1:3) to give **32** (41 mg, 0.018 mmol, 42%) as a faint yellow solid film. Melting trajectory: 140-156 °C (stays thick and oily); Due to the complexity of the ^{1}H-NMR spectrum, please consult supplementary figure 69; IR (cm^{-1}): 2959, 2928, 2856, 1729, 1670, 1622, 1601, 1506; HRMS (FD) calcd for $C_{149}H_{191}N_{10}O_{14}$ [(M+H)$^+$]: 2344.4541, found: 2344.4423

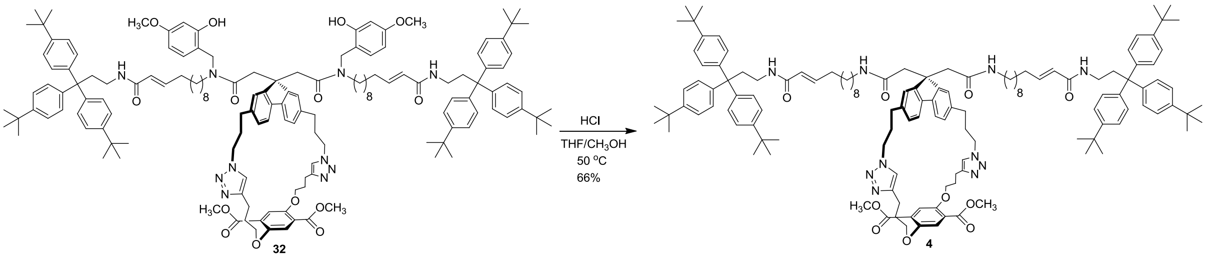

Compound **4**

41 mg **32** (0.018 mmol) was dissolved in 1 mL THF and added dropwise to 3 mL 3M HCl in CH_3OH (made via addition of acetyl chloride to CH_3OH at 0 °C). The reaction was stirred overnight at 50 °C and was subsequently concentrated in *vacuo*. The residue was partitioned between 20 mL CH_2Cl_2 and 15 mL $NaHCO_3$ and the water layer was extracted with 2 x 5 mL CH_2Cl_2. The combined organic layers were dried over $MgSO_4$ and concentrated in *vacuo*. The crude product was dry-loaded on silica and purified by column chromatography (PE/EtOAc/CH_3OH 1:2:0 → 1:4:0 → 1:6:0 →0:1:0 → 0:97:3) to give **4** (24 mg, 0.012 mmol, 66%) as a faint yellow solid film. Melting trajectory: 130-138 °C (stays thick and oily); ^{1}H-NMR (300 MHz, $CDCl_3$) δ 7.71 (t, 2H), 7.37 (d, 2H), 7.29-7.22 (m, 26H), 7.15 (s, 2H), 6.97 (s, 2H), 6.80-6.73 (m, 4H), 5.51 (d, 2H), 5.17 (t, 2H), 4.33 (t, 4H), 3.91 (s, 6H), 3.70+3.60 (dt, 6H), 3.69 (q, 4H), 3.36 (q, 4H), 2.78 (t, 4H), 2.68 (t, 4H), 2.60-2.53 (m, 8H), 2.19 (quint, 4H), 2.12 (q, 4H), 1.95 (quint, 4H), 1.80-1.59 (m, 10H), 1.43-1.21 (m, 90H); ^{13}C-NMR (75 MHz, $CDCl_3$) δ 171.20, 166.08, 151.60, 149.39, 148.55, 146.26, 144.42, 144.11, 139.18, 136.95, 128.78, 127.95, 124.84, 124.39, 124.12, 123.63, 122.46, 119.63, 116.23, 68.12, 54.62, 52.47, 50.33, 50.03, 43.02, 40.07, 39.92, 37.24, 34.42, 33.77, 32.03, 31.49, 31.15, 29.83, 29.72, 29.54, 29.43, 29.19, 28.37, 28.00, 27.22, 21.49; IR (cm^{-1}): 3291, 2961, 2928, 2856, 1726, 1667, 1632, 1507, 1206; HRMS (FD) calcd $C_{133}H_{175}N_{10}O_{10}$ [(M+H)$^+$]: 2072.3493, found: 2072.3566

Supplementary References

1) Harris, J.D., Lui, J., Carter, K.R. Synthesis of π-bridge dually-dopable conjugated polymers from benzimidazole and fluorene: separating sterics from electronics *Macromolecules*, **48**, 6970-6977 (2015)

2) Barlan, A.U., Basak, A., Yamamoto, H. Enantioselective oxidation of olefins catalyzed by a chiral bishydroxamic acid complex of molybdenum, *Angew. Chem. Int. Ed.* **45**, 5849-5852 (2006)

3) Leonard, N.M., Brunckova J. In situ formation of N-trifluoroacetoxy succinimide (TFA-NHS): one-pot formation of succinimidyl esters, N-trifluoroacetyl amino acid succinimidyl esters, and N-maleoyl amino acid succinimidyl esters *J. Org. Chem.*, **76**, 9169-9174 (2011)

4) Chan, T.R., Hilgraf, R., Sharpless, K.B., Fokin, V.V. Polytriazoles as copper(I)-stabilizing ligands in catalysis *Org. Lett.* , **6**, 2853-2855 (2004)
